# Comparative analysis of chemical elements and metabolites in diverse garlic varieties based on metabolomics and ionomics

**DOI:** 10.1002/fsn3.4397

**Published:** 2024-08-07

**Authors:** Junjun Meng, Haitao Zhong, Xue Chu, Jinxiu Guo, Shiyuan Zhao, Tao Shen, Wenxue Sun, Jianhua Wang, Pei Jiang

**Affiliations:** ^1^ Translational Pharmaceutical Laboratory, Jining No. 1 People's Hospital Shandong First Medical University Jining China; ^2^ Shandong Engineering Research Center for Traditional Chinese Medicine Standard, School of Pharmaceutical Sciences, Cheeloo College of Medicine Shandong University Jinan China

**Keywords:** functional component, garlic, ionomics, metabolomics, quality evaluation

## Abstract

As a plant classified under the “medicine food homology” concept, garlic offers various health benefits and comes in many different varieties. In this study, the metabolite composition of different garlic varieties were analyzed using LC–MS/MS quadrupole‐Orbitrap mass spectrometry and ICP‐MS. A total of 30 chemical elements and 1256 metabolites were identified. Significant differences in chemical elements and metabolomics profiles were observed among the five garlic groups (VIP > 1.5). Compared to WG, PG contained 5 unique compounds, HG had 15 unique compounds, SCG had 18 unique compounds, and SBG had 26 unique compounds. Furthermore, the results showed that WG had smaller differences with PG and HG, but significant differences with SBG and SCG. KEGG analysis revealed metabolic pathways associated with the formation of differential metabolites. These findings uncover the differences and mechanisms in the composition of various garlic varieties, providing a theoretical foundation for distinguishing the nutritional components of different garlic types.

## Graphical abstract

The compositional profiles of five garlic varieties were studied and compared. A panel of 1256 metabolites were putatively annotated in garlic by metabolomics. 30 chemical elements were quantified in the garlic samples using ICP‐MS. Differences in composition and elements were established among garlic varieties.

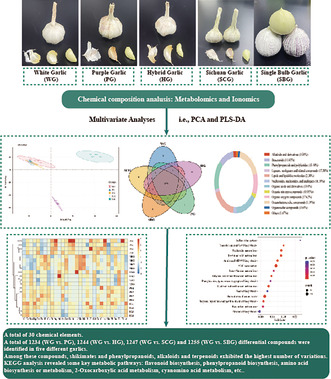

## 1 INTRODUCTION

Due to the COVID‐19 pandemic, consumers' health awareness is evolving. In daily life, people are increasingly paying attention to the consumption of healthy foods. Garlic (*Allium sativum* L.), known for its “medicine food homology” superiority in traditional and alternative medicine, is abundant in China, which is the foremost garlic producer and exporter. The health advantages of garlic can be attributed to its substantial content of minerals, vitamins, essential amino acids, phenols, and notably, organosulfur compounds (OSCs) (Liu et al., 2015). OSCs are the primary chemical compounds that give garlic its distinctive aroma, distinguishing it from other plants, mainly include diallyl sulfide (De Greef et al., 2021), diallyl disulfide (DADS), diallyl trisulfide (DATS), and other allyl polysulfides (Calvo‐Gómez et al., 2004; Chan et al., 2013; Chuah et al., 2007). The sulfonated compound allicin, which is produced from the crushing of the cloves, possess cardiovascular and cerebrovascular protection, anticancer, and antimicrobial effects (Deng et al., 2023). Black garlic is obtained from fresh garlic through a controlled fermentation process involving high humidity and temperature. This fermentation process induces significant changes in its physicochemical properties. Black garlic has been shown to effectively reduce risks associated with diabetes, hypercholesterolemic atherosclerosis, hyperlipidemic hypertension, inflammation, oxidative stress, cancer, and various neurological disorders. The market for black garlic is rapidly expanding due to its beneficial effects on human health. The development of black garlic has further expanded the application of garlic and amplified its benefits (Afzaal et al., 2021).

Garlic possesses saponins, flavonoids, and polysaccharides, each offering distinct biological advantages. Saponins are known for their antioxidant effects, flavonoids have antioxidant and anti‐inflammatory effects, and polysaccharides contribute to overall health by supporting immune function (Shang et al., 2019). The abundance of metabolites and nutrients not only gives garlic its distinctive aroma but also generates a wide range of pharmacological activities, like antibacterial, antioxidant, anti‐inflammatory, immune regulatory, and anticancer effects (Qin et al., 2020; Rana et al., 2011; Zeng et al., 2017). However, there are various varieties of garlic planted in China, the chemical composition of different garlic varieties may vary, thereby offering diverse health benefits. For instance, the quantity of saponins present in purple garlic is nearly 40 times greater than that in white garlic, and certain saponin compounds are exclusively found in purple garlic (Diretto et al., 2017). Garlic possesses a remarkable nutritional profile, encompassing over 20 essential nutrients, including protein, niacin, fat, magnesium, phosphorus, iron, and potassium, among others, which are vital for human bodily functions (Agarwal, 1996). Experts have recognized garlic as an exceptional natural antibiotic and health‐promoting food. The effective components of garlic have been attracting increasing attention because of their significant medicinal value and health function. Currently, certain investigations primarily concentrate on the sulfides present in garlic, specifically alliin and allicin (Ozma et al., 2022). Except for individual sulfur compounds, limited research has been conducted on the comprehensive chemical profiles, synthetic pathways of metabolites, and the composition and content of nutrient elements in various varieties of fresh garlic bulbs. Evaluating the quality of garlic solely based on sulfides lacks comprehensiveness.

This study aims to utilize metabolomics and ionomics technology to investigate the chemical components of garlic and analyze the distinctive constituents of four different garlic varieties cultivated in Jinxiang County, Shandong Province, which is renowned for its Jinxiang garlic, a nationally recognized geographical indication product of China. Then, postauthentication of constituents, advanced statistical methodologies, such as principal component analysis (PCA), orthogonal partial least squares‐discriminant analysis (OPLS‐DA), and hierarchical cluster analysis (HCA), were implemented to effectively cluster and categorize the detected substances. The clustering analysis revealed a notable distinction between five garlic varieties in metabolites. Thereby, this research provides valuable insights into its flavor profile, nutritional composition, and potential therapeutic advantages.

## 2 MATERIALS AND METHODS

### 2.1 Samples, chemicals, and reagents

Fresh garlic, including purple garlic (PG), white garlic (WG), hybrid garlic (HG), Sichuan garlic (SCG), and single bulb garlic (SBG), were the five main varieties cultivated in Jinxiang County. The garlic samples were collected from May to June 2023 at the planting site in Jinxiang County, Jining City, Shandong Province. The samples at the same collection site were uniform in size and free from damage or infection in appearance. The samples were put into foam boxes and ice bags, and immediately transported back to the laboratory and stored in cold storage (4–8°C). Methanol and acetonitrile were purchased from CNW Technologies. Ammonium acetate was purchased from Sigma‐Aldrich. Ethanoic acid and ethanol were purchased from Fisher Chemical. ddH_2_O was purchased from Watsons.

### 2.2 Sample preparation

The garlic bulbs were separated into cloves (50 g), crushed into mashed garlic, and then put in 200 mL 95% (v/v) ethanol ultrasound for 3 h. After drying on a rotary evaporator, a 100 mg/mL solution with 50% (v/v) methanol was prepared. The obtained extracts were filtered through a 0.20‐μm filter and stored at −20°C until analysis.

### 2.3 Metabolites extraction

200 μL of sample was transferred to an EP tube and nitrogen dried. After the addition of 200 μL of extract solution (methanol: water = 3:1, containing isotopically labeled internal standard mixture), the sample was vortexed for 30 s, and sonicated for 15 min in an ice‐water bath. Then, the sample was centrifuged at 12000 rpm (RCF = 13,800 (×g), *R* = 8.6 cm) for 15 min at 4°C. The supernatant was carefully filtered through a 0.22‐μm microporous membrane and transferred to a fresh glass vial for analysis. Quality control (QC) samples were prepared by mixing an equal amount of supernatant from all samples.

### 2.4 LC–MS/MS analysis

LC–MS/MS analyses were performed using an UHPLC system (Vanquish, Thermo Fisher Scientific) with a UPLC HSS T3 column (2.1 × 100 mm, 1.8 μm) coupled to Orbitrap Exploris 120 mass spectrometer (Orbitrap MS, Thermo). The mobile phase consisted of 5 mmol/L ammonium acetate and 5 mmol/L acetic acid in water (A) and acetonitrile (B). The autosampler temperature was 4°C, and the injection volume was 2 μL. The Orbitrap Exploris 120 mass spectrometer was used for its ability to acquire MS/MS spectra on information‐dependent acquisition (Mohd Ariff et al., 2023) mode in the control of the acquisition software (Xcalibur, Thermo). In this mode, the acquisition software continuously evaluates the full scan MS spectrum. The ESI source conditions were set as following: sheath gas flow rate as 50 Arb, Aux gas flow rate as 15 Arb, capillary temperature 320°C, full MS resolution as 60,000, MS/MS resolution as 15,000 collision energy as 10/30/60 in NCE mode, spray Voltage as 3.8 kV (positive) or −3.4 kV (negative), respectively.

### 2.5 Data preprocessing and annotation

ProteoWizard was used to convert raw data into mzXML format and was processed using an internal program developed in R based on XCMS for peak detection, extraction, comparison, and integration. Then, the internal MS2 database (BiotreeDB) was applied to metabolite annotation. The cutoff value for comments was set to 0.3.

### 2.6 ICP‐MS analysis

For the plant, the five fresh garlic varieties were peeled, chopped, and ground. Then, 0.5 g of the uniformly ground tissue was put into the 50‐mL tube, with 7 mL 65% (v/v) nitric acid and 1 mL 15% (v/v) H_2_O_2_, heated in the oven at a temperature of 130°C for 2 h. After cooling to room temperature, dilute with deionized water to the marked area. Stored in 4°C until ready for use. The measurement of element concentrations in the prepared solutions was performed using the ICP‐MS system (Perkin Elmer ELAN 9000, Perkin Elmer Sciex Penlivia, Canada). The ICP‐MS conditions were: RF power 1300 W; plasma gas flow rate: 16 L/min; auxiliary gas flow rate: 1.25 L/min; gas flow of spray: 0.9 L/min; spray, cross flow; spray room, PFA dual gas; and monitor ion m/z. Quantification was measured using an external calibration curve prepared by high‐purity ICP multi‐element calibration standard (Tan‐Mo Technology Co., Ltd.). The data were processed and explained in units of mg/kg for the garlic samples. Each sample was made in triplicate, and the average value with a relative standard deviation below 10% is representative for further analysis.

### 2.7 Statistical analysis

Univariate statistical analyses were conducted using Student's *t*‐test. Multivariate statistical analysis was performed using principal component analysis (PCA) and orthogonal partial least squares discriminant analysis (OPLS‐DA). Additionally, other differential compounds were screened and identified. Subsequently, hierarchical clustering analysis, correlation analysis of differential metabolites, KEGG (Kyoto Encyclopedia of Genes and Genomes) annotation, differential metabolite pathway analysis, and regulatory network analysis of differential metabolites were carried out following the identification of differential metabolites.

## 3 RESULTS AND DISCUSSION

### 3.1 Appearance attributes of five garlic samples

**FIGURE 1:**
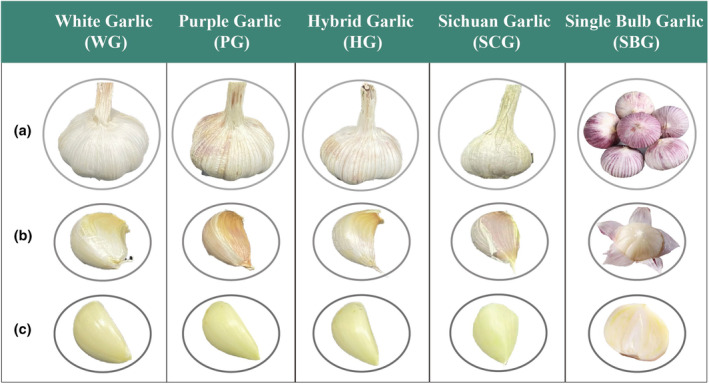
Appearance profiles of five garlics. (a) The appearance of the five garlic varieties. (b) The morphology of the cloves of the five garlic varieties after removing the outer skin. (c) The morphology of the cloves after removing the skin.

Figure 1 displays five fresh garlic samples: WG, PG, HG, SCG, and SBG. With the exception of WG, the skins of the other four types of garlic are all purple. WG, PG, and HG have similar shapes and sizes, but WG is white, while PG and HG have varying shades of purple. The bulbs of SCG are significantly smaller and harder compared to those of WG, PG, and HG. SCG is similar in size to SBG, but unlike SBG, which consists of a single clove forming a complete bulb.

In living organisms, the synthesis of specific amino acids and proteins is essential for sustaining normal physiological functions. These substances, commonly known as primary metabolites, encompass various biomolecules, including nucleotides and fatty acids. Secondary metabolites are the consequence of plants' enduring adaptation to their indigenous surroundings, serving to enhance their self‐defense, viability, competitive advantage, coordination, and interaction with the surrounding ecosystem (Rizhsky et al., 2004). A secondary metabolite exhibits a higher correlation and a greater ability to adapt to its environment compared with its primary counterpart.

In recent years, consumer demand for health products and organic products has been increasing. However, factors such as low yields, lack of information, lack of organic markets, lack of government support, and limited supply of organic fertilizers are obstacles faced by organic agriculture. The implementation of organic farming is hindered by a lack of knowledge, better varieties, extension services, decision‐making, and access opportunities (Saeed et al., 2023). This study analysis of the metabolites of different garlic varieties helps to understand the nutritional differences between different varieties, and other differences between different varieties, which relate to the cultivation of garlic.

### 3.2 Secondary metabolic profiling of five garlic varieties

**FIGURE 2:**
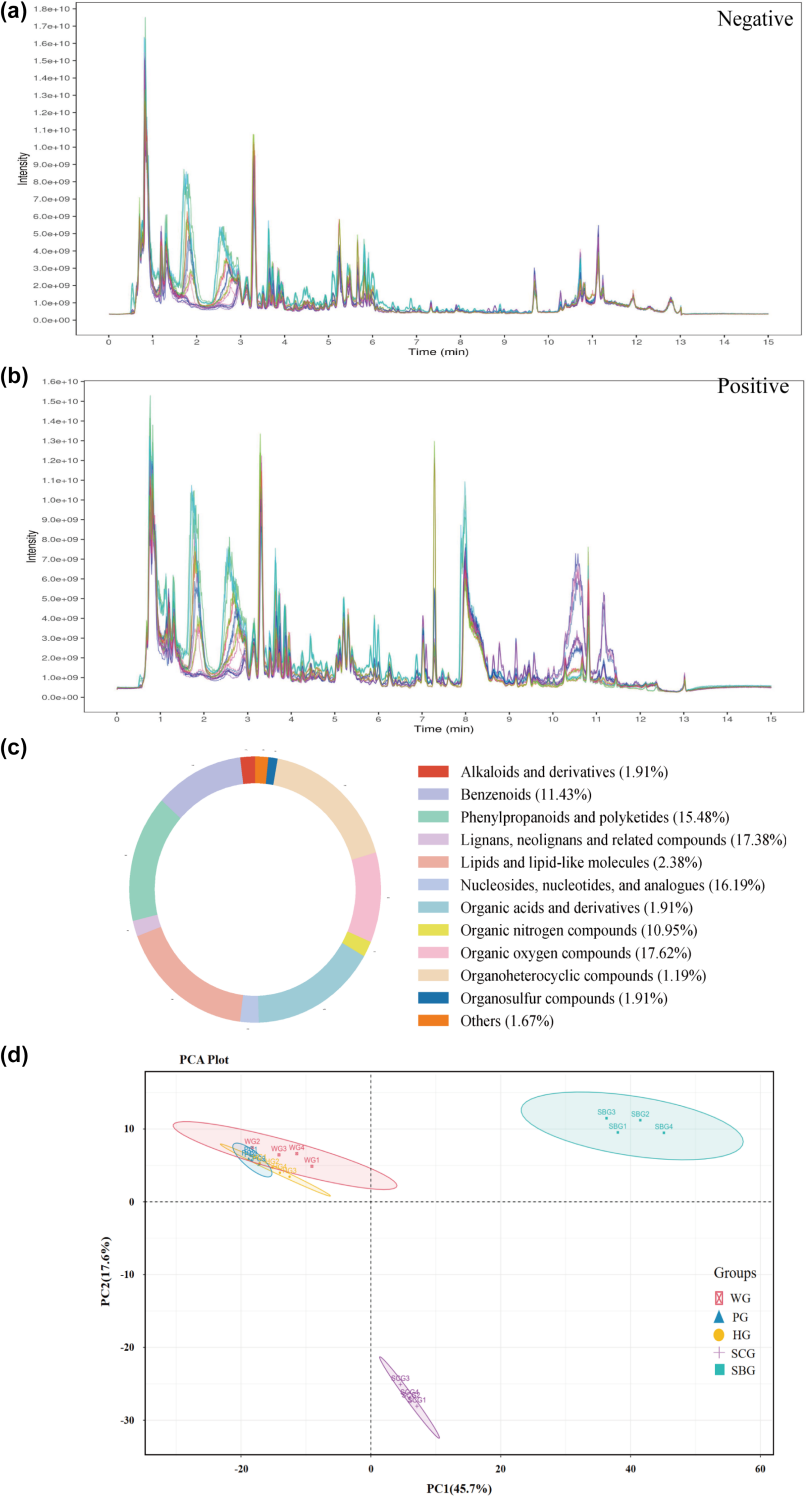
Secondary metabolic profiling of five garlic varieties. Total ion current of five garlic varieties obtained by mass spectrometry detection. (a) A multi‐peak detection plot of the negative mode. (b) A multi‐peak detection plot of the positive mode. (c) Classification of the metabolites of garlic samples. (d) Scatter plot of principal component analysis (PCA).

In this study, no statistically significant variations were observed in plant growth parameters, encompassing planting environment, nutritional factors, and environmental factors. The LC–MS/MS total ion current (TIC) chromatograms of the garlic under positive and negative electrospray ionization modes are depicted in Figure 2a,b, respectively. The peak heights of specific components exhibited varied across the five garlic varieties, suggesting disparities in the expression and content of metabolites. To assess the diversity of their metabolites, LC–MS/MS metabolic profiling analyses were conducted on five varieties of garlic, providing comprehensive insights into their metabolic distinctions.

Metabolite data indicated that a total of 1256 metabolites were identified in the five garlic groups through untargeted metabolomics. The identified metabolites were lipids and lipid‐like molecules (17.38%), organic acids (16.19%), phenylpropanoids and polyketides (15.48%), organoheterocyclic compounds (17.62%), benzenoids (11.43%), organic oxygen compounds (10.95%), lignans, neolignans and related compounds (1.90%), nucleosides, nucleotides and analogs (2.38%), alkaloids and derivatives (1.90%), and other organic compounds like organosulfur compounds (1.19%) and organic nitrogen compounds (1.90%) (Figure 2c). Our research on garlic metabolomics data provides a comprehensive exploration of the biochemical composition of garlic, shedding light on the intricate network of metabolites that contribute to its unique properties. The metabolomics data in the study reveal the rich diversity of compounds present in garlic, which not only contribute to its unique taste and aroma but also impact its potential medicinal and nutritional value.

### 3.3 Multivariate statistical analysis

Principal component analysis (PCA) was used to illustrate the variability in the composition of the extracts obtained from the five garlic varieties. The dense clustering of QC samples on the PCA score plot confirmed the stability and reliability of the chromatography and quality detection system. The PCA plot showed that the first and second principal components accounted for 45.7% and 17.6% of the total variance within the dataset, respectively. As shown in Figure 2d, a discernible differentiation among the five samples was observed: WG, PG, and HG showed an aggregation trend in PCA and were significantly separated from SBG on PC1 and SCG on PC2. Additionally, the tight grouping of the four biological replicates of each variety underscored the good reproducibility within each group. Preliminary results indicate that although there are differences in the varieties of WG, PG, and HG, there is a high degree of similarity in their metabolites. In contrast, the metabolites of SCG and SBG differ significantly from those of the other garlic varieties.

**FIGURE 3:**
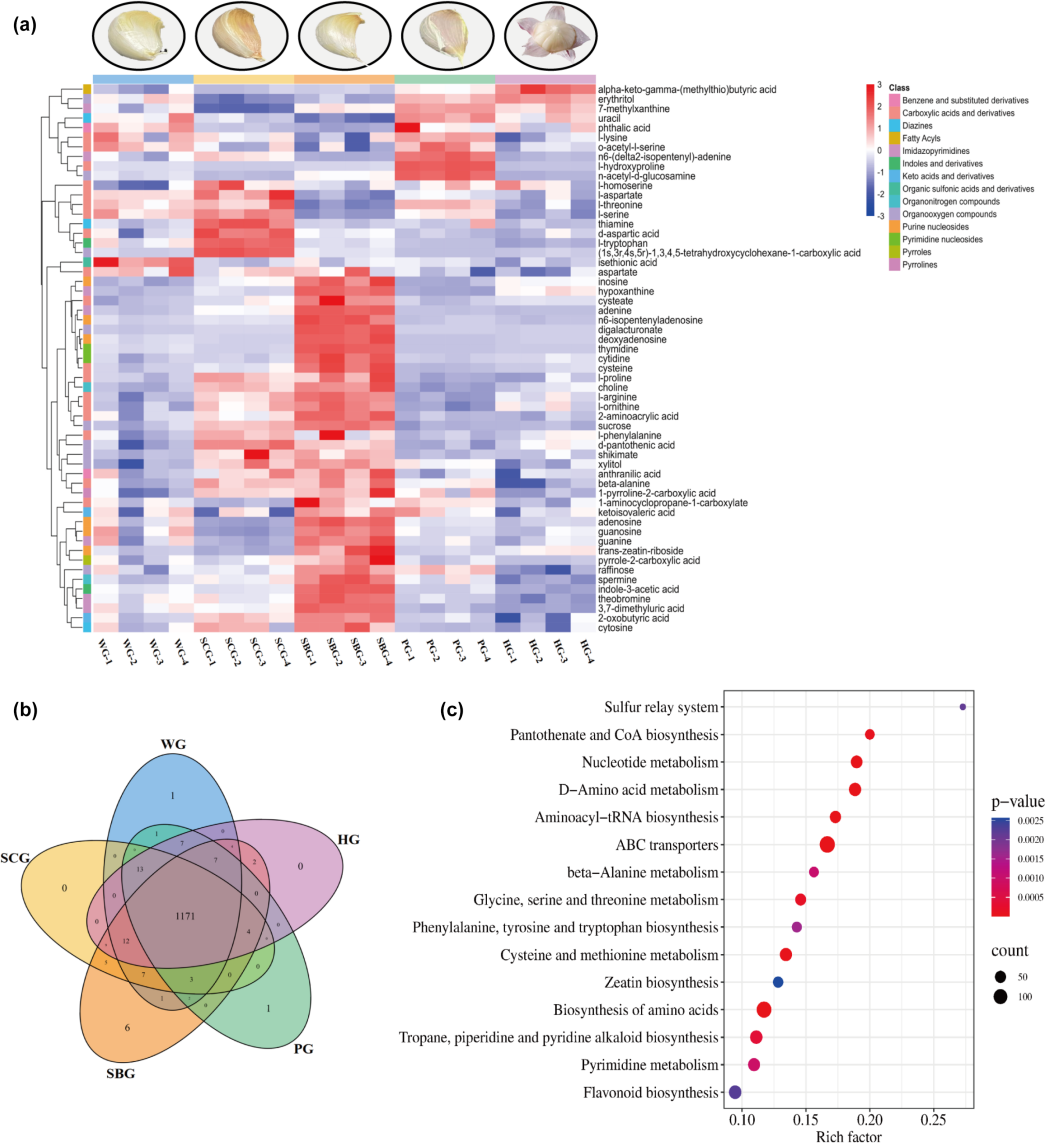
Proportions and clustering heatmaps of differential compounds. (a) Metabolite classification and proportion; (b) thermogram analysis of differential metabolite content; and (c) Air Bubble Diagram of differential metabolite KEGG enrichment.

Heatmaps depicting the clustering of the five garlic varieties were generated using metabolomics data. Hierarchical clustering analysis (HCA) showed significant differences in the levels of differential metabolites among the five garlic groups (Figure 3a). Although WG, HG, and PG share similarities in appearance, shape, and other features, their metabolite expression exhibits certain differences. The expression trends of metabolites in SCG and SBG are different from those of the other three types of garlic, and the metabolite accumulation pattern of SBG markedly diverged from the others. A comparative analysis of the total metabolites of the five garlic varieties was conducted using Venn plots to represent the results. It was found that among all 1256 metabolites, WG and PG had one unique metabolite, while SBG had six unique metabolites (Figure 3b). Significant differences in metabolites were observed among the garlic varieties, each exhibiting distinct metabolic profiles.

### 3.4 KEGG enrichment analysis of the differential metabolites

By comparing with the KEGG database, genes can be categorized according to their participation in pathways or functions, providing information on the metabolic pathways involving the metabolites. The identified differential metabolites from the screening were then matched with relevant information, followed by correlation analysis of the metabolites and pathways exhibiting notable disparities. KEGG analysis revealed that 37 pathways exhibited significant differences (*p* < .05), the top 15 pathways including d‐Amino acid metabolism, cysteine and methionine metabolism, glycine, serine and threonine metabolism, phenylalanine, tyrosine and tryptophan biosynthesis, and flavonoid biosynthesis (Figure 3c). These pathways are closely related to the adaptability of garlic to its growth environment, color formation, and synthesis of characteristic substances and nutrients.

By utilizing the KEGG database to map differential metabolites, it was observed that a total of 14 substances exhibited highly significant differences. These substances included l‐threonine, l‐hydroxyproline, l‐phenylalanine, l‐arginine, l‐serine, cysteine, l‐proline, l‐lysine, l‐aspartate, l‐ornithine, o‐acetyl‐l‐serine, d‐aspartic acid, 2‐aminoacrylic acid, and cysteate. Among these metabolites, l‐threonine, l‐phenylalanine, and l‐tryptophan are involved in three metabolic pathways concurrently, while l‐serine, cysteine, and l‐aspartate participate in four metabolic pathways simultaneously. It is known that the composition of food can vary according to its origin. The abundance and composition of amino acids in garlic can provide valuable information about its quality and nutritional value. Interestingly, the content of amino acids varied among garlic samples from different geographical origins (Pacholczyk‐Sienicka et al., 2024). We found that the expression of these 14 amino acids varied among the five garlic varieties. The content of 1‐hydroxyproline was highest in PG, being 5–11 times higher than in the other garlic varieties. In SBG, 4 amino acids (l‐arginine, 2‐aminoacrylic acid, cysteine, and l‐ornithine) were present in higher concentrations compared to the other varieties, whereas in PG, the levels of these amino acids were extremely low. Additionally, iaspartate was significantly expressed in SCG (Table S1). The differential metabolites are primarily influenced by the metabolic utilization of garlic compounds, with the different types and contents of metabolites contributing to garlic's unique characteristics. Our research demonstrates that garlic contains a wide variety of primary metabolites, including nucleotides and amino acids, including L‐tryptophan, L‐arginine, L‐threonine, L‐serine, and so on. Simultaneously, organisms undergo alterations in specific metabolic pathways and generate distinct metabolites in response to abiotic stresses, plant hormones, and other external factors as a means of adapting to environmental stressors and defending against external adversaries (Zhang et al., 2015).

### 3.5 Tentative identification of compounds present between WG and other garlics

To further understand the differences in metabolites, the five varieties were compared pairwise. The OPLS‐DA plots (Figure S1) showed notable differences in metabolic phenotypes between the four groups (WG vs. PG, WG vs. HG, WG vs. SCG, WG vs. SBG). WG is a variety selected by the Agricultural Bureau of Jinxiang County, Shandong Province, known for its disease resistance, high quality, and high yield. This variety was successfully selected in 1996 and has been expanded in recent years (Li et al., 2022; Toledano Medina et al., 2019). Currently, it possesses the most extensive cultivation region and serves as a substitute cultivar for exporting and generating foreign currency in garlic production regions. Consequently, the polar extracts of WG were compared to those of four other garlic varieties in terms of their metabolites. To ensure quality control and assurance during the LC–MS detection process, each sample underwent four assays.

**FIGURE 4:**
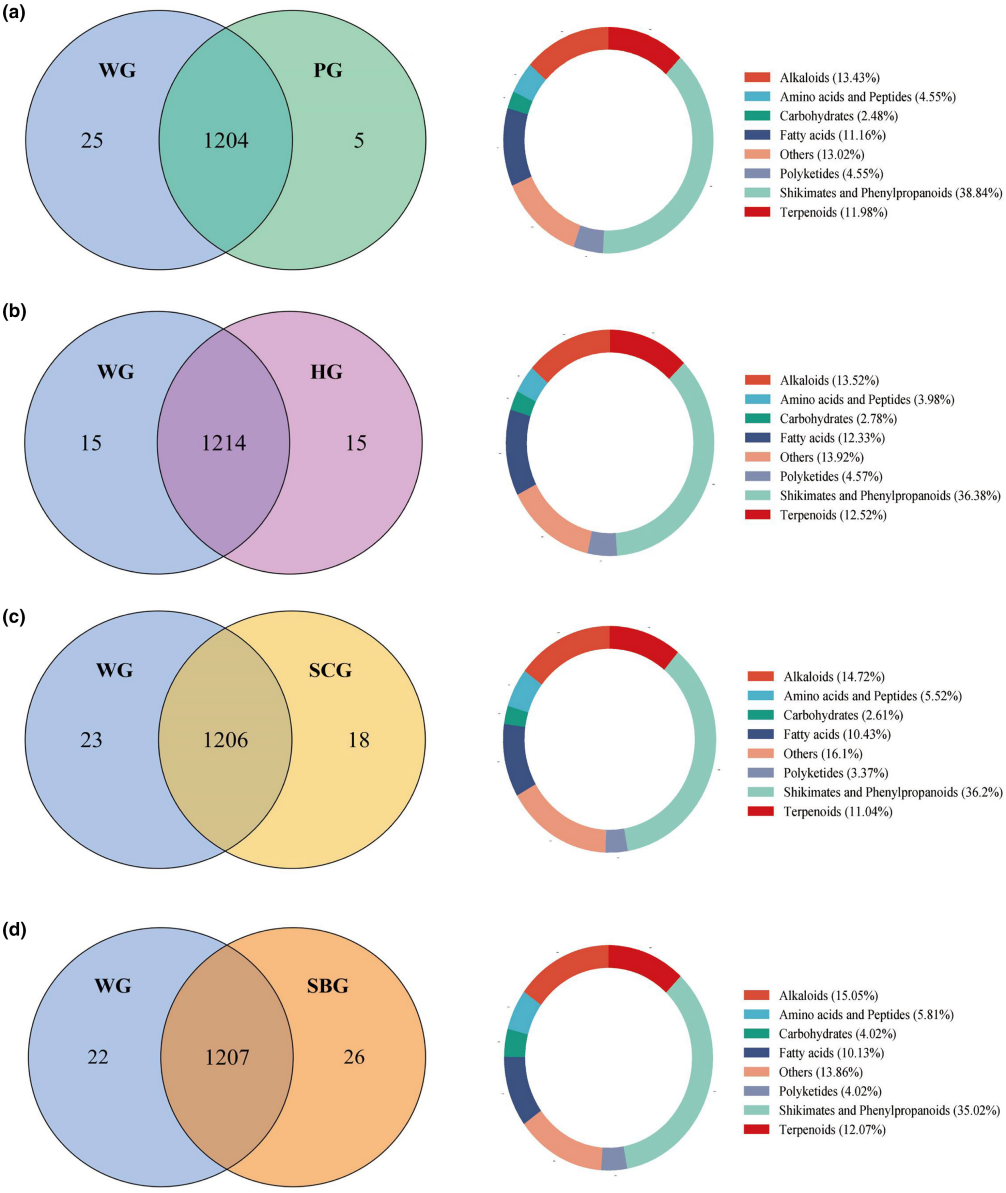
Venn diagram and the superclass of identified metabolites. (a) WG versus PG; (b) WG versus HG; (c) WG versus SCG; and (d) WG versus SBG.

To investigate the presence of common and unique metabolites among different groups, Venn diagrams were utilized for comparative analysis. The Venn diagram analysis revealed that a total of 1234 compounds were detected in both the WG versus PG group, with 25 compounds exclusively detected in the WG group and 5 compounds exclusively detected in the PG group. Similarly, in the WG versus HG group, a total of 1244 compounds were detected, with 15 compounds exclusively detected in the WG group and 15 compounds exclusively detected in the HG group. Furthermore, in the WG versus SCG group, a total of 1247 compounds were detected, with 23 compounds exclusively detected in the WG group and 18 compounds exclusively detected in the SCG group. A total of 1255 compounds were detected in both the WG versus SBG group, with 22 compounds exclusively detected in the WG group and 26 compounds exclusively detected in the SBG group (Figure 4). From the proportion chart of differential metabolite categories, it can be seen that the types of differential metabolites in each group mainly include shikimates and phenylpropanoids, alkaloids, terpenoids, fatty acids, and others. These differential metabolites shikimates and phenylpropanoids including flavonoids, lignans, phenolic acid and coumarins (38.84% of WG vs. PG group, 36.38% of WG vs. HG group, 36.2% of WG vs. SCG group, 35.02% of WG vs. SBG group), terpenoids (11.98% of WG vs. PG group, 12.52% of WG vs. HG group, 11.04% of WG vs. SCG group, 12.07% of WG vs. SBG group), alkaloids (13.43% of WG vs. PG group, 13.52% of WG vs. HG group, 14.72% of WG vs. SCG group, 15.05% of WG vs. SBG group), fatty acids (11.16% of WG vs. PG group, 12.33% of WG vs. HG group, 10.43% of WG vs. SCG group, 10.13% of WG vs. SBG group). Significantly, shikimates and phenylpropanoids, alkaloids, and terpenoids displayed the highest number of discrepancies. These three compound categories constituted a substantial proportion of the identified variances, ranging from 35.02% to 38.84% for shikimates and phenylpropanoids, 13.43%–15.05% for alkaloids, and 11.04%–12.52% for terpenoids, surpassing the other compound groups. These results indicated that the different garlics contained a wide variety of metabolites, including multiple common and unique metabolites of each type of garlic.

**FIGURE 5:**
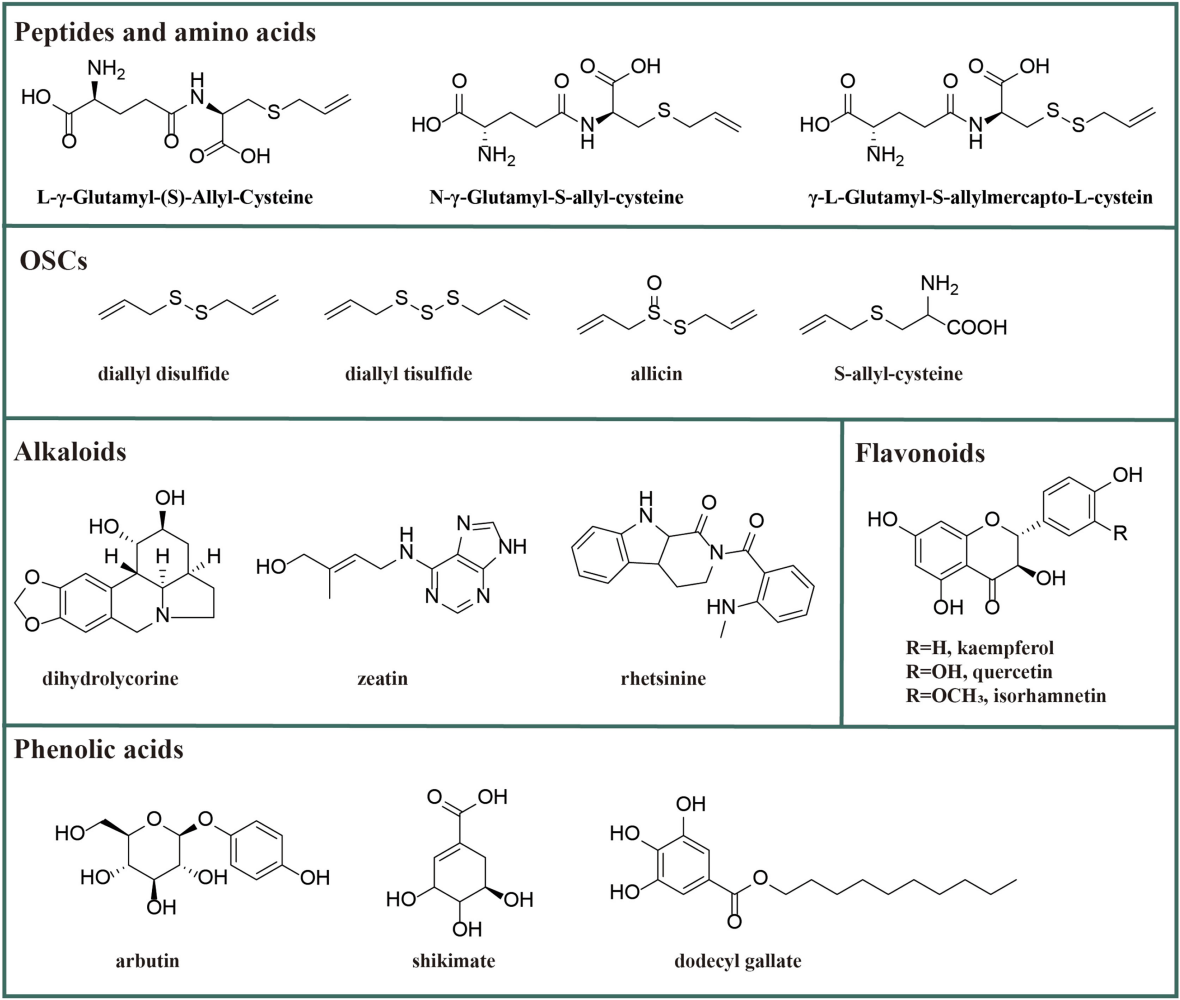
Examples of natural product classes reported in garlic.

The metabolites of four distinct varieties of garlic were compared to those of WG, the results emphasized the diversity and richness of metabolites in five varieties, revealing their potential health‐promoting and flavor characteristics. The differential metabolites in each group can be categorized into the following classes: shikimates and phenylpropanoids, alkaloids, terpenoids, fatty acids, and others (Figure 5). These metabolites play a crucial role in garlic's anti‐inflammatory and antioxidant effects, as well as in the treatment of diseases (Gao et al., 2021; Qiu et al., 2020). Shikimates and phenylpropanoids include flavonoids, lignans, phenolic acids, phenylpropanoids, and coumarins. Flavonoids are synthetized via the shikimic acid pathway (Santos‐Buelga & Feliciano, 2017). The majority of phenolic compounds in garlic are organic compounds with acidic properties, such as β‐phthalic acid, containing functional groups like ‐CHO, which mainly exert antioxidant properties (Mohd Ariff et al., 2023; Skoczylas et al., 2023). Plants with high polyphenol content have the ability to slow down the oxidative breakdown of lipids, enhancing the quality and nutritional content of food (Barajas‐Ramírez et al., 2023). Garlic and onion, especially, are recognized as among the most abundant sources of phenolic compounds, specifically flavonoids. Photodiode array inspection of peaks provided a comprehensive understanding of their primary flavonoid subclasses (Kova et al., 2023). The flavonoids in garlic mainly include kaempferol, quercetin, and isorhamnetin, as well as O‐ or C‐glycosides. In addition to maintaining the normal growth of garlic itself, pure flavonoids have been shown to have antioxidant and anti‐inflammatory effects (Dabeek & Marra, 2019).

In the WG versus PG group, the most abundant differential metabolites are flavonoids, which predominantly show an upregulation trend in WG. A similar trend is observed in the WG versus HG group. The flavonoids in garlic mainly include kaempferol, quercetin, and isorhamnetin, as well as O‐ or C‐glycosides. These flavonoids not only support the normal growth of garlic but also exhibit antioxidant and anti‐inflammatory effects (Santos‐Buelga & Feliciano, 2017). This suggests that among the three garlic varieties with high similarity, WG may exhibit better antioxidant and anti‐inflammatory effects compared to HG and PG. Additionally, the number of differential metabolites in WG is substantially higher than in the PG and HG groups. Metabolites such as phloretin, chlorogenic acid, luteolin, stearidonic acid, etc., which primarily contribute to scavenging free radicals, exerting anti‐inflammatory and antibacterial effects (Anunciato Casarini et al., 2020; Naveed et al., 2018), are more abundant in WG. Moreover, there is a significant increase in the number of upregulated alkaloids, lignans, coumarins, terpenoids, and phenolic acids metabolites in WG compared to the downregulated ones. The results of WG versus SCG and WG versus SBG groups are markedly different from the first two groups, indicating significant differences between SCG and SBG compared to the other three garlic varieties (Figures S2–S5).

### 3.6 Key differential metabolites in different garlic varieties

**FIGURE 6:**
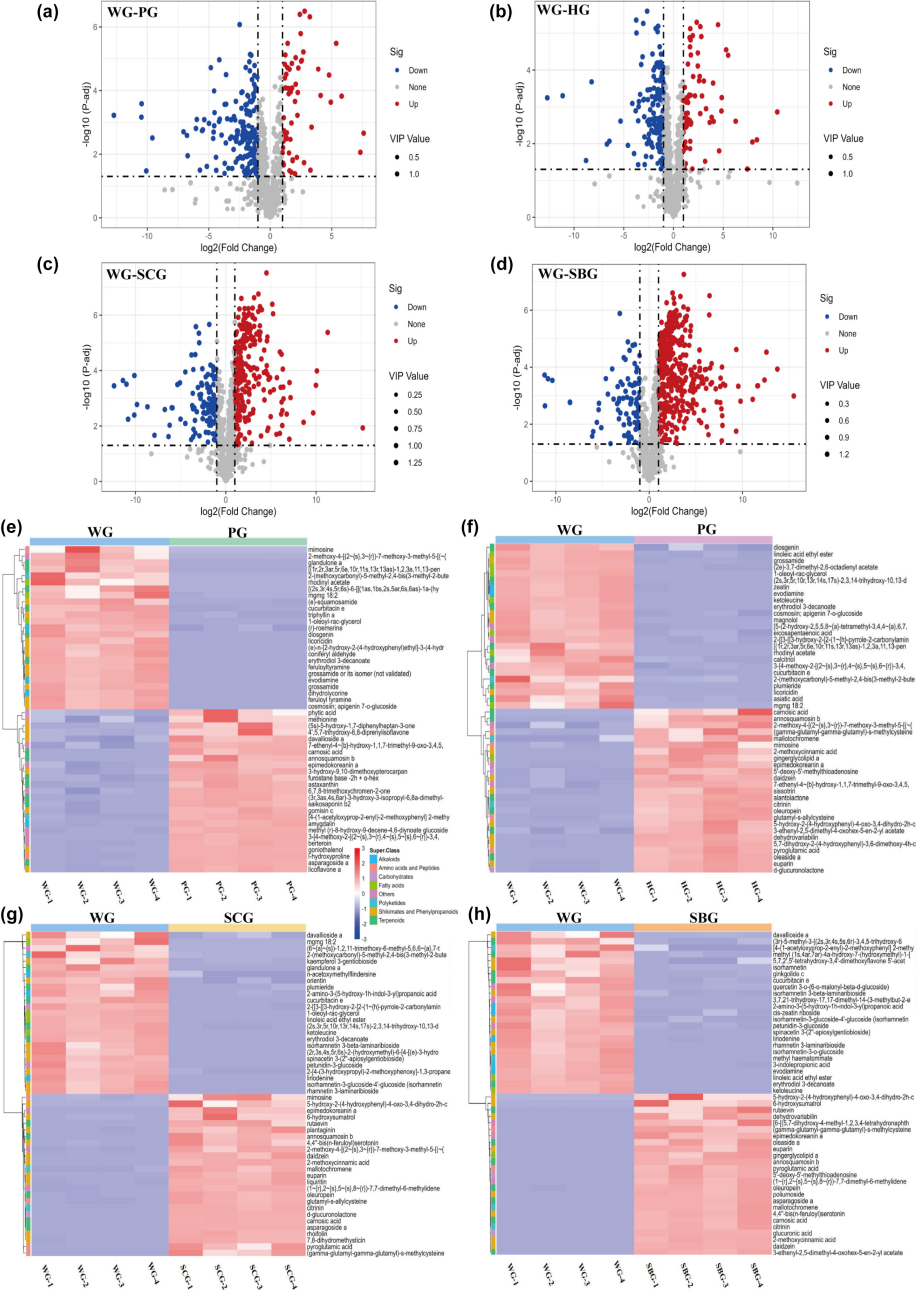
Identification and classification of metabolites between WG and other garlics. The volcano maps exhibited the differential metabolites between four groups under positive and negative ion modes. (a) WG versus PG; (b) WG versus HG; (c) WG versus SCG; (d) WG versus SBG and heatmap of metabolites; (e) WG versus PG; (f) WG versus HG; (g) WG versus SCG; and (h) WG versus SBG.

To identify the compounds responsible for the differences between WG versus PG, WG versus HG, WG versus SCG, and WG versus SBG, the metabolites were considered significantly different while VIP > 1, *p* < .05, and fold change (FC) ≥2 or ≤0.5. These metabolites were categorized into various groups, including acids, phenols, phenylpropanoic acids, sugars and glycosides, flavonoids, quinones, steroids, terpenoids, alkaloids, and other natural products. A total of 255 significantly different metabolites were identified between the WG versus PG group, with 61 metabolites showing increased abundance and 194 metabolites showing a decrease in the PG group compared with WG (Figure 6a). Analysis revealed 231 metabolites with differential abundances between WG and HG, where 77 metabolites were significantly upregulated and 154 metabolites were significantly downregulated in WG (Figure 6b). Between WG and SCG, 397 metabolites exhibited significant differences, with 276 metabolites significantly upregulated and 121 metabolites significantly downregulated in WG (Figure 6c). Lastly, 495 metabolites showed significant differences between WG and SBG, with 415 metabolites upregulated and 80 metabolites downregulated in WG compared to SBG (Figure 6d).

In the WG group, several metabolites showed significant increases, including methionine, a sulfur‐containing essential amino acid that humans cannot synthesize and must obtain through diet (Amir et al., 2019). Methionine is well‐known for its involvement in various aspects of plant growth (Escaray et al., 2022) and serves as a crucial precursor for garlic's diverse pharmacological activities. Additionally, it can be utilized in the synthesis of S‐methyl‐L‐cysteine and its sulfoxide compounds within garlic. The concentrations of tyrosine alkaloids, specifically allicin and thiamine, also showed notable increases. Furthermore, compared to other garlic varieties, WG was enriched with metabolites such as glutamyl‐S‐methylcysteine, glutamyl‐S‐allylcysteine, and glutamyl‐S‐allylthio‐L‐ cysteine. Organosulfur dipeptides in Allium species are particularly significant, as they mediate medicinal properties and contribute to sensory characteristics. Garlic, in particular, contains a high concentration of γ‐glutamyl peptides and sulfoxides, which are of considerable interest (Hernandez‐Cruz et al., 2022).

HCA was employed to summarize the variation in the levels of the distinct metabolites across the five garlic varieties. The heat map reveals notable disparities in metabolites between each group upon comparison, specifically highlighting the initial 25 and final 25 differential metabolites that exhibit significant differences (|log(FC)| > 1) (Figure 6e–h). HCA showed significant differences in the levels of differential metabolites between the WG group and other garlic groups. The types of differential metabolites mainly involve several categories such as alkaloids, amino acids and peptides, carbohydrates, polyketides, shikimates and phenylpropanoids, terpenoids, and fatty acids.

### 3.7 KEGG pathway analysis of differential metabolites between WG and other garlics

**FIGURE 7:**
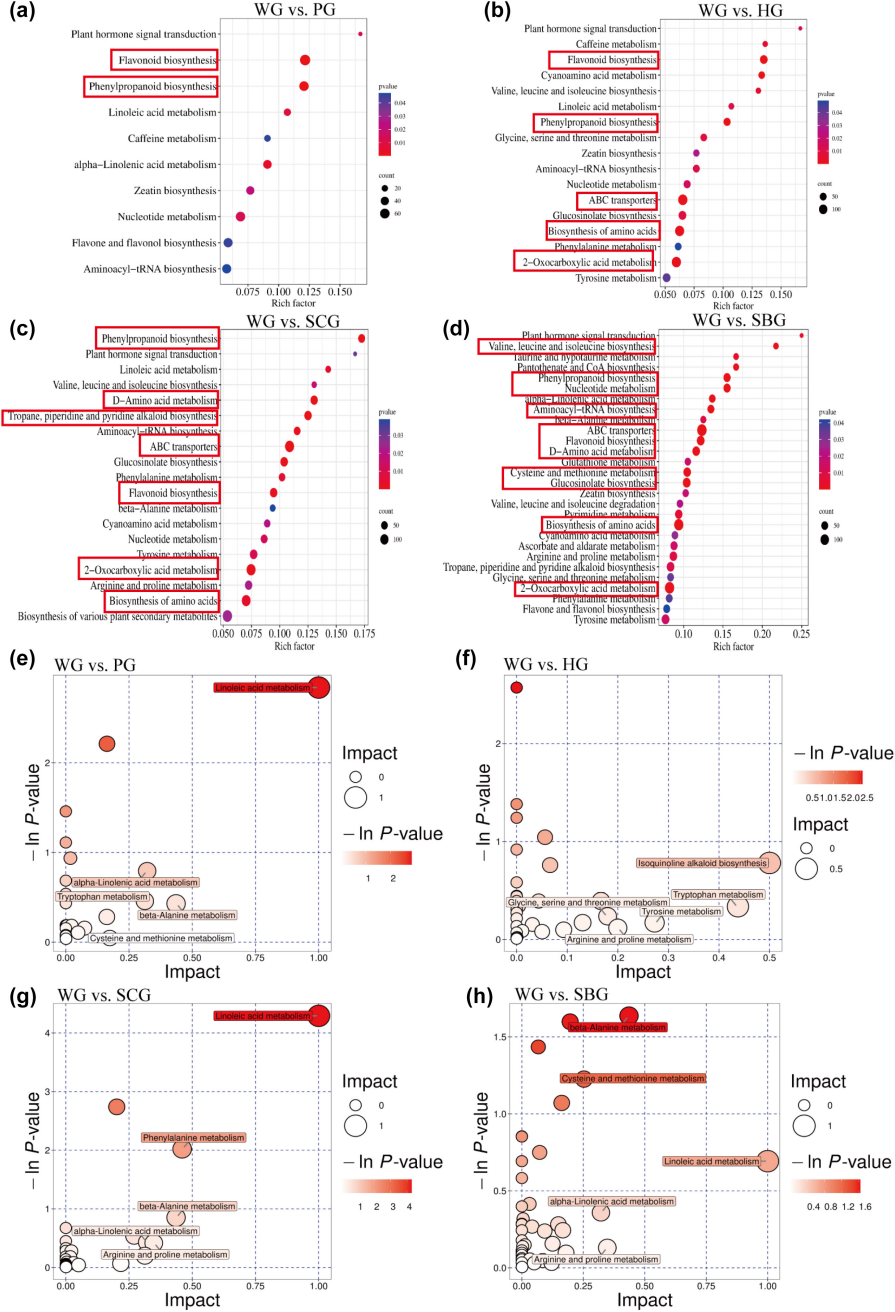
Bubble diagram of differential metabolite KEGG enrichment (a) WG versus PG; (b) WG versus HG; (c) WG versus SCG; (d) WG versus SBG, and analysis of key KEGG pathways (e) WG versus PG; (f) WG versus HG; (g) WG versus SCG; and (h) WG versus SBG.

The metabolic pathways of all the differential metabolites were acquired from the KEGG database. The enrichment analysis of the identified metabolic pathways yielded a total of 54 differential metabolite pathways in the WG versus PG group. Among these, 10 metabolic pathways exhibited significant differences (*p* < .05). Notably enriched pathways included flavonoid biosynthesis, phenylpropanoid biosynthesis, alpha‐linolenic acid metabolism, and linoleic acid metabolism (Figure 7a). In the WG versus HG comparison, 70 differential metabolite pathways were enriched, with 17 pathways exhibiting significant differences (*p* < .05). These pathways included flavonoid biosynthesis, cyanoamino acid metabolism, and phenylpropanoid biosynthesis (Figure 7b). The KEGG pathway analysis results for significantly abundant metabolites (DAMs) in WG versus SCG were illustrated in a bubble diagram (Figure 7c), revealing 19 pathways with significant differences (*p* < .05). For WG versus SBG, DAMs were enriched in 28 metabolic pathways, including ABC transporters, nucleotide metabolism and phenylalanine metabolism (Figure 7d). Further analysis in Figure 7e–h showed that the differential metabolites in four groups were enriched in some key metabolic pathways: linoleic acid metabolism, alpha‐linolenic acid metabolism, tryptophan metabolism, isoquinoline alkaloid biosynthesis, glycine, serine and threonine metabolism, beta‐alanine metabolism, cysteine and methionine metabolism, phenylalanine metabolism. The metabolites involved in the main metabolic pathways include several categories, the compounds are crucial for the characteristics of the garlic and primarily include compounds from categories such as flavonoids, alkaloids, phenolic acids, lignans and coumarins, and terpenoid metabolites.

**FIGURE 8:**
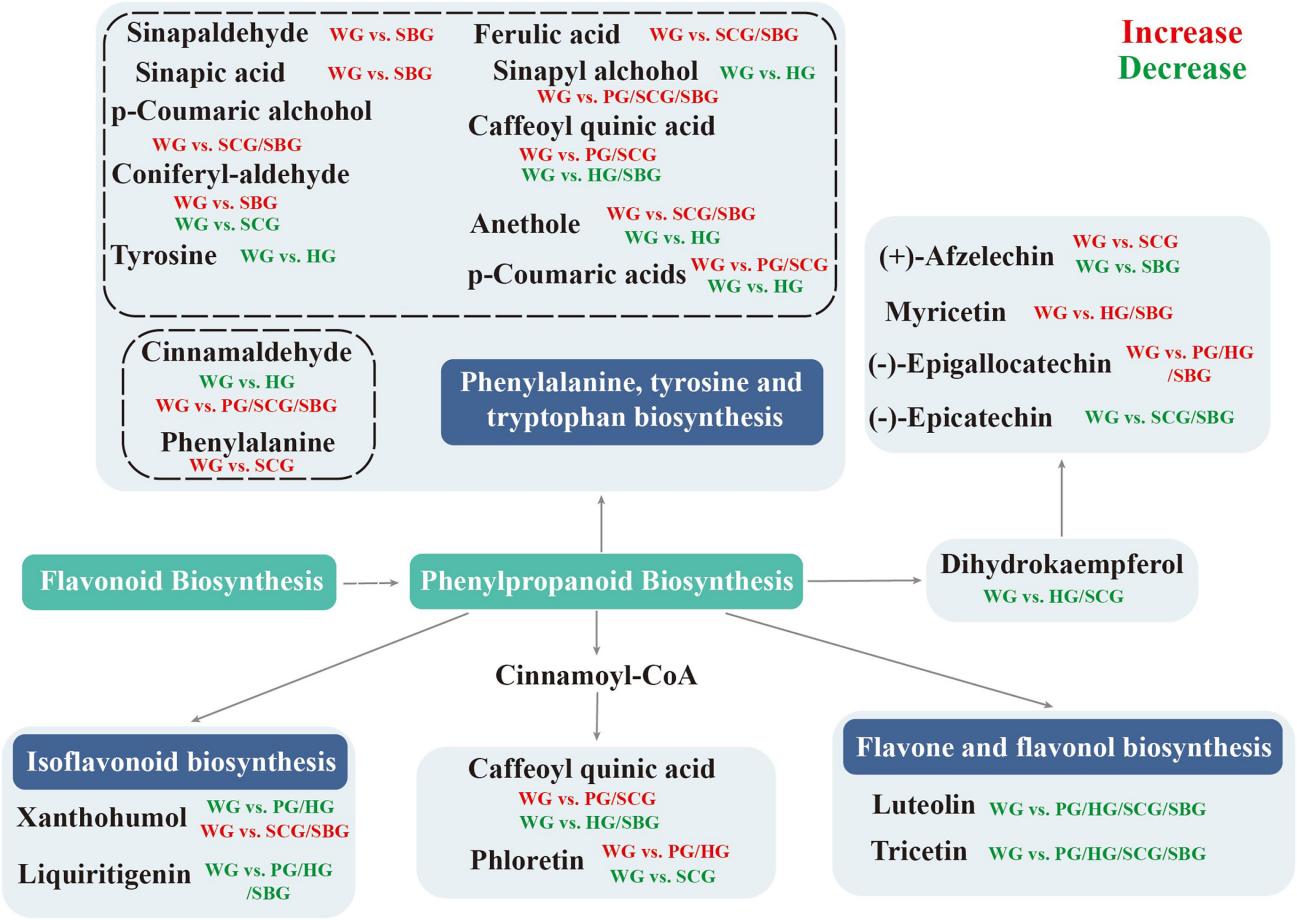
Expression of differential metabolites in flavonoid biosynthesis and phenylpropanoid biosynthesis pathways.

The analysis results of KEGG further validate the above results (Figures S6–S10). In the KEGG analysis of the four groups, pathways related to flavonoid biosynthesis, phenylpropanoid biosynthesis, tropane, piperidine and pyridine alkaloid biosynthesis, and alpha‐linolenic acid metabolism were found to be significantly enriched. These pathways are closely associated with the biosynthesis of flavonoids, phenylpropanoids, and alkaloids. The phenylpropane biosynthetic pathway begins with phenylalanine or tyrosine as the initial substrate. Enzymes such as PAL regulate this pathway, resulting in the production of hydroxyaromatic metabolites like flavonoids, coumarins, and phenolic compounds (Deng & Lu, 2017). In the WG versus PG and WG versus HG groups, the two most significantly enriched pathways are related to flavonoid biosynthesis and phenylpropanoid biosynthesis. Flavonoids constitute a vast and diverse category of secondary metabolites synthesized via a distinct branch of the phenylpropanoid pathway (Li et al., 2017). The expression of 6 metabolites showed increased (sinapyl alcohol, caffeoylquinic acid, p‐coumaric acids, cinnamaldehyde, (−)‐epigallocatechin, phloretin) in WG versus PG group, while the expression of 4 metabolites (xanthohumol, liquiritigenin, luteolin, and tricetin) was decreased in WG versus PG and WG versus HG groups (Figure 8). 5 metabolites were increased both in WG versus SCG group and WG versus SBG group, while the expression of 3 metabolites was downregulated. In the WG versus PG group, pathways significantly enriched in differential metabolites also include alpha‐linolenic acid metabolism and linoleic acid metabolism, apart from their association with garlic flavor, these metabolites also participate in jasmonic acid synthesis and wound healing during plant trauma (Wang et al., 2021). Therefore, based on the KEGG analysis results, WG demonstrates greater potential in terms of flavor, sweetness, spiciness, antioxidant activity, free radical scavenging, antibacterial, and anti‐inflammatory properties, alongside stronger environmental adaptability compared to other types of garlic.

**FIGURE 9:**
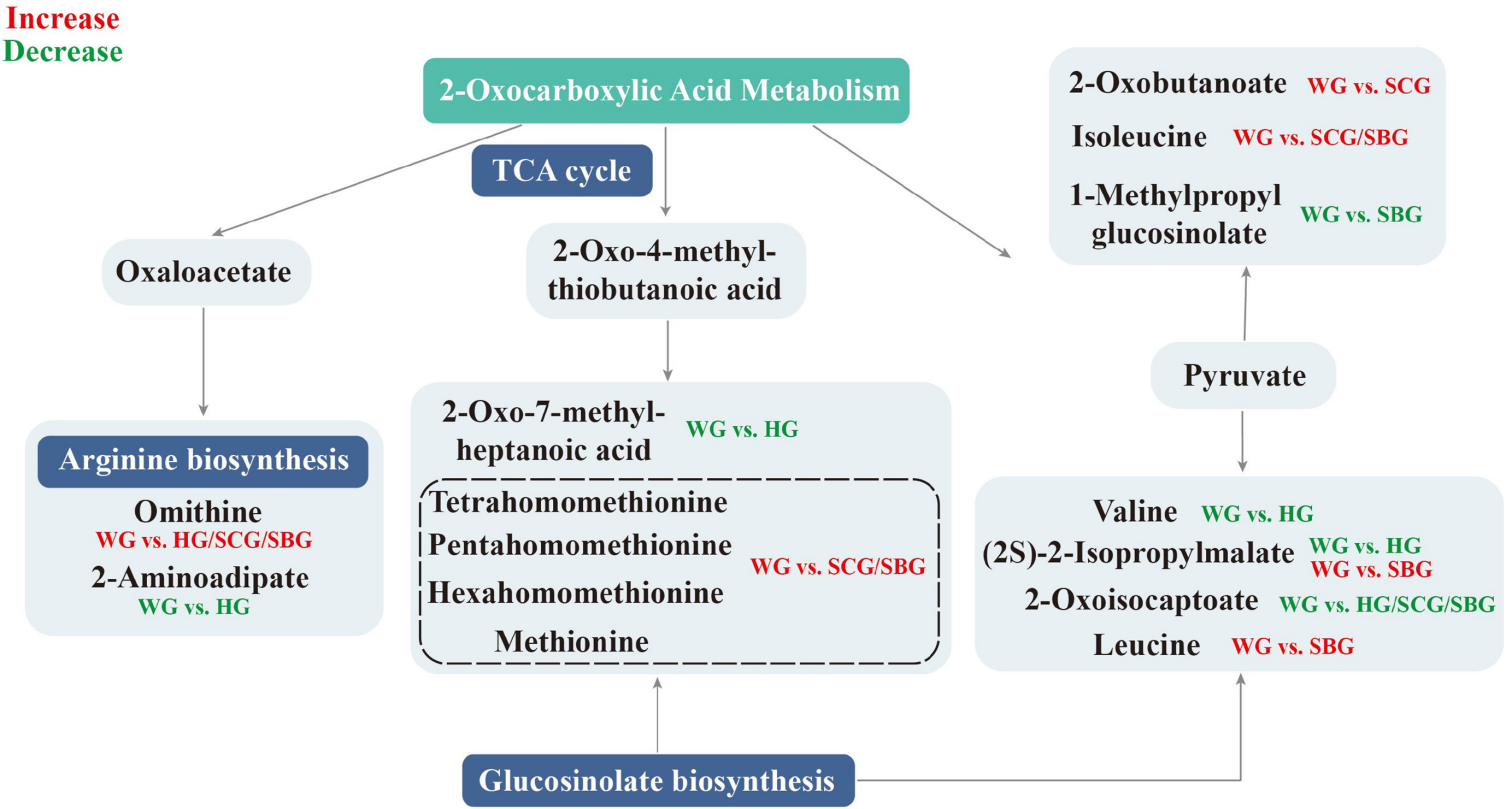
Expression of differential metabolites in 2‐oxocarboxylic acid metabolism pathway.

Another significantly enriched pathway in the WG versus HG, WG versus SCG, and WG versus SBG groups is the 2‐Oxocarboxylic acid metabolism pathway (Figure 9). This pathway is crucial for plant metabolism, involving the processing of various organic acids. 2‐oxocarboxylic acid metabolism is linked to various linked to several key processes, including amino acid biosynthesis, tricarboxylic acid cycle (TCA), and glucosinolate biosynthesis (Munir et al., 2020). It facilitates energy production by converting 2‐oxocarboxylic acids into other compounds, with molecules like methionine and its derivatives, such as dihydromethionine, playing a significant role. These derivatives are involved in the 2‐oxocarboxylic acid metabolism pathway and exhibit notable antioxidant activity, aiding plants in managing environmental stress, including oxidative stress (Saeed et al., 2017). Differently, this pathway was downregulated in the WG versus HG group but upregulated in the WG versus SCG group and WG versus SBG (Figures S6‐1 to S6‐4). Analysis of differential metabolites associated with this pathway revealed that the downregulation observed in the WG versus HG group may be due to the downregulation of the expression of 5 metabolites. Notably, 2‐oxoisocaptoate expression was downregulated across all three groups, and 1‐methylpropyl glucosinolate was downregulated in the WG versus SBG group. However, all other differential metabolites related to this pathway were upregulated in the WG versus SCG, and WG versus SBG groups.

**FIGURE 10:**
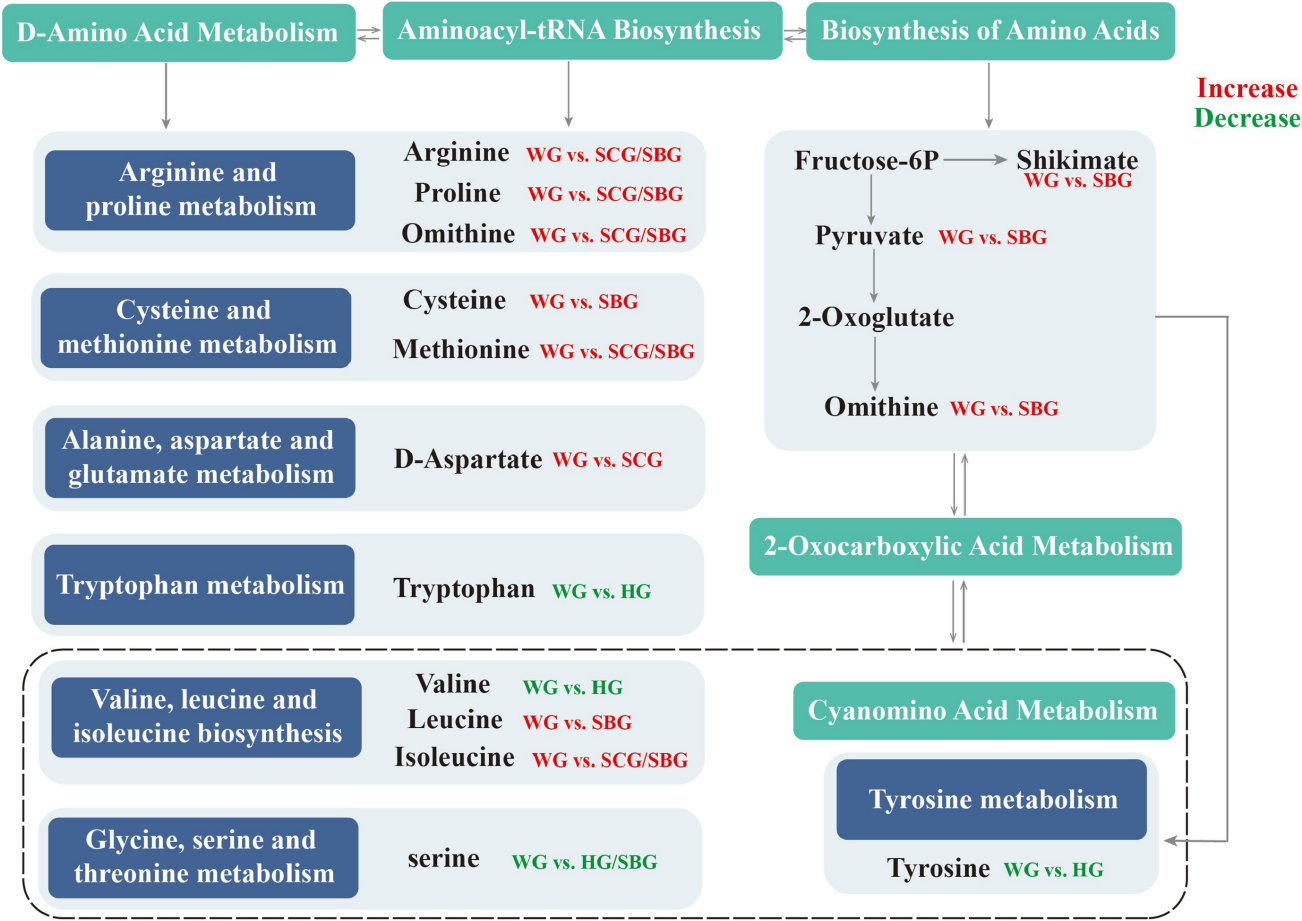
Expression of differential metabolites in amino acid biosynthesis and metabolism‐related pathways.

In addition to the KEGG analysis findings, the pathways primarily encompass the synthesis and metabolism of amino acids. The pathways within the WG versus HG, WG versus SCG, and WG versus SBG groups are listed as follows: aminoacyl‐tRNA biosynthesis, biosynthesis of amino acids, D‐amino acid metabolism, and cyanoamino acid metabolism. The above four metabolic pathways affect the pathways of various amino acids, such as valine, leucine, and isoleucine biosynthesis; glycine, serine, and threonine metabolism; tyrosine metabolism; arginine and proline metabolism; and cysteine and methionine metabolism (Figure 10), causing differences in amino acid formation among different garlic varieties. Plants need amino acids for a variety of metabolic activities, not only to produce proteins, but also to produce secondary metabolites and to transfer organic nitrogen between organs (Dinkeloo et al., 2018). Amino acids are closely related to the adaptability of garlic to environmental stress (Campalans et al., 1999). As a general response to the abiotic stress, plants, including halophytes, accumulate amino acids, betaine acid, sugars, organic acids, and other osmotic fluids in the cytoplasm (Parida & Das, 2005). Numerous studies have highlighted the roles of threonine, leucine, isoleucine, and other amino acids in enhancing plant resistance to environmental stress such as drought, temperature, salinity, etc. (Joshi et al., 2010). Additionally, the tropane, piperidine, and pyridine alkaloid biosynthesis pathway in WG versus SCG indicates a broader spectrum of phytochemicals beyond the more well‐known sulfur‐containing compounds. Alkaloids, including those in the tropane, piperidine, and pyridine classes, often function as secondary metabolites that contribute to the plant's ability to deter herbivores or defend against microbial infections. The presence of these alkaloids may contribute to the plant's defense mechanisms and environmental adaptation, and potentially offer additional nutritional or medicinal benefits (Zhang et al., 2022).

Meanwhile, ABC transporters were significantly enriched in WG versus HG, WG versus SCG, and WG versus SBG groups the three groups. ABC transporters facilitate the transport of various substances, including metal ions, lipids, proteins, etc., by hydrolyzing the energy released by ATP (Banasiak & Jasiński, 2021). These transporters play a key role in cellular tolerance and resistance to toxic substances (Tawbeh et al., 2021). Given that biological processes are dynamic and interconnected, the impact of stress factors on one component of this network can indirectly influence other pathways, either positively or negatively. Further research is necessary to investigate the specific alkaloids present in garlic and their potential implications for human health.

By conducting a comprehensive analysis of the pathways involved in differential metabolites, including both enrichment and topology analyses, we can further screen the pathways and identify the key pathways with the highest correlation with metabolite differences. Notably, the α‐linolenic/linoleic acid metabolism pathway was observed, along with significant enrichment in the cysteine and methionine metabolism pathway in the WG versus SBG group. Cysteine and methionine were the sulfur‐containing amino acids crucial for the synthesis of various bioactive compounds, including glutathione, allicin, methyl allyl disulfide, diallyl sulfide, and methionine, which play roles in defending against oxidative stress (Lee & Gladyshev, 2011). Variations in cysteine and methionine metabolism may impact the overall antioxidant capacity of garlics, influencing their health‐promoting properties. The glycine, serine, and threonine metabolism pathways indicate differences in the synthesis of these amino acids between WG and HG. These amino acids are essential in building blocks for protein synthesis and play important roles in various cellular processes. Variances in these pathways may influence the overall amino acid composition and nutritional content of the garlic varieties. In the comparative analyses of WG versus HG and WG versus SBG groups, significant enrichment was observed in the valine, leucine, and isoleucine biosynthesis pathways. These branched‐chain amino acids (BCAAs) are critical for protein synthesis and serve as precursors for various bioactive molecules (Galili et al., 2016), further supporting the conclusions drawn from the analysis.

### 3.8 Element contents in garlics

**FIGURE 11:**
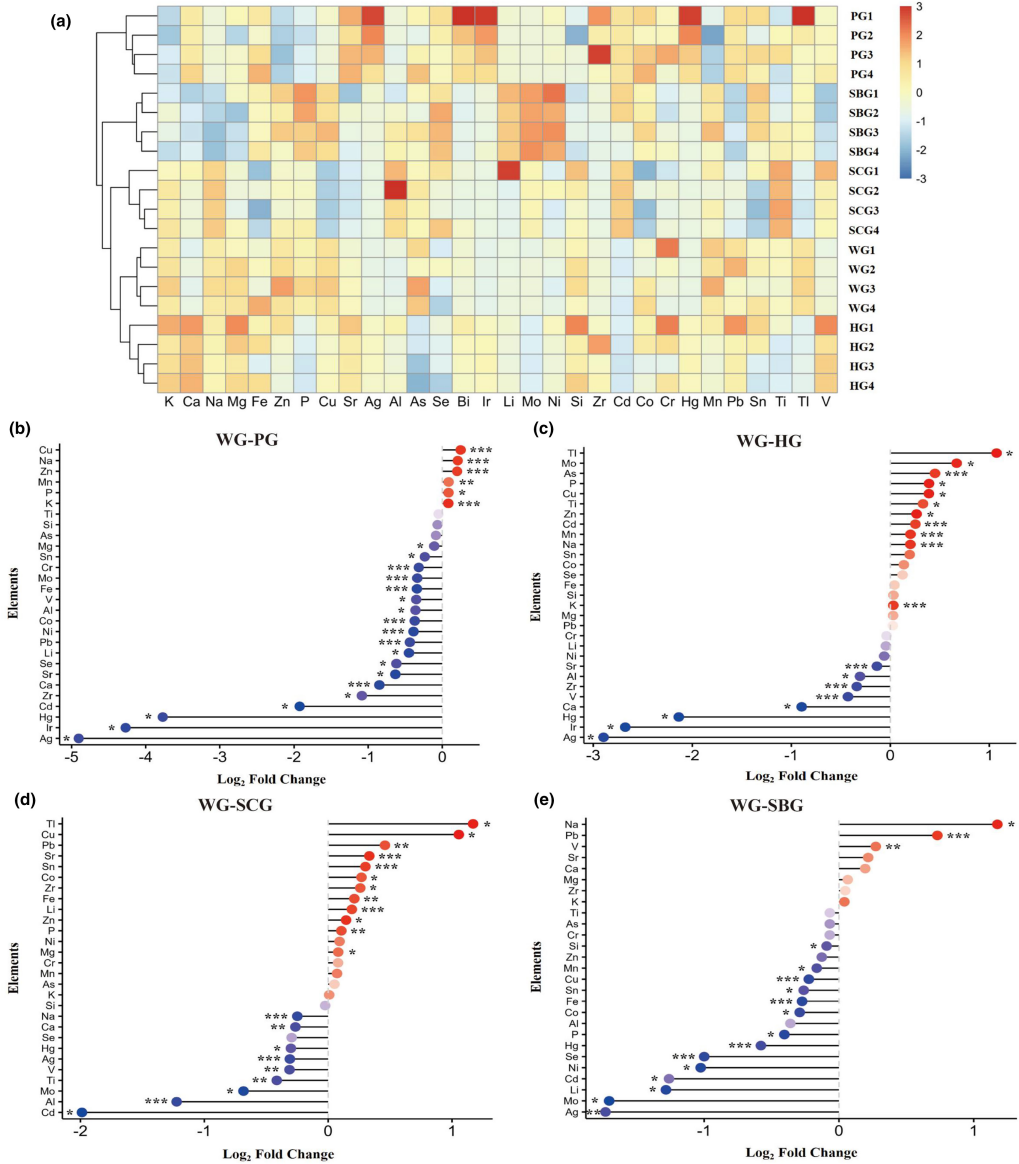
Heatmap of hierarchical clustering analysis and matchstick plot of differential ions in five garlic varieties. (a) Heatmap of hierarchical clustering analysis. (b) Matchstick plot of differential ions in WG versus PG; (c) matchstick plot of differential ions in WG versus HG; (d) matchstick plot of differential ions in WG versus SCG; and (e) matchstick plot of differential ions in WG versus SBG.

The concentrations of 30 elements in five distinct garlic varieties are depicted in Figure 11a. These elements encompassed 9 nutrient elements, 5 metal elements, 10 heavy metal elements, 4 alkali metal elements, and 2 non‐metallic elements. Notably, there are variations in the content of nutrients, alkali metals, other metal elements, and heavy metals across different garlic varieties. The concentration of heavy metals in all garlic varieties is significantly below the threshold established by the National Food Safety Standard for heavy metal detection. These results indicate that the analyzed elements vary significantly among the five garlic varieties, each of which is grown in the same region.

Subsequent pairwise comparison tests demonstrate that the concentrations of certain elements in garlic samples differ compared to WG samples. Analyzing the elements of WG compared with the other four types of garlic, 17 elements, including Fe, Ca, Ni, K, Cu, Na, Co, Zn, Zr, etc., exhibited significance with both *p* < .05 and VIP > 1 among the WG versus PG group (Table S2). 19 elements, including K, Mn, Na, As, V, Cd, Sr, Zr, etc., exhibited significance with both *p* < .05 and VIP > 1 among the WG versus HG group (Table S3). 19 elements, including Sr, Na, Al, Sn, Ag, Li, P, Fe, Ca, Ti, etc., exhibited significance with both *p* < .05 and VIP > 1 among the WG versus SCG group (Table S4). 14 elements, including Cu, Fe, Se, Pb, Hg, Ag, Mn, Na, P, etc., exhibited significance with both *p* < .05 and VIP > 1 among the WG versus SBG group (Table S5). The elements identified in each pairwise comparison group were recognized as potential indicators for distinguishing between the four groups of garlic bulbs (Ahn et al., 2019; D'Archivio et al., 2019).

In the pairwise comparison results and discussion, we identified 27 elements that simultaneously met the criteria of *p* < .05 and VIP > 1. Further analysis reveals differential expression of detected elements among the five garlic varieties. Compared to PG, four elements were significantly upregulated and seven elements were significantly downregulated in WG (Figure 11b). Compared to HG, five elements were significantly upregulated and three elements were significantly downregulated in WG (Figure 11c). Compared to SCG, three elements were significantly upregulated and three elements were significantly downregulated in WG (Figure 11d). Compared to SBG, one element was significantly upregulated and four elements were significantly downregulated (Figure 11e).

In summary, garlic, being a plant possessing numerous advantageous properties for human health, exhibits a wide array of metabolites. Different garlic varieties possess distinct metabolites and nutrients, each offering substantial advantages for human health. This has led to increasing attention and consumption of garlic products. Our research has revealed the metabolic characteristics of several types of garlic. Among these, WG, PG, and HG have similar shapes and sizes but different colors, whereas SCG, SBG, and WG exhibit significant differences in appearance and size. Metabolomic analysis indicates that all types of garlic contain abundant metabolites, with WG being particularly rich in amino acids and demonstrating a strong ability to resist environmental stress. Preliminary findings suggest that garlic extract may offer significant antioxidant, free radical scavenging, antibacterial, and anti‐inflammatory effects. Notably, the levels and types of active ingredients in garlic may vary depending on factors such as garlic variety and cultivation conditions. Further research is needed to explore the bioavailability and potential beneficial interactions of these compounds in the human body.

## 4 CONCLUSION

In summary, a comprehensive examination of the metabolites in five garlic varieties was carried out using nontargeted metabolomics and ionomics. A total of 30 chemical elements and 1256 metabolites were identified. Across these assessments, a total of 1234 (WG vs. PG), 1244 (WG vs. HG), 1247 (WG vs. SCG), and 1255 (WG vs. SBG) compounds were identified in five different garlics. The metabolomics data in the study reveal the rich diversity of compounds present in garlic. By comparing the differential compounds among the four groups, WG, PG, and HG have similar shapes and sizes but different colors, whereas SCG, SBG, and WG exhibit significant differences in appearance and size. KEGG analysis revealed key metabolic pathways related to amino acid metabolism and synthesis. Data from ionomics analysis indicate that garlic contains a variety of trace elements essential for the human body, making it highly nutritious. The diversity of metabolites not only contributes to the unique taste and aroma of garlic but also has an impact on its potential medicinal and nutritional value.

## AUTHOR CONTRIBUTIONS

**Junjun Meng:** Conceptualization (equal); data curation (equal); formal analysis (equal); funding acquisition (equal); investigation (equal); writing – original draft (equal). **Haitao Zhong:** Methodology (equal); validation (equal); writing – review and editing (equal). **Xue Chu:** Methodology (equal); validation (equal); writing – review and editing (equal). **Jinxiu Guo:** Data curation (equal); funding acquisition (equal); methodology (equal); validation (equal); writing – review and editing (equal). **Shiyuan Zhao:** Data curation (equal); funding acquisition (equal); methodology (equal); validation (equal); writing – review and editing (equal). **Tao Shen:** Formal analysis; data curation; writing‐review & editing. **Wenxue Sun:** Data curation (equal); funding acquisition (equal); methodology (equal); validation (equal); writing – review and editing (equal). **Jianhua Wang:** Data curation (equal); methodology (equal); validation (equal); writing – review and editing (equal). **Pei Jiang:** Conceptualization (equal); formal analysis (equal); methodology (equal); project administration (equal); supervision (equal); validation (equal); writing – original draft (equal); writing – review and editing (equal).

## CONFLICT OF INTEREST STATEMENT

The authors declare that they have no known competing financial interests or personal relationships that could have appeared to influence the work reported in this paper.

## Supporting information


Supinfo S1.

## Data Availability

Data will be made available on request.
