# Supplementary material for: Comparative analysis of chemical elements and metabolites in diverse garlic varieties based on metabolomics and ionomics
Source: Food Sci Nutr. 2024 Aug 7;12(10):7719–36. doi: 10.1002/fsn3.4397 (PMC11521672; doi:10.1002/fsn3.4397)
Supplement: Supplementary file 1 — Supinfo S1. [file FSN3-12-7719-s001.docx]

**Comparative Analysis of Chemical Elements and Metabolites in Diverse Garlic Varieties Based on Metabolomics and Ionomics**

Junjun Meng ^1, #^, Tao Shen ^2, #^, Xue Chu ^1^, Jinxiu Guo ^1^, Shiyuan Zhao ^1^,

Wenxue Sun ^1^, Jianhua Wang ^1^, Haitao Zhong ^1, *^, Pei Jiang^1, *^

^1^ Translational Pharmaceutical Laboratory, Jining NO.1 People's Hospital, Shandong First Medical University, Jiankang Road, Jining 272000, China

^2^ Shandong Engineering Research Center for Traditional Chinese Medicine Standard, School of Pharmaceutical Sciences, Cheeloo College of Medicine, Shandong University, Jinan, 250012 , China

* **Corresponding authors:** Haitao Zhong, Pei Jiang

**E-mail:** [haitao_zhong@163.com](mailto:haitao_zhong@163.com) (H. Zhong), [jiangpeicsu@sina.com](mailto:jiangpeicsu@sina.com) (P. Jiang)

**Tel./Fax:** +86 537 2337200, +86 537 2106208

Translational Pharmaceutical Laboratory, Jining NO.1 People's Hospital, Shandong First Medical University, Jiankang Road, Jining 272000, China

# These authors contributed equally to this work.

| **Table S1** Mean relative quantification values of 14 amino acids in an experimental group compared to 5 garlic varieties. | | | | | | | | | |
| --- | --- | --- | --- | --- | --- | --- | --- | --- | --- |
| **NO.** | **MS2 name** | **mz** | **Mean WG** | **Mean PG** | **Mean HG** | **Mean SCG** | **Mean SBG** | **P-value** | **Q-value** |
| 1 | **l-threonine** | 118.0509407 | 15.06942842 | 17.02602803 | 13.65958276 | 17.36416494 | 12.65722187 | 3.95236E-05 | 6.8004E-05 |
| 2 | **l-hydroxyproline** | 132.0653076 | 2.722942107 | 14.44714869 | 1.236488663 | 1.664191105 | 2.355821136 | 5.13112E-23 | 1.16446E-19 |
| 3 | **l-phenylalanine** | 166.0861094 | 6.175075336 | 6.207979984 | 7.330042945 | 9.447988721 | 9.218278186 | 0.000233731 | 0.0003481 |
| 4 | **l-arginine** | 173.1040021 | 119.5849428 | 110.6702748 | 150.350294 | 184.1687651 | 231.7189561 | 2.39799E-08 | 1.08322E-07 |
| 5 | **2-aminoacrylic acid** | 88.03916498 | 1.41233406 | 1.259617007 | 1.253694062 | 1.762600264 | 2.675142109 | 1.22425E-07 | 4.2978E-07 |
| 6 | **l-serine** | 106.0497237 | 0.975762672 | 0.888352433 | 0.556141738 | 1.137161922 | 0.528852425 | 1.20644E-08 | 6.06738E-08 |
| 7 | **cysteine** | 120.012333 | 0.426413729 | 0.349981943 | 0.481530322 | 0.609369094 | 1.631125452 | 2.26236E-10 | 2.01325E-09 |
| 8 | **l-proline** | 116.0704655 | 2.129256272 | 1.853989276 | 2.019613762 | 3.34462901 | 3.772979585 | 0.000839707 | 0.001144934 |
| 9 | **cysteate** | 167.9968331 | 0.957953056 | 1.13030088 | 1.52962708 | 1.72019139 | 4.049138544 | 0.008868282 | 0.010703132 |
| 10 | **d-aspartic acid** | 134.0446655 | 4.604755036 | 4.504697066 | 4.603896322 | 6.757246987 | 4.984172178 | 0.000127196 | 0.000198546 |
| 11 | **l-lysine** | 147.1127238 | 2.898358464 | 3.59018717 | 2.564922971 | 3.073187016 | 2.594623327 | 0.004825911 | 0.005988048 |
| 12 | **l-aspartate** | 132.0301724 | 13.443662 | 13.15962175 | 11.99941835 | 17.18317861 | 10.83648378 | 0.000551561 | 0.000772844 |
| 13 | **l-ornithine** | 131.0825374 | 10.82212242 | 9.04922412 | 15.30174973 | 18.60966497 | 24.0340816 | 4.49264E-07 | 1.30502E-06 |
| 14 | **o-acetyl-l-serine** | 146.0457845 | 0.173476077 | 0.204732106 | 0.129911618 | 0.13616292 | 0.106285716 | 0.010414378 | 0.012478536 |


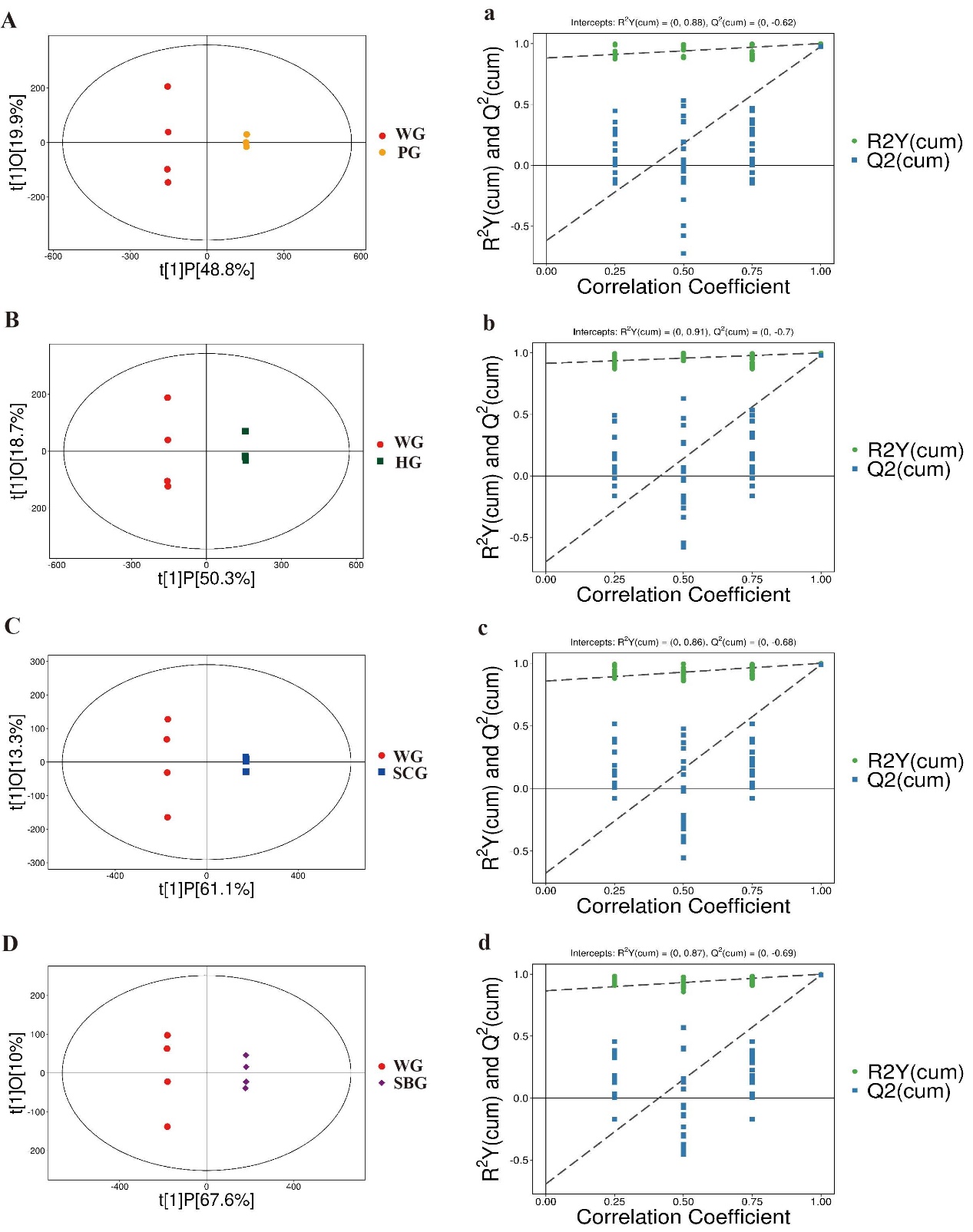


**Figure S1.** (**A-D**) Scatter plot of the OPLS−DA models. (**a-d**) Replacement test results of the OPLS−DA models


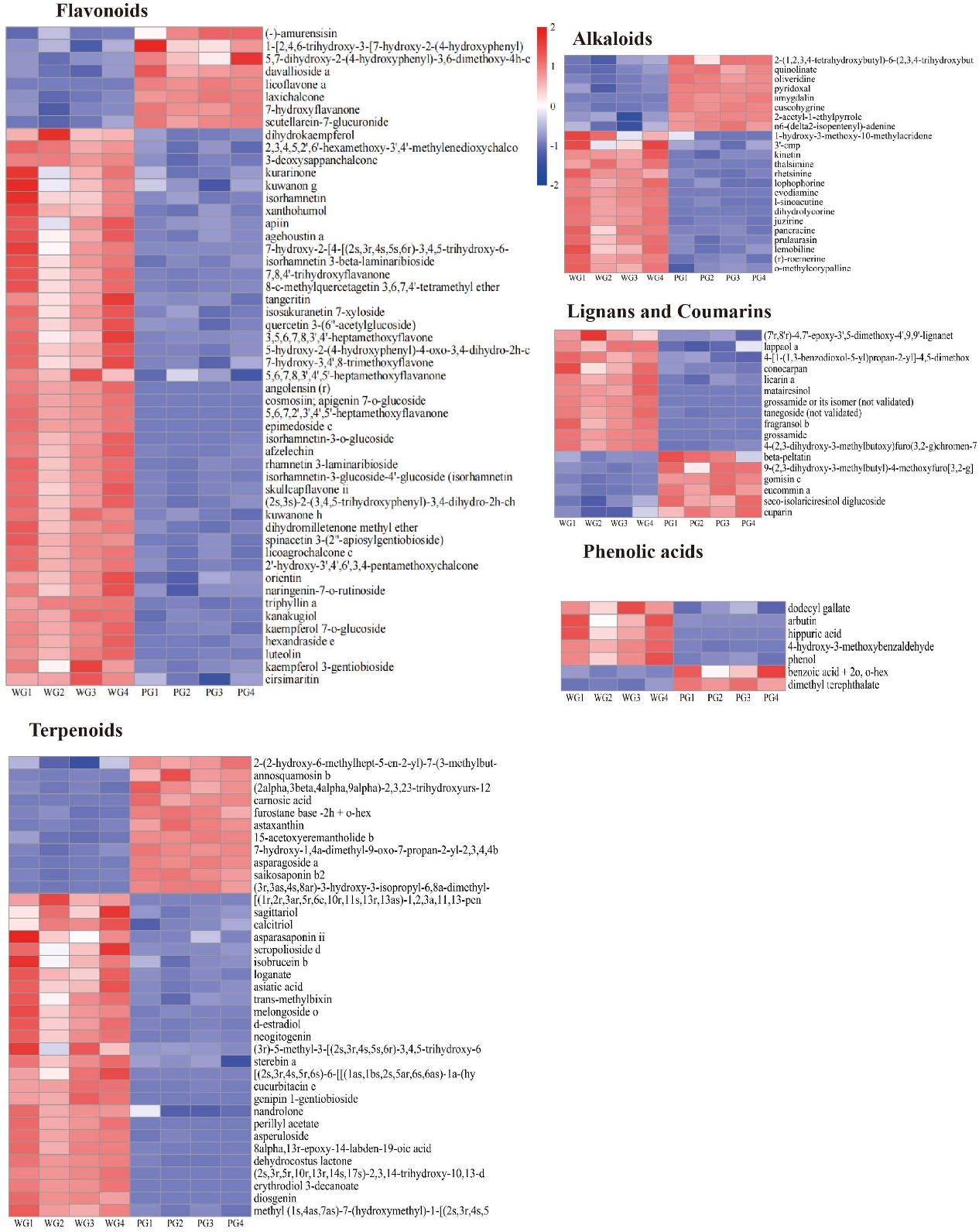


**Figure S2.** Clustering heatmaps of partial substances (WG vs. PG)


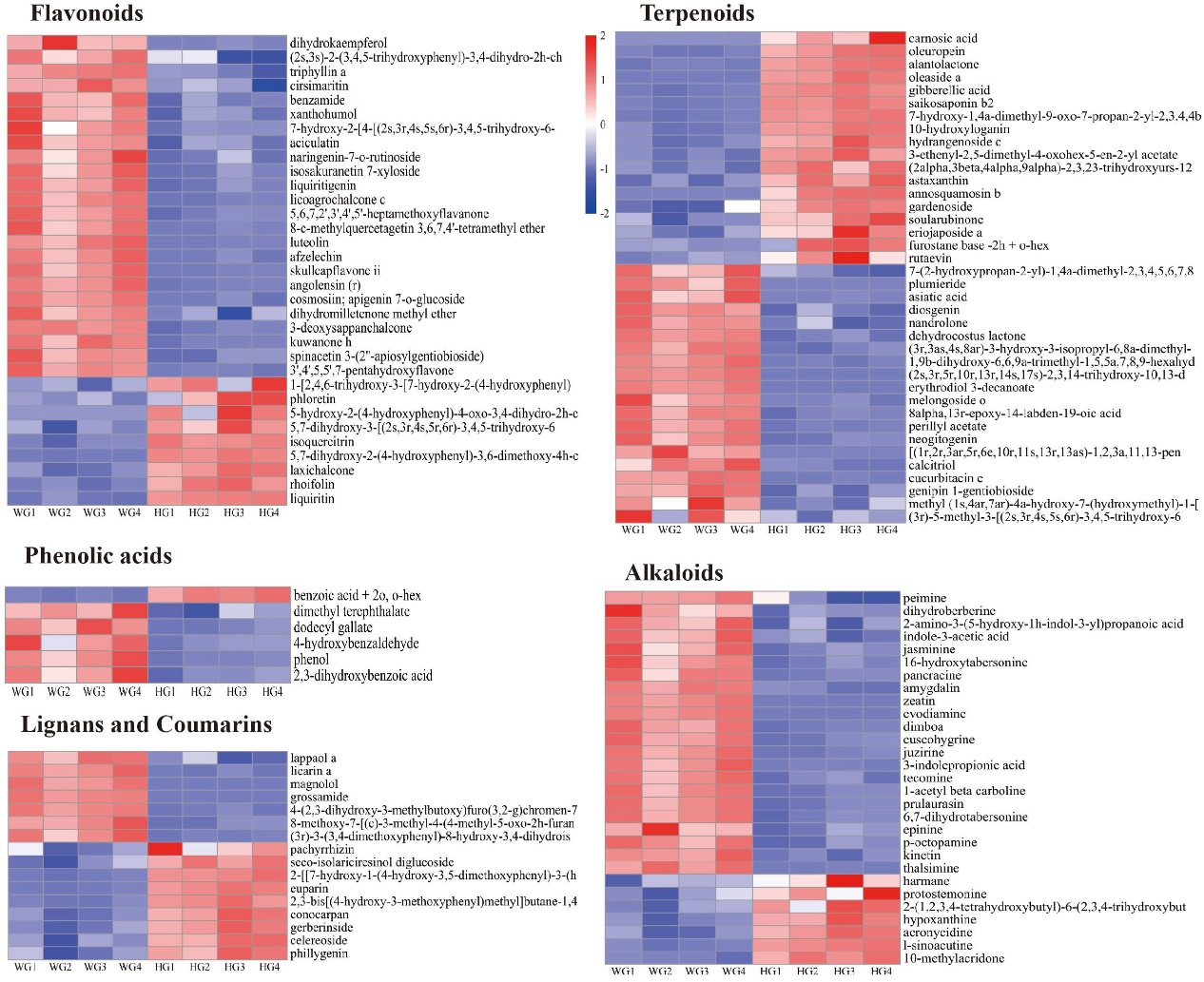


**Figure S3.** Clustering heatmaps of partial substances (WG vs. HG)


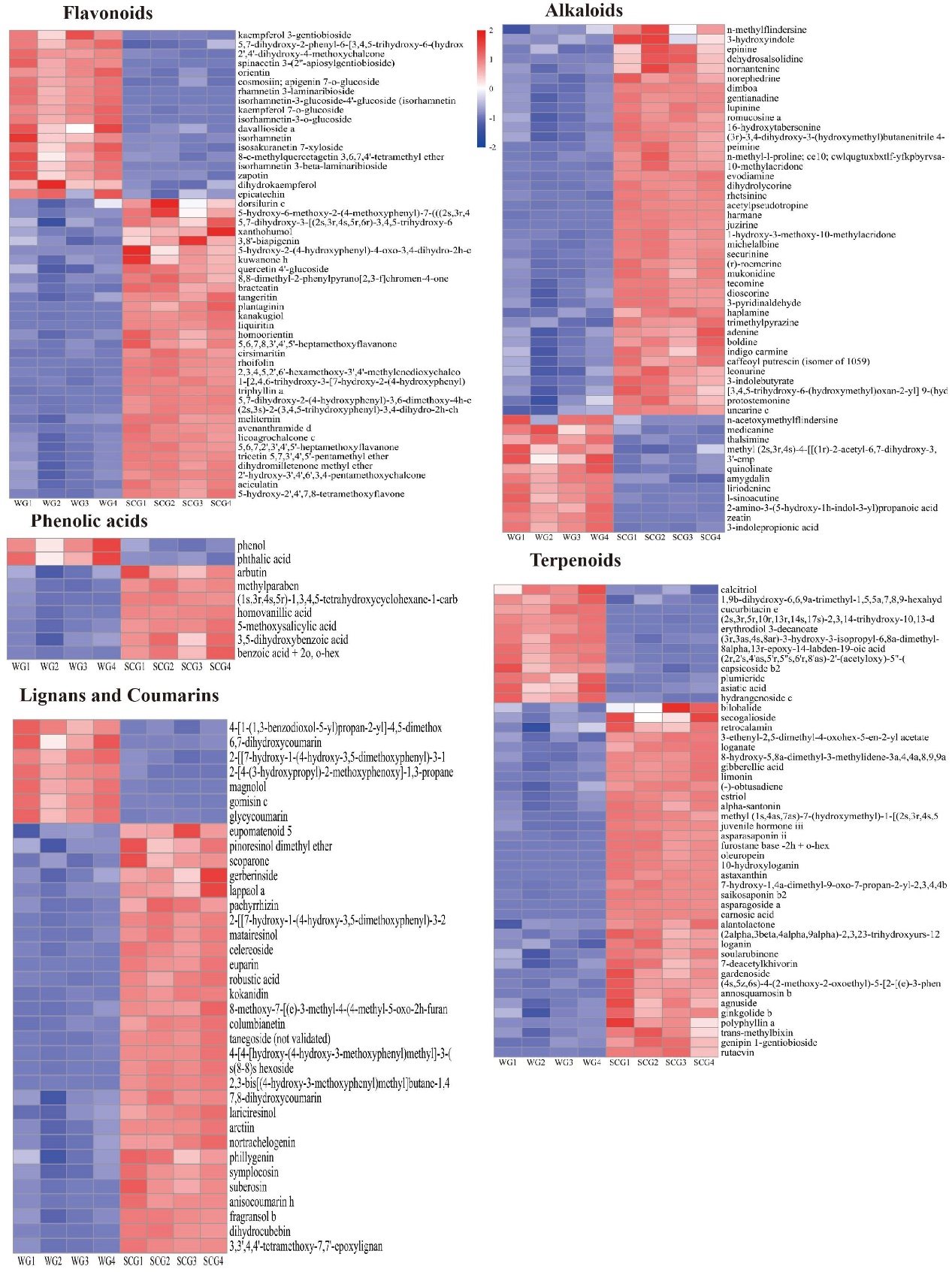


**Figure S4.** Clustering heatmaps of partial substances (WG vs. SCG)


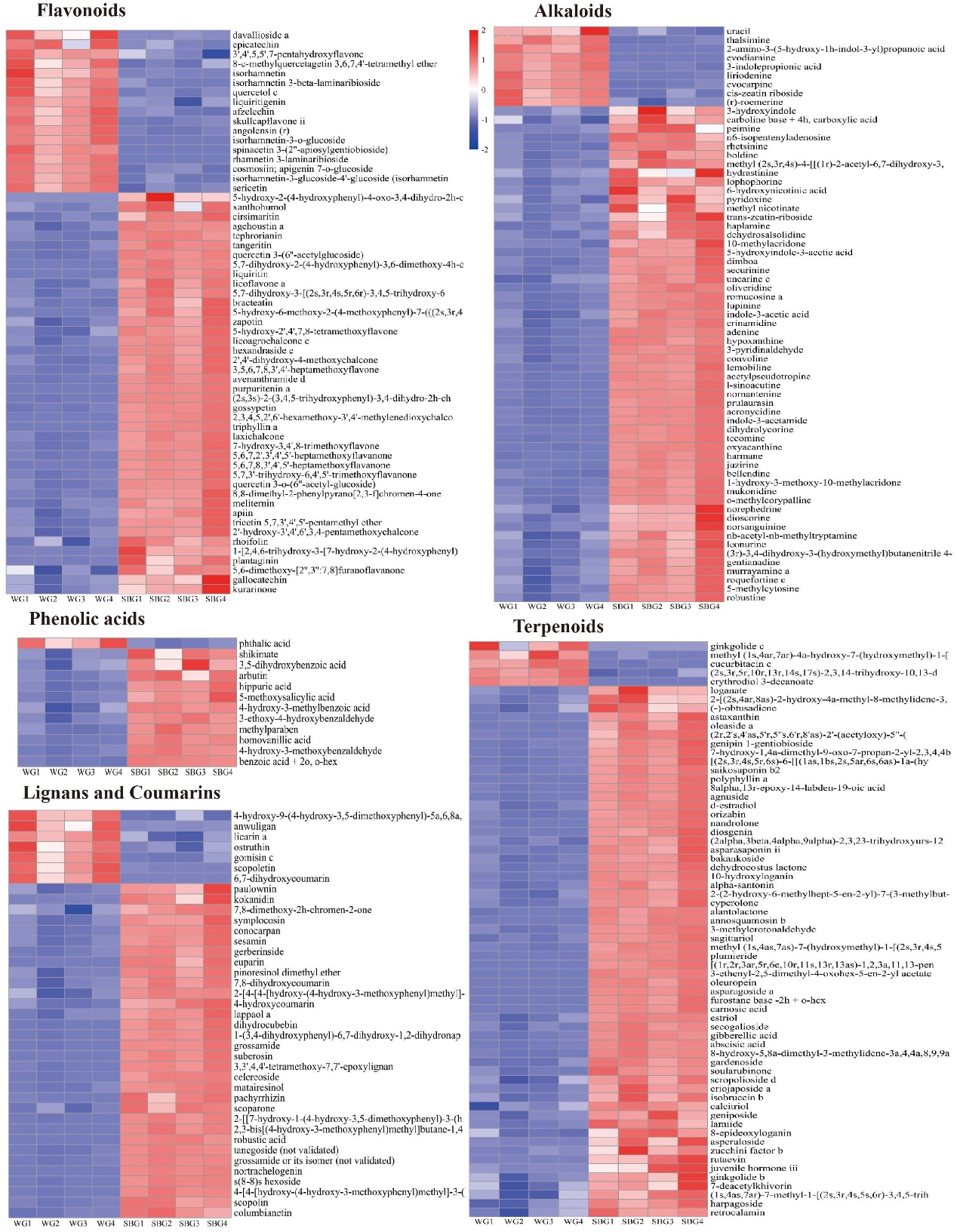


**Figure S5.** Clustering heatmaps of partial substances (WG vs. SBG)

**
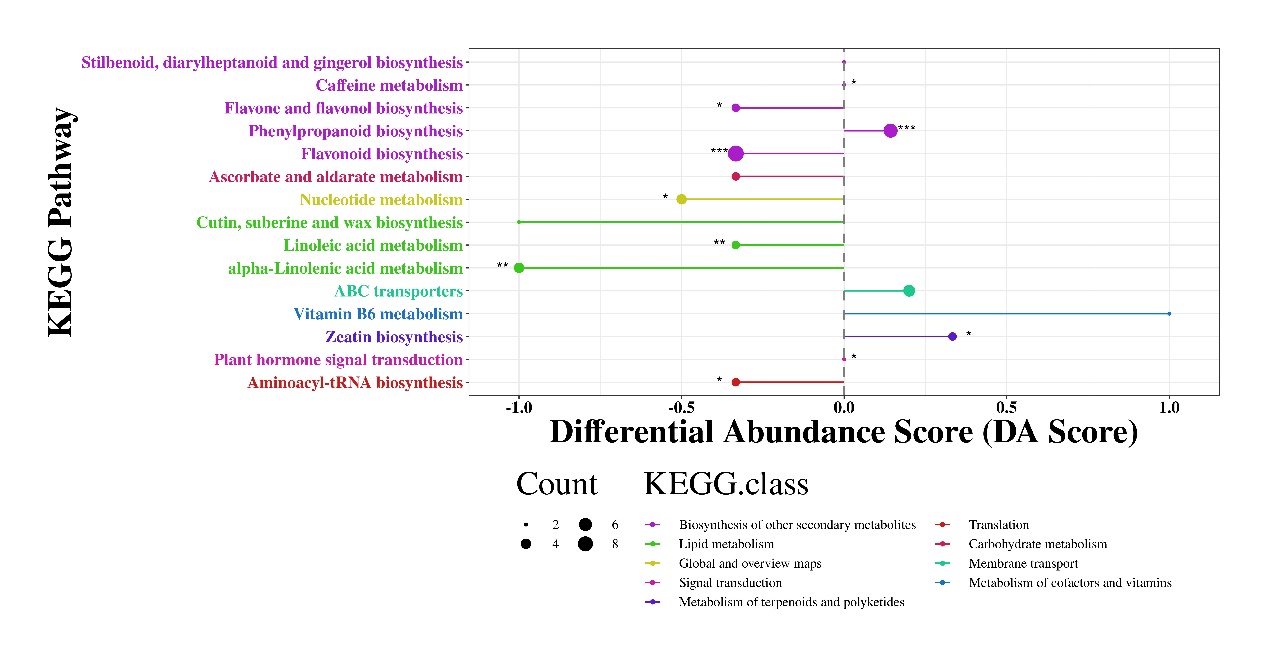
**

**Figure S6-1.** The enrichment analysis of KEGG DA score plot of WG vs. PG.


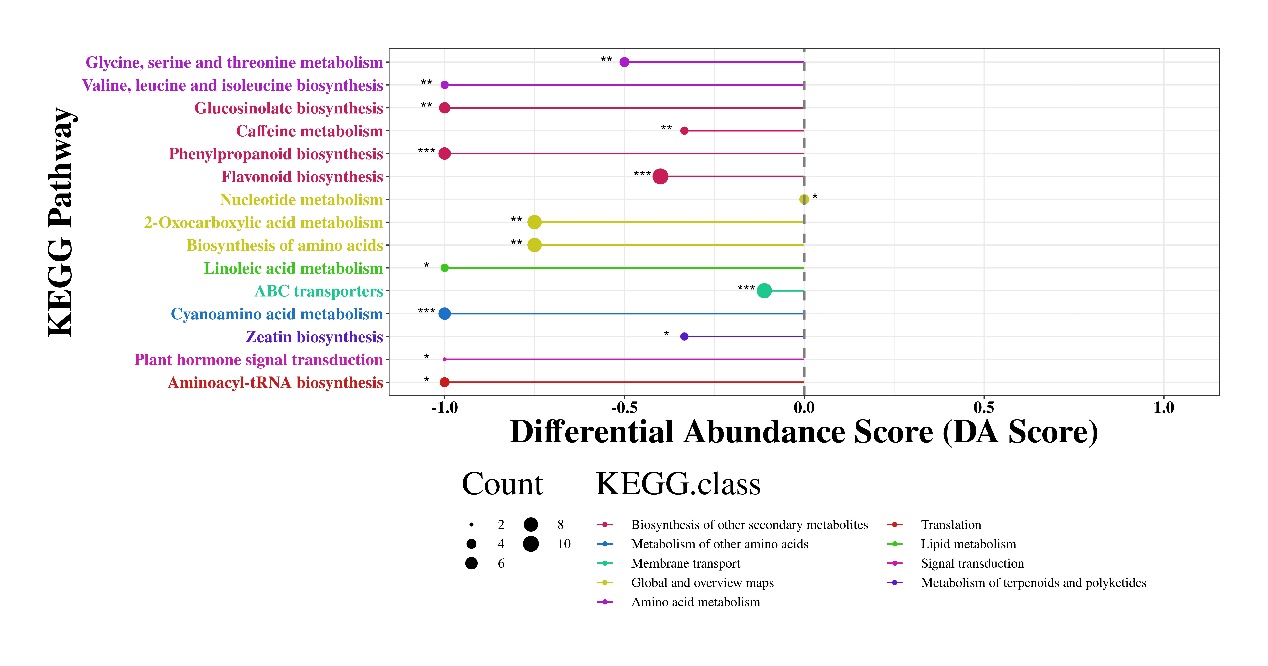


**Figure S6-2.** The enrichment analysis of KEGG DA score plot of WG vs. HG.

**
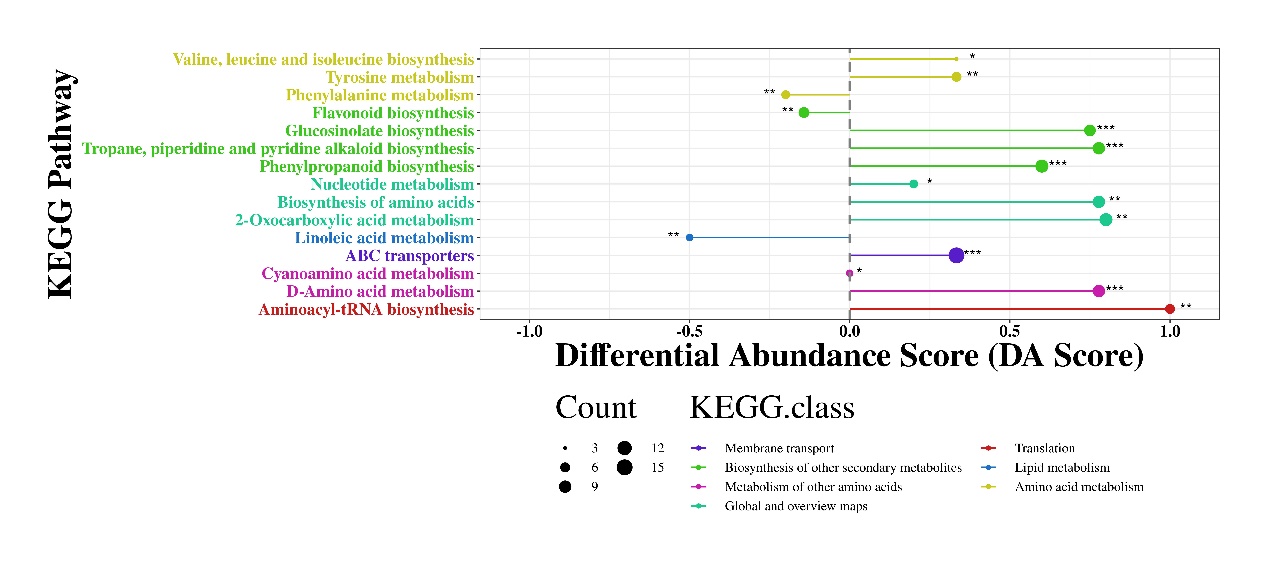
**

**Figure S6-3.** The enrichment analysis of KEGG DA score plot of WG vs. SCG.


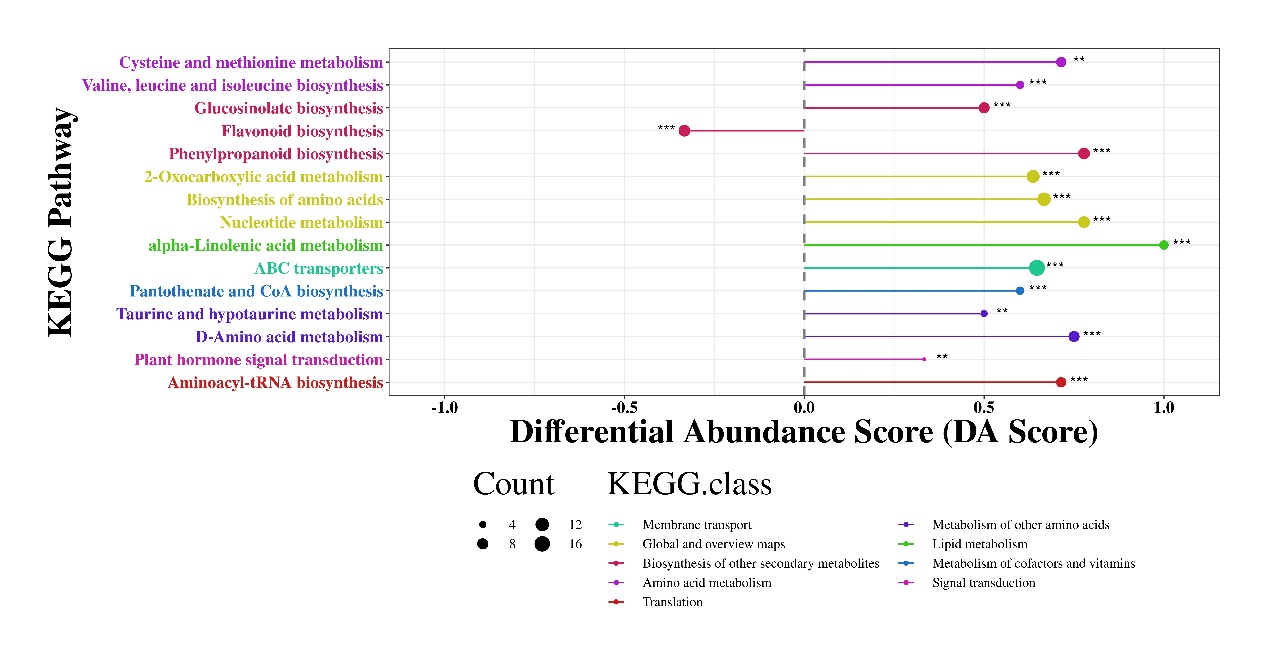


**Figure S6-4.** The enrichment analysis of KEGG DA score plot of WG vs. SBG.


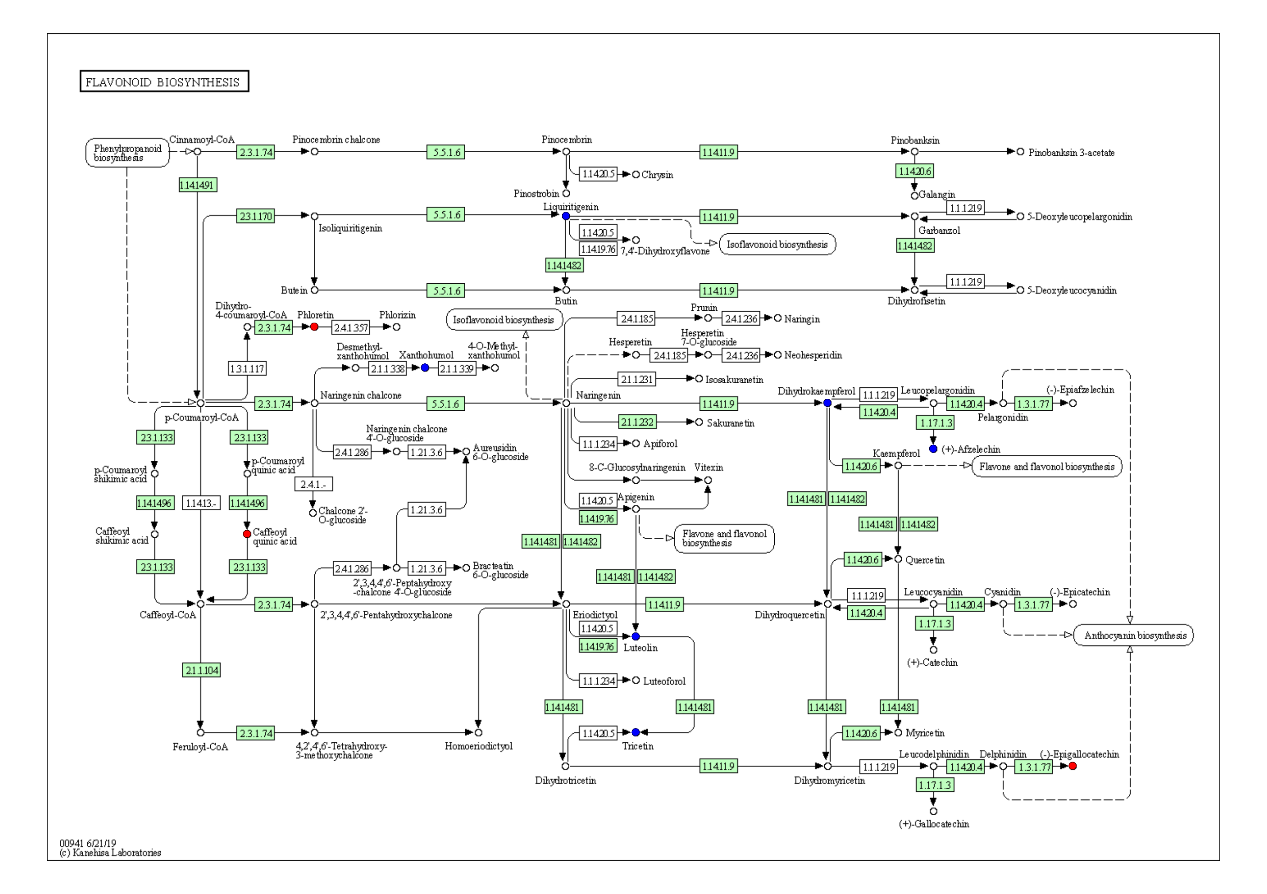


**Figure S7-1.** The flavonoid biosynthesis with significant enrichment of differential metabolites in the KEGG pathway analysis of the WG vs. PG group.


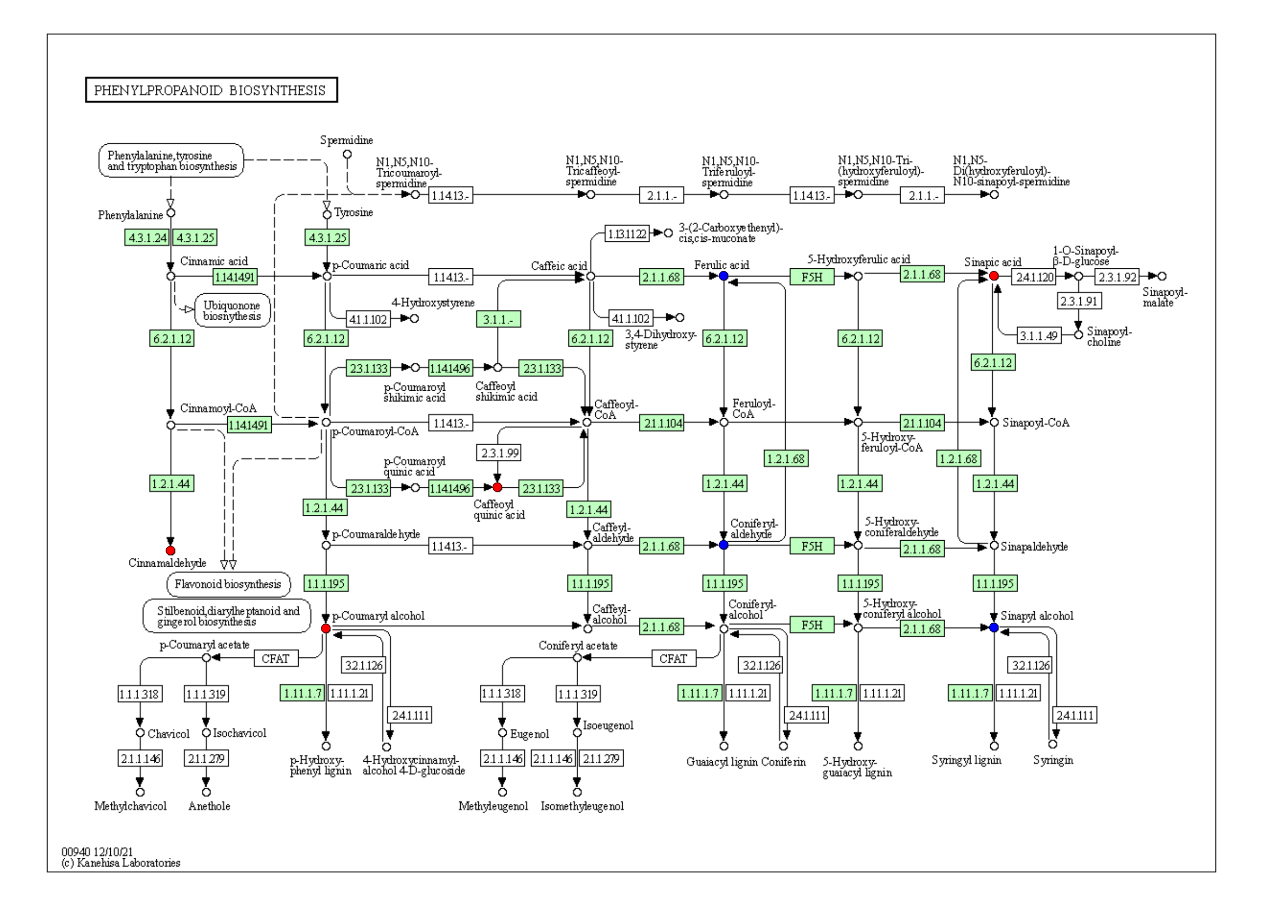


**Figure S7-2.** The phenylpropanoid biosynthesis with significant enrichment of differential metabolites in the KEGG pathway analysis of the WG vs. PG group.


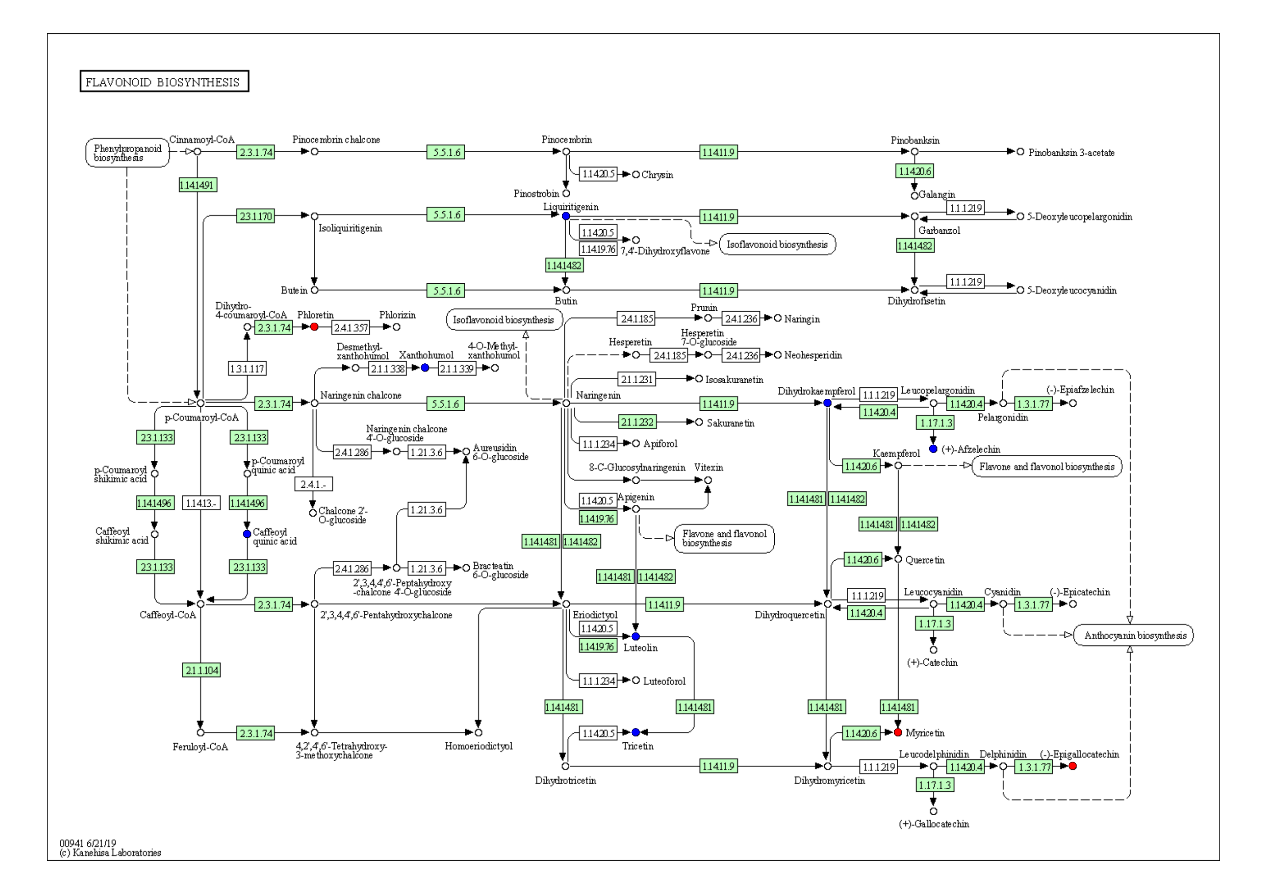


**Figure S8-1.** The flavonoid biosynthesis with significant enrichment of differential metabolites in the KEGG pathway analysis of the WG vs. HG group.
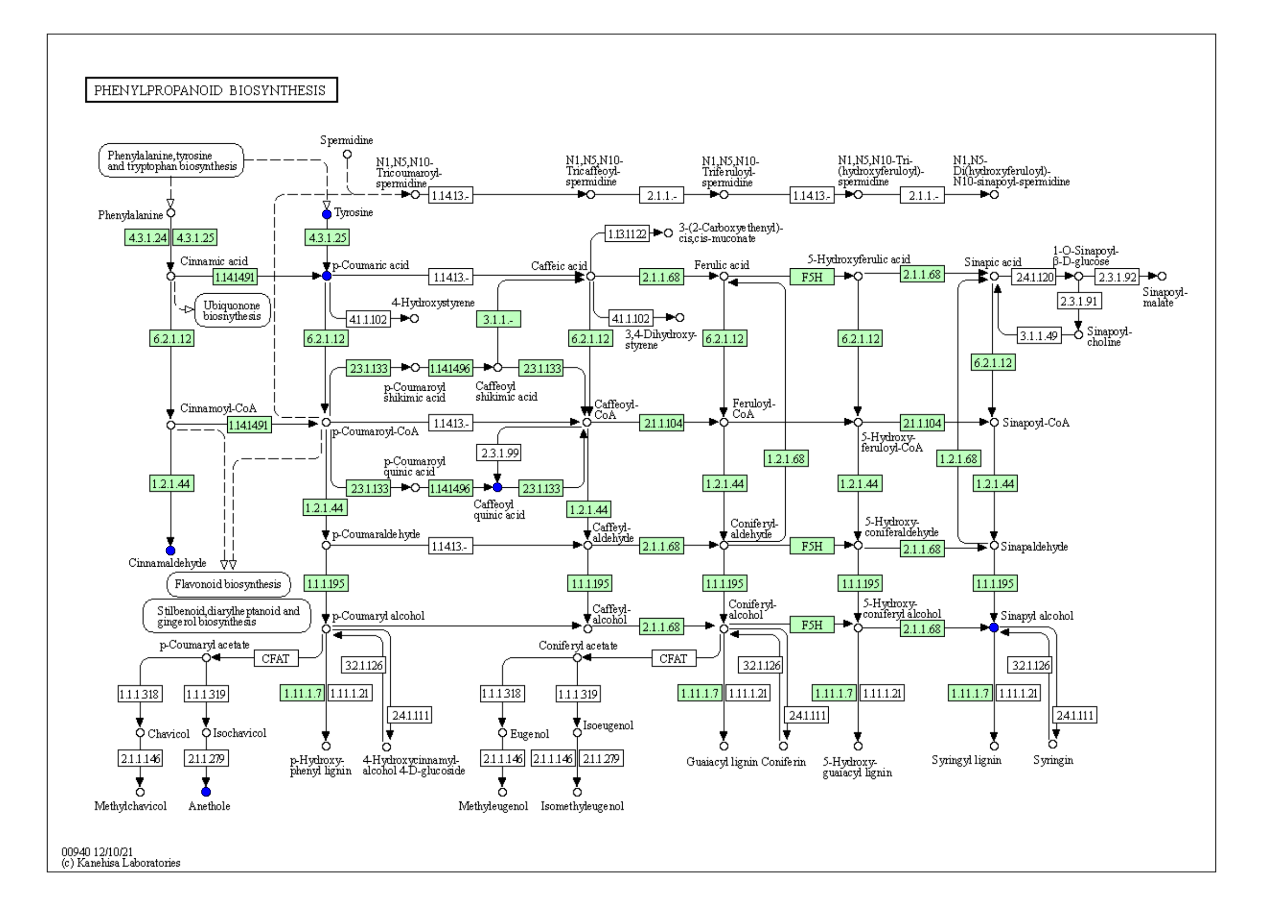


**Figure S8-2.** The phenylpropanoid biosynthesis with significant enrichment of differential metabolites in the KEGG pathway analysis of the WG vs. HG group.


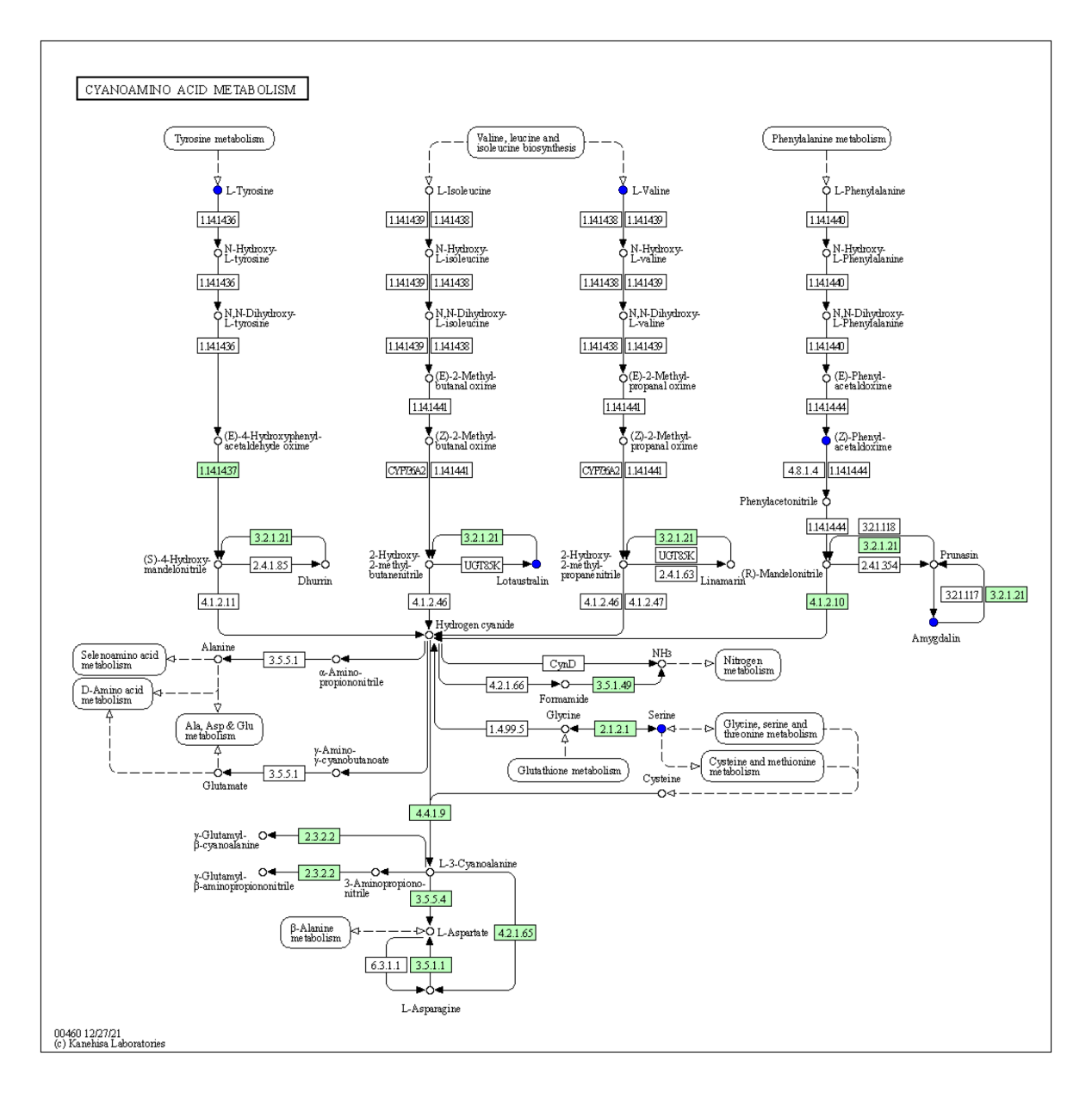


**Figure S8-3.** The cyanoamino acid metabolism with significant enrichment of differential metabolites in the KEGG pathway analysis of the WG vs. HG group.


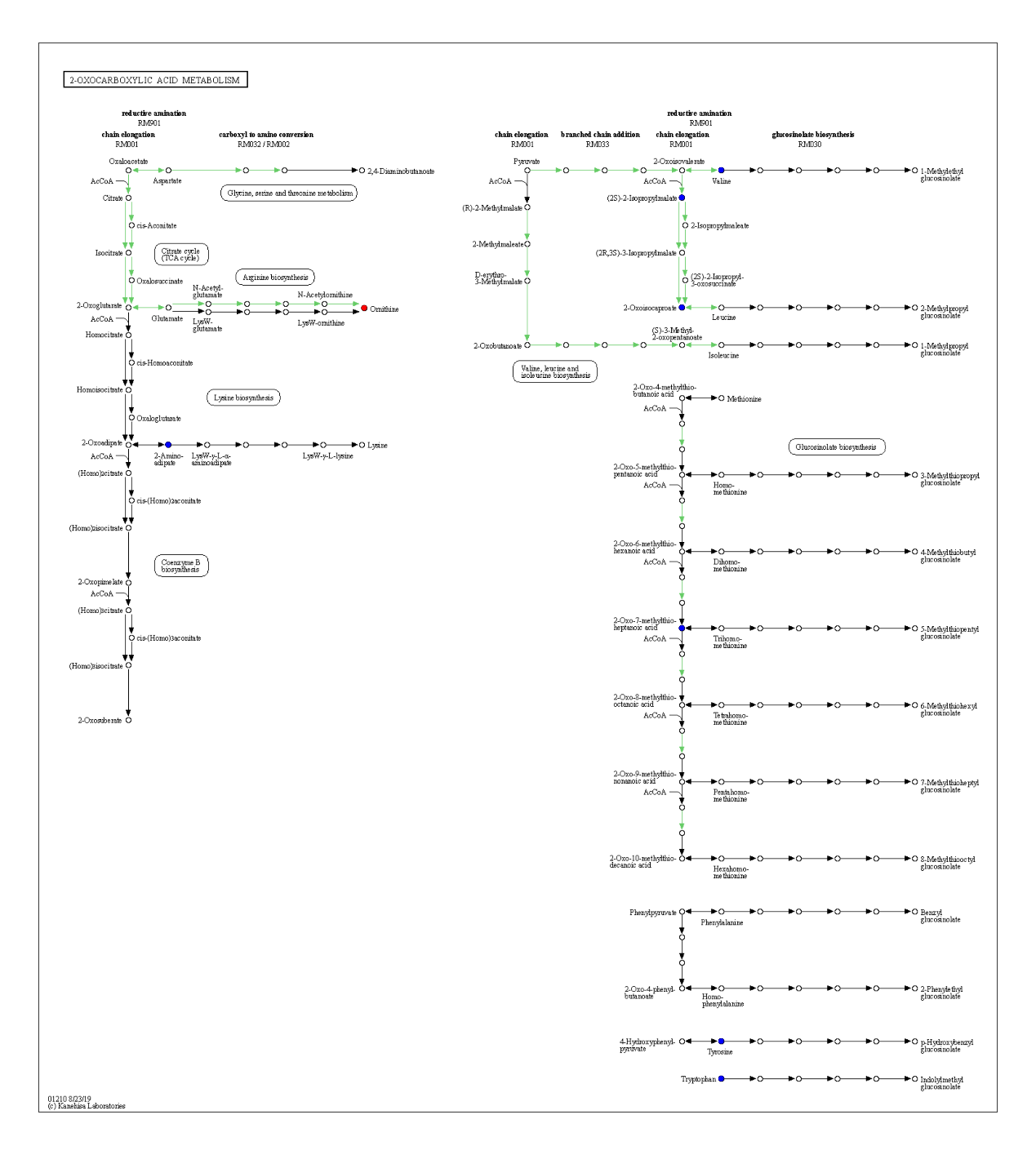


**Figure S8-4.** The 2-oxocarboxylic acid metabolism with significant enrichment of differential metabolites in the KEGG pathway analysis of the WG vs. HG group.


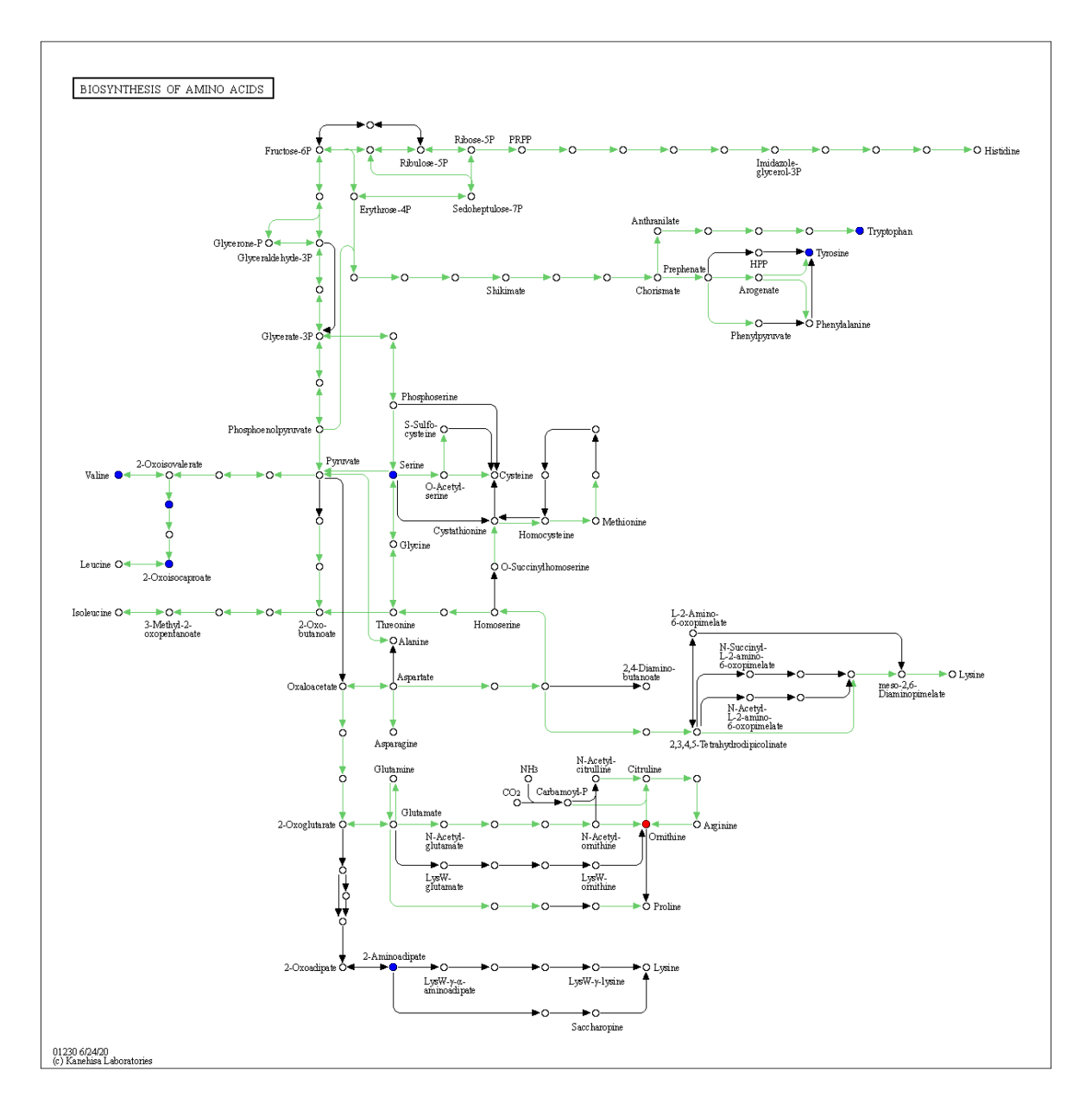


**Figure S8-5.** The biosynthesis of amino acids with significant enrichment of differential metabolites in the KEGG pathway analysis of the WG vs. HG group.


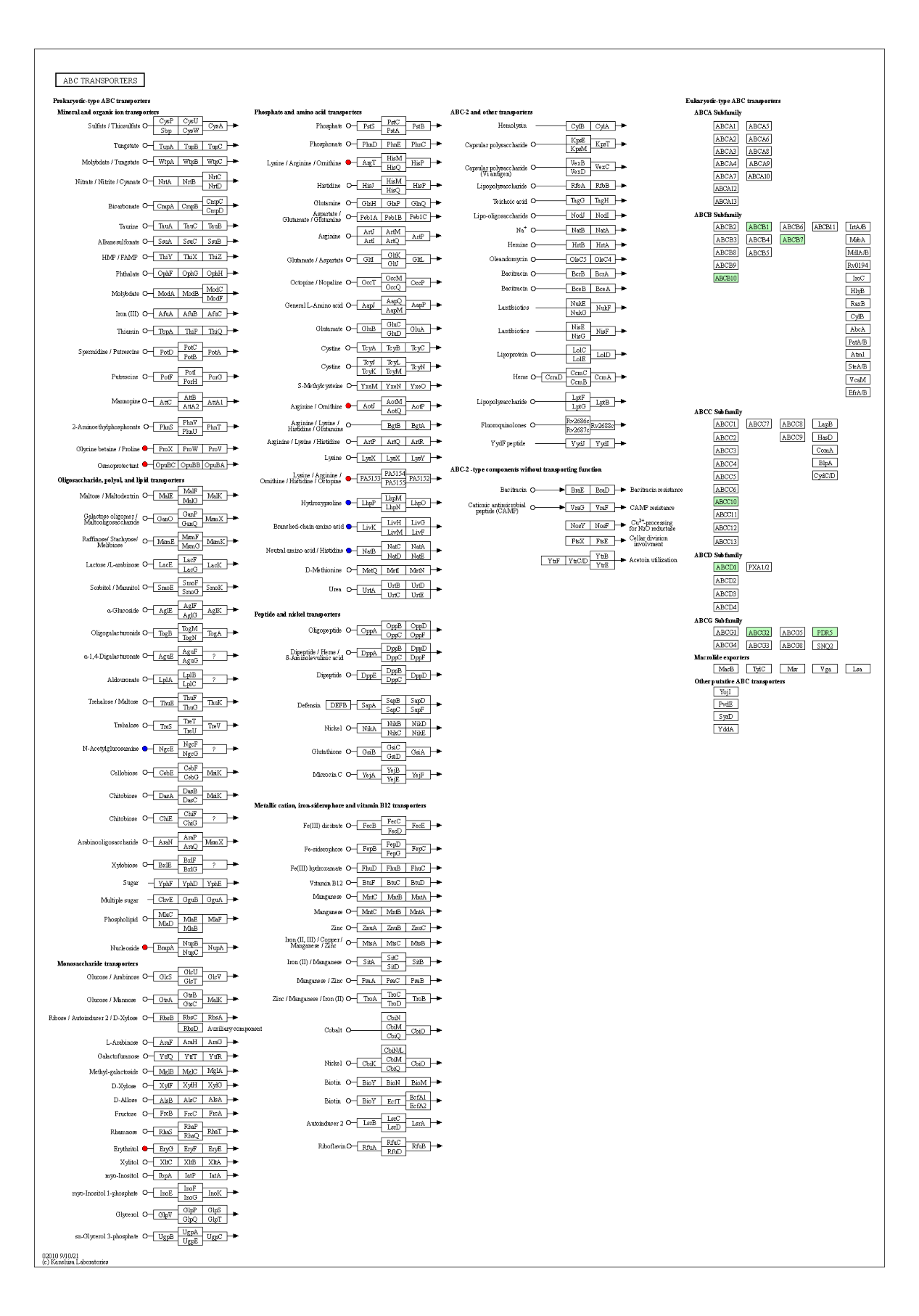


**Figure S8-6.** The ABC transporters with significant enrichment of differential metabolites in the KEGG pathway analysis of the WG vs. HG group.


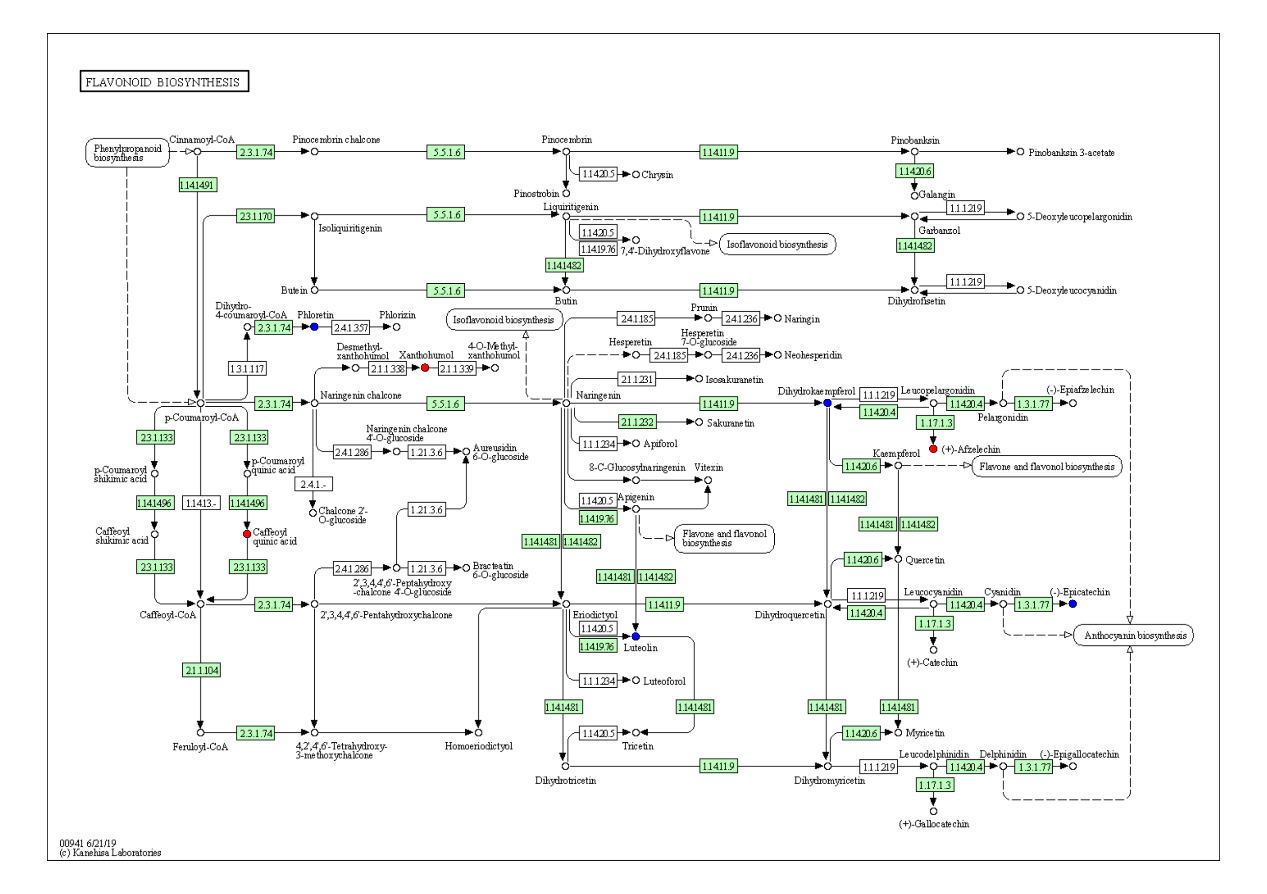


**Figure S9-1.** The flavonoid biosynthesis with significant enrichment of differential metabolites in the KEGG pathway analysis of the WG vs. SCG group.


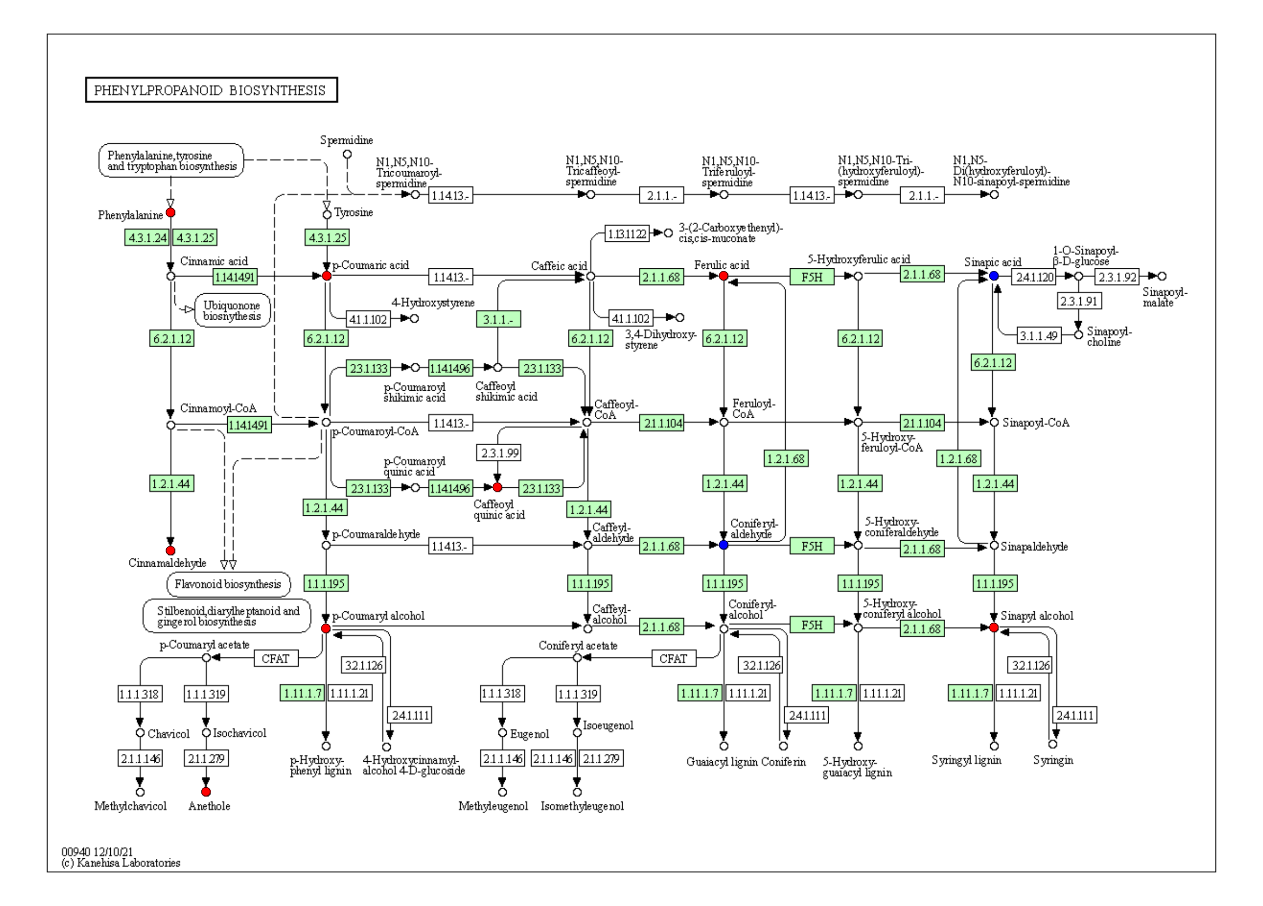


**Figure S9-2.** The phenylpropanoid biosynthesis with significant enrichment of differential metabolites in the KEGG pathway analysis of the WG vs. SCG group.


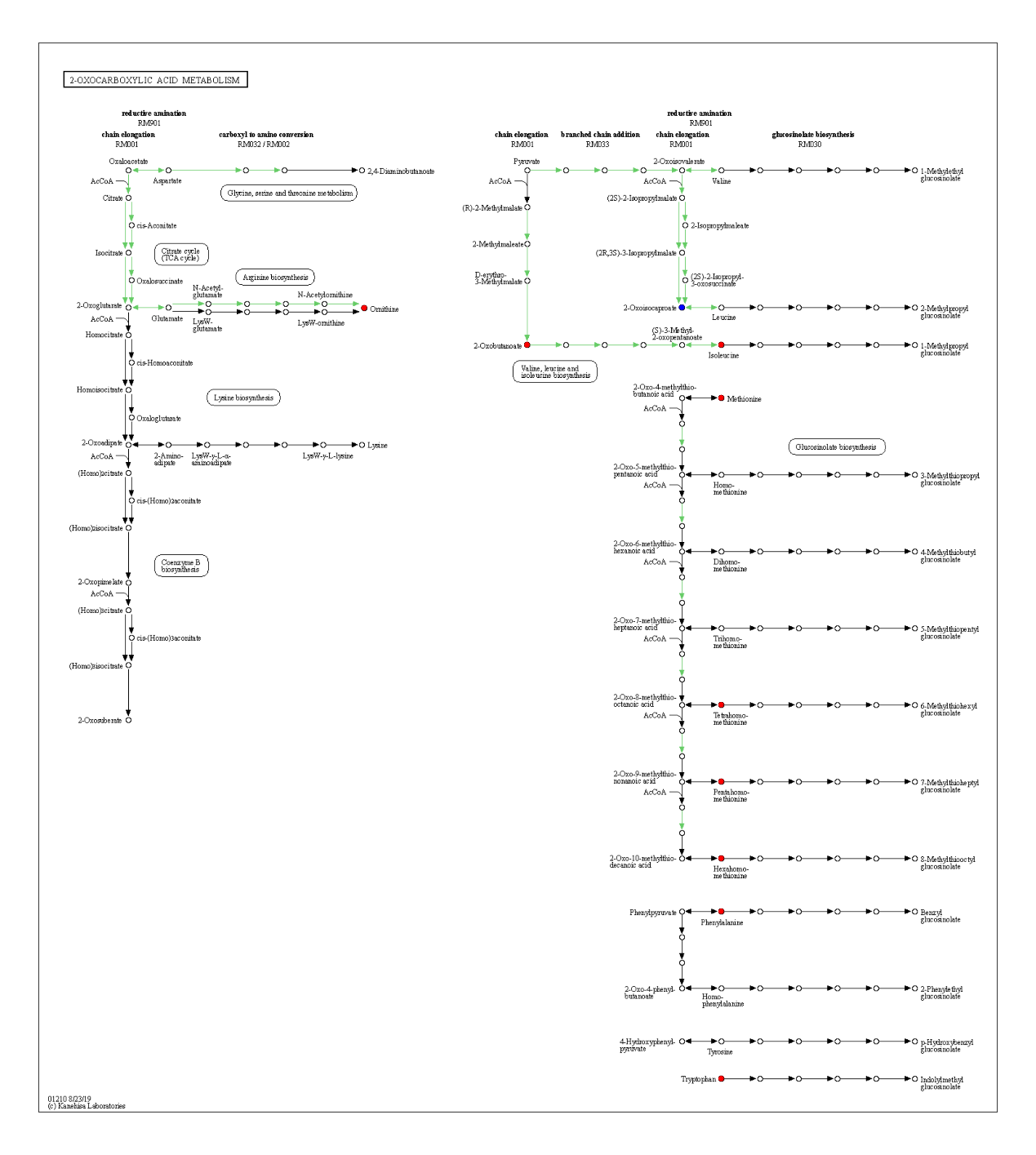


**Figure S9-3.** The 2-oxocarboxylic acid metabolism with significant enrichment of differential metabolites in the KEGG pathway analysis of the WG vs. SCG group.


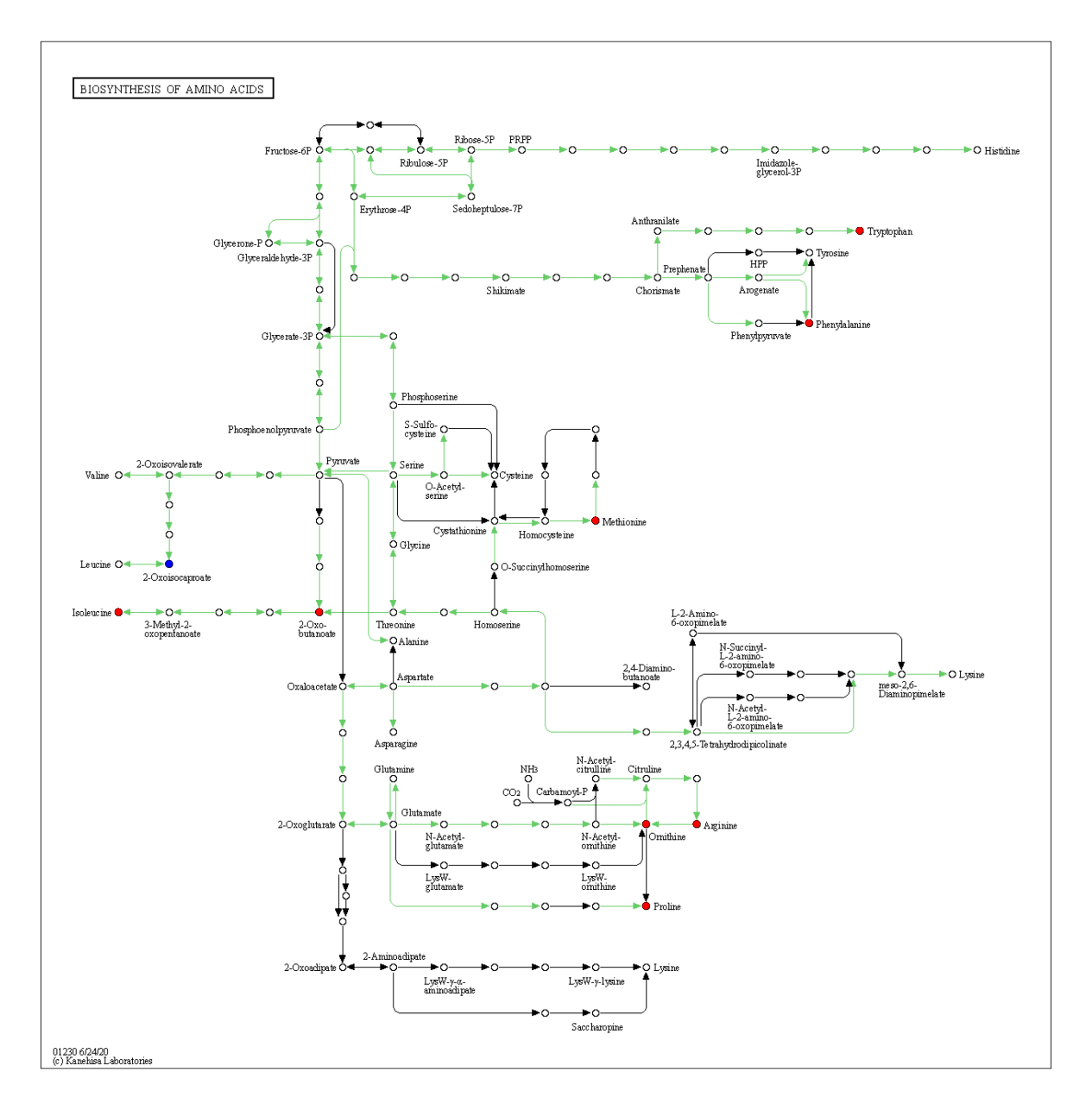


**Figure S9-4.** The biosynthesis of amino acids with significant enrichment of differential metabolites in the KEGG pathway analysis of the WG vs. SCG group.


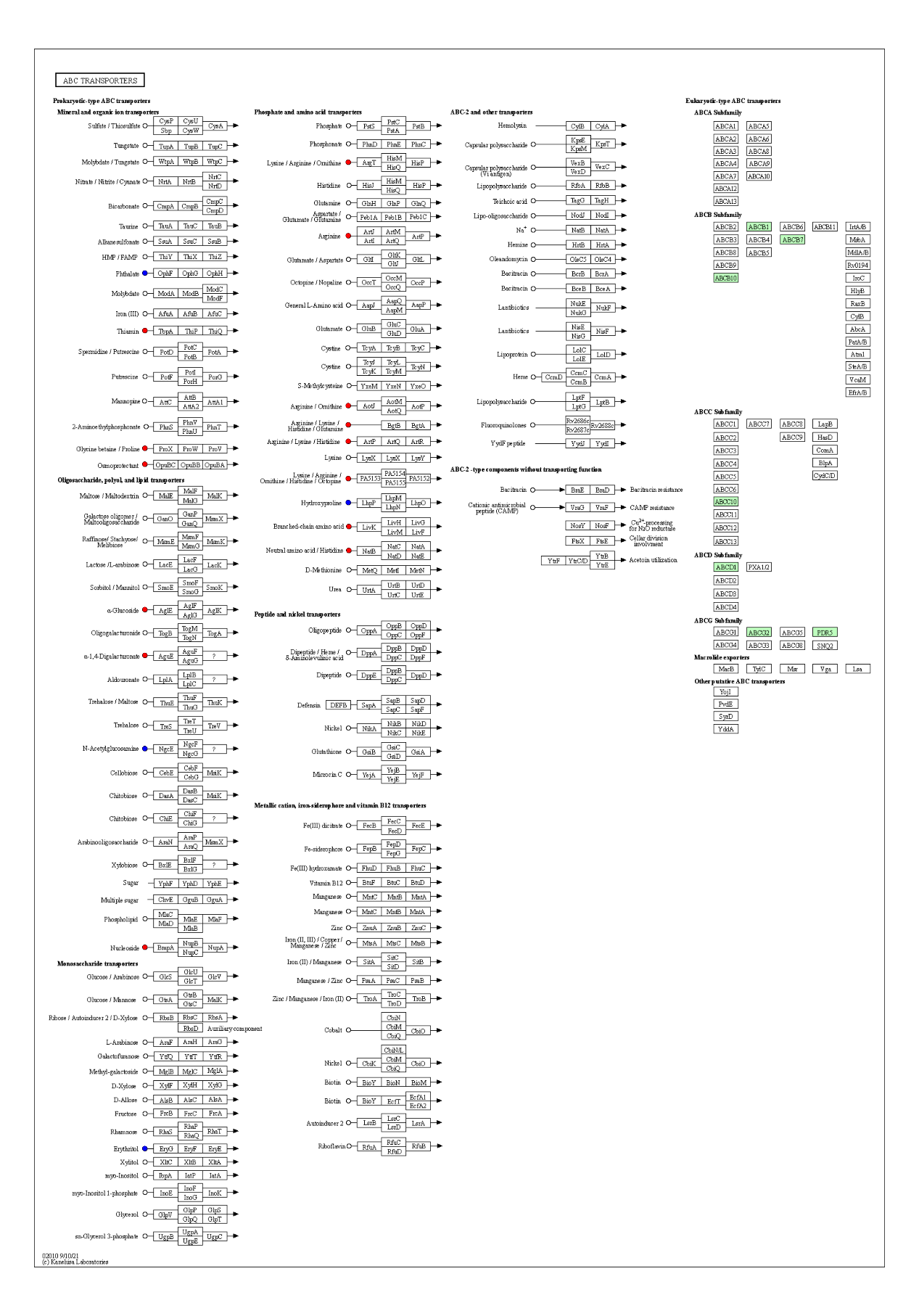


**Figure S9-5.** The ABC transporters with significant enrichment of differential metabolites in the KEGG pathway analysis of the WG vs. SCG group.


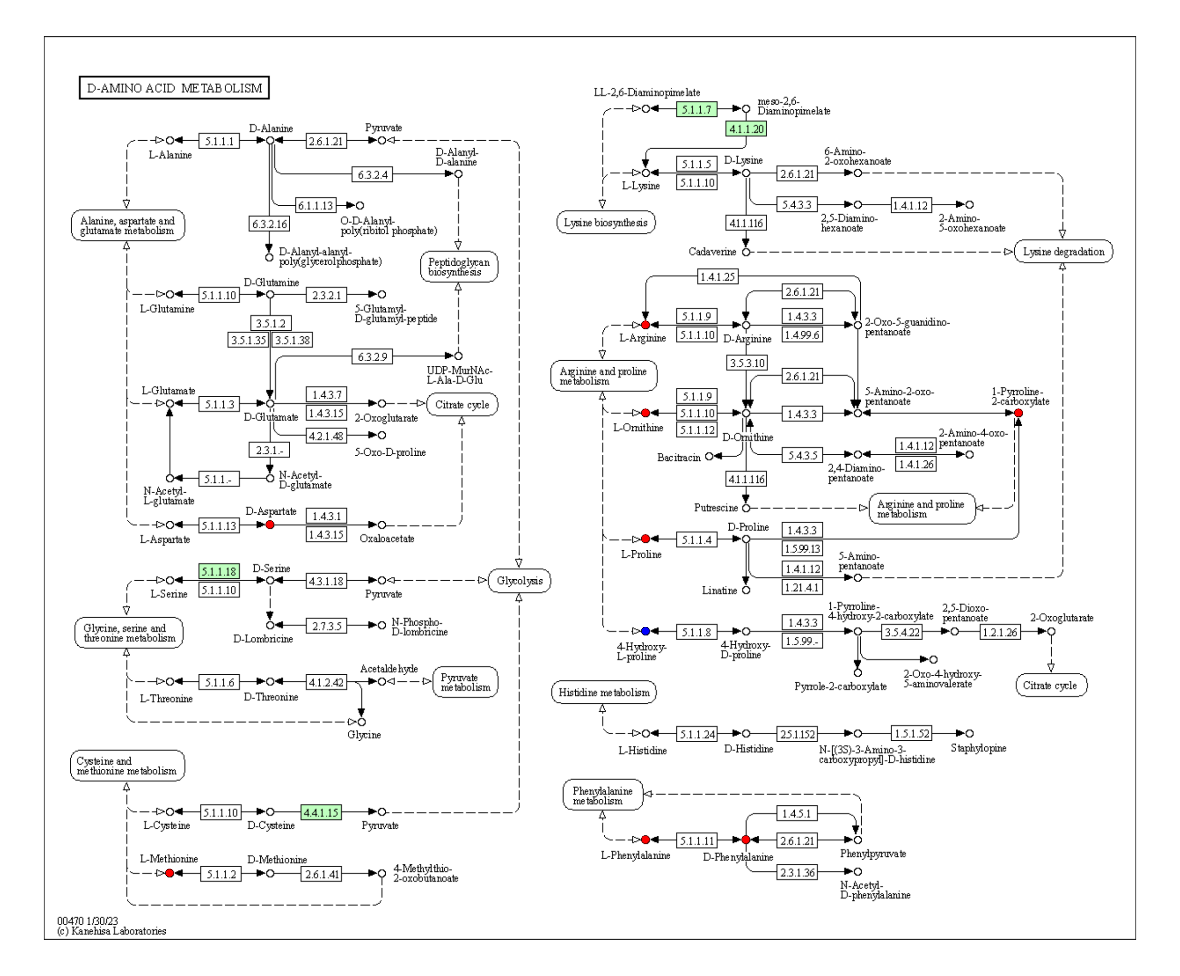


**Figure S9-6.** The aminoacyl-tRNA biosynthesis with significant enrichment of differential metabolites in the KEGG pathway analysis of the WG vs. SCG group.


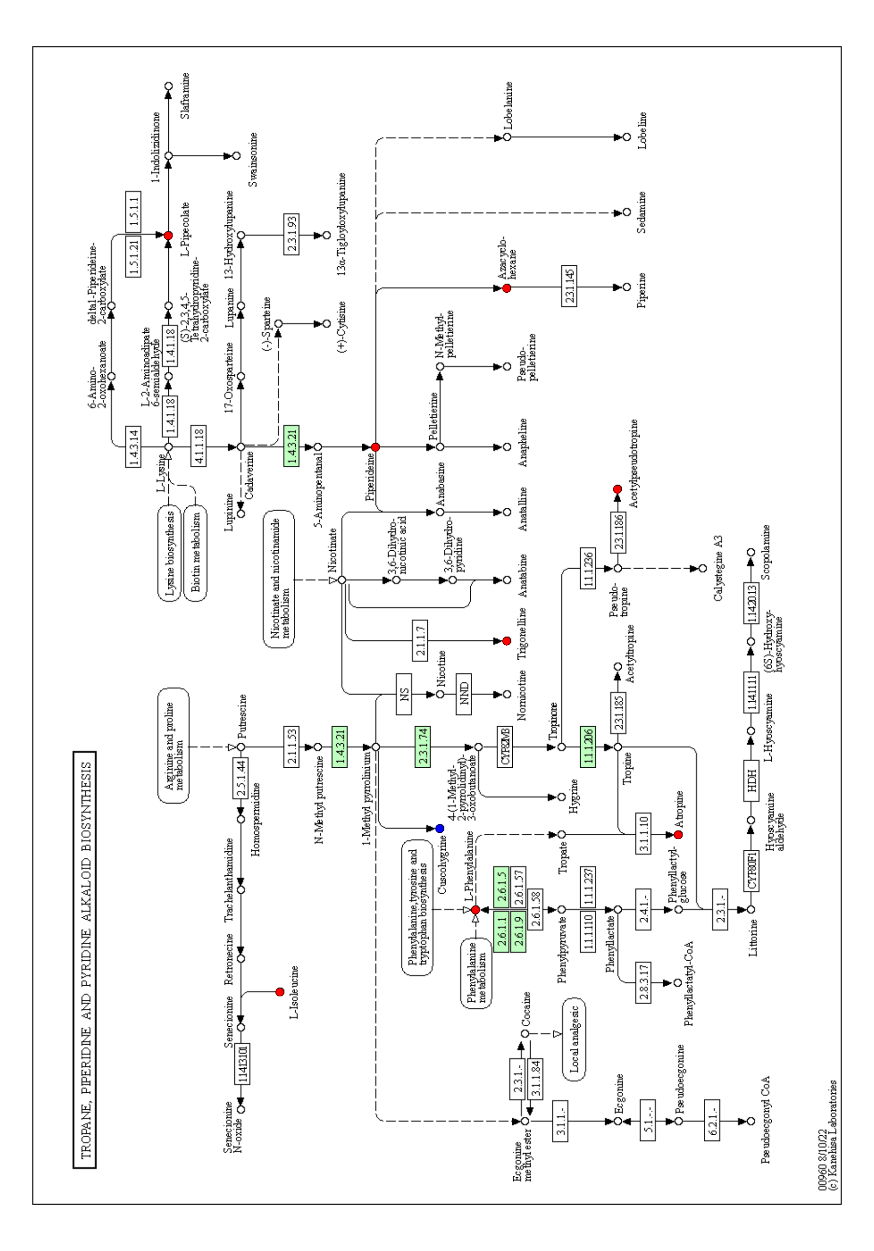


**Figure S9-7.** The tropane, piperidine and pyridine alkaloid biosynthesis with significant enrichment of differential metabolites in the KEGG pathway analysis of the WG vs. SCG group.


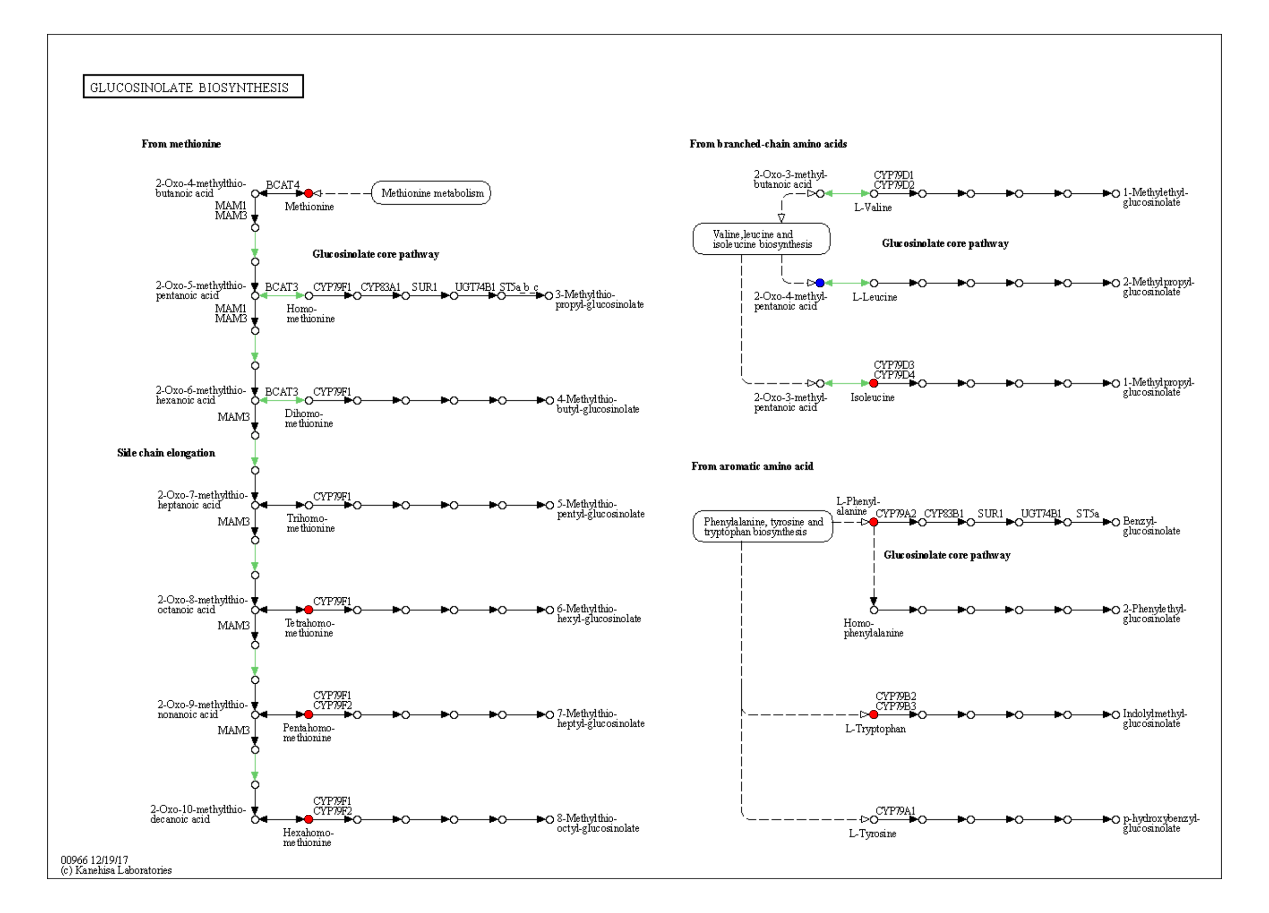


**Figure S9-8.** The glucosinolate biosynthesis with significant enrichment of differential metabolites in the KEGG pathway analysis of the WG vs. SCG group.


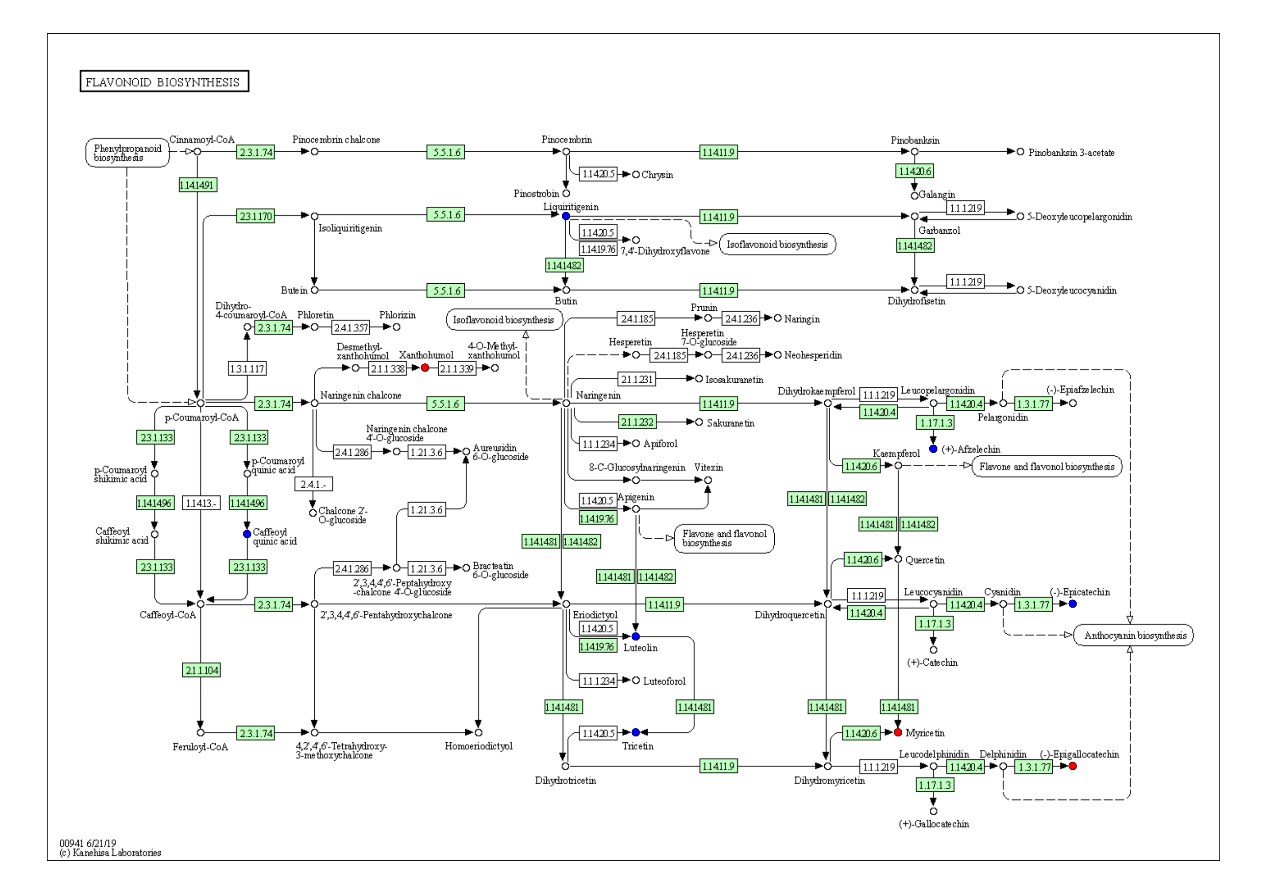


**Figure S10-1.** The flavonoid biosynthesis with significant enrichment of differential metabolites in the KEGG pathway analysis of the WG vs. SBG group.


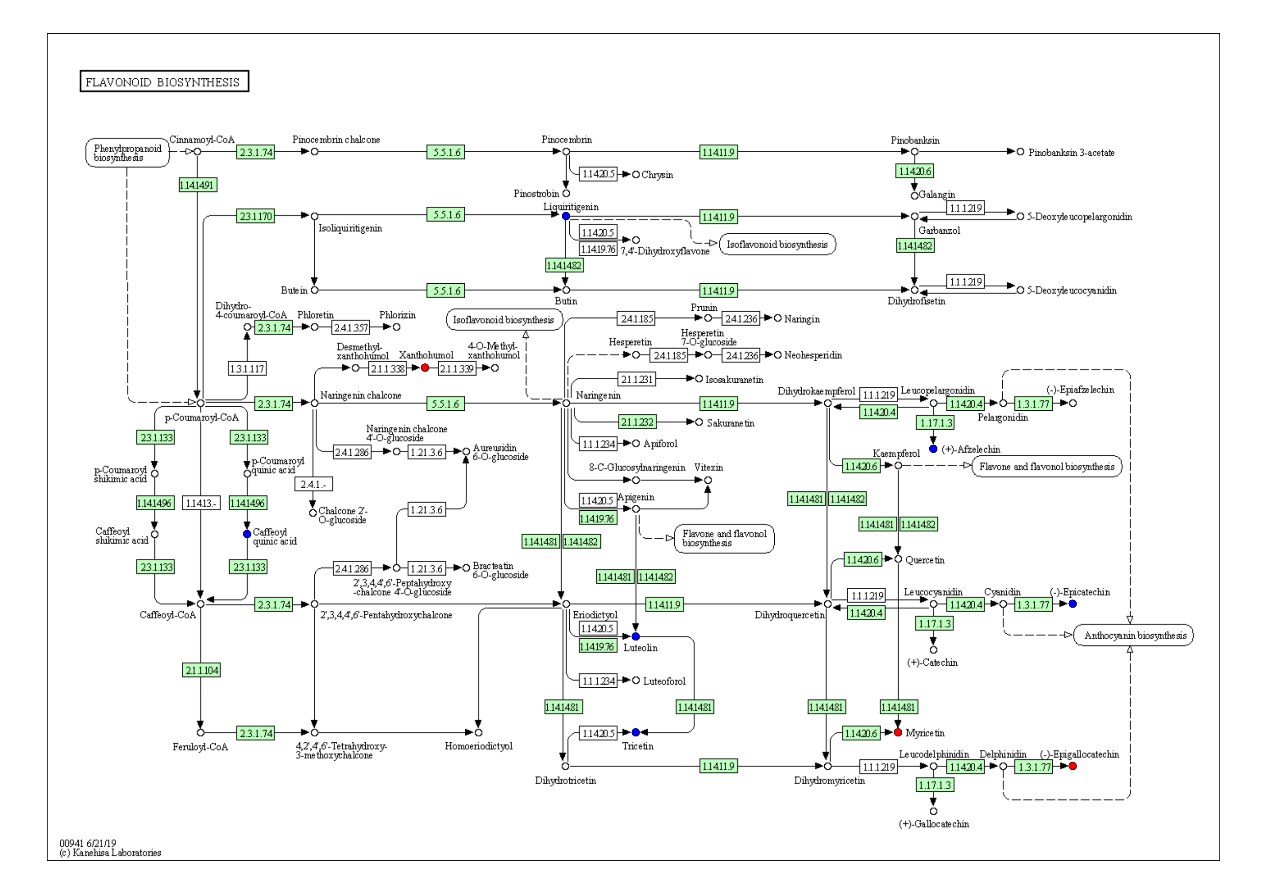


**Figure S10-2.** The phenylpropanoid biosynthesis with significant enrichment of differential metabolites in the KEGG pathway analysis of the WG vs. SBG group.


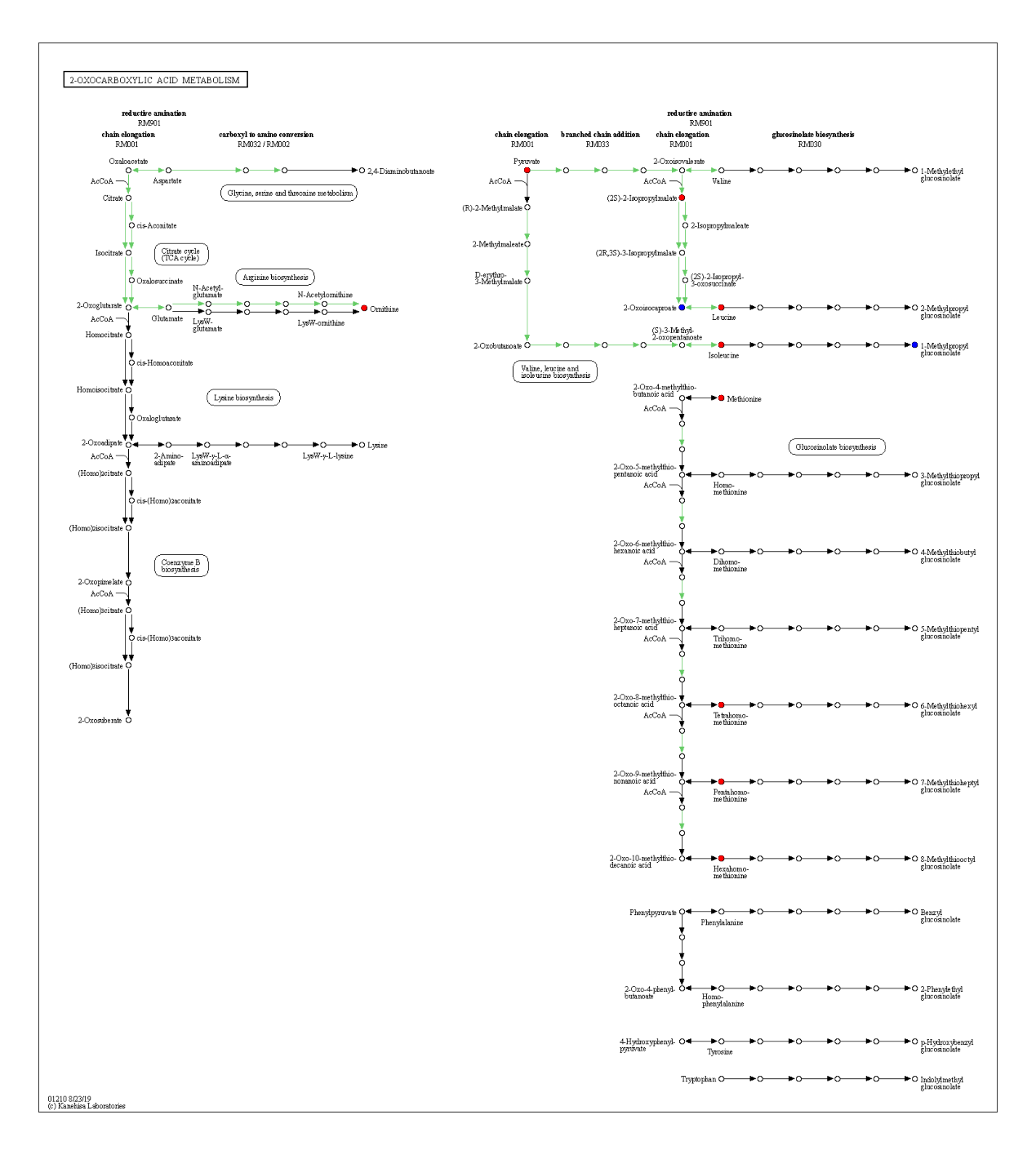


**Figure S10-3.** The 2-oxocarboxylic acid metabolism with significant enrichment of differential metabolites in the KEGG pathway analysis of the WG vs. SBG group.


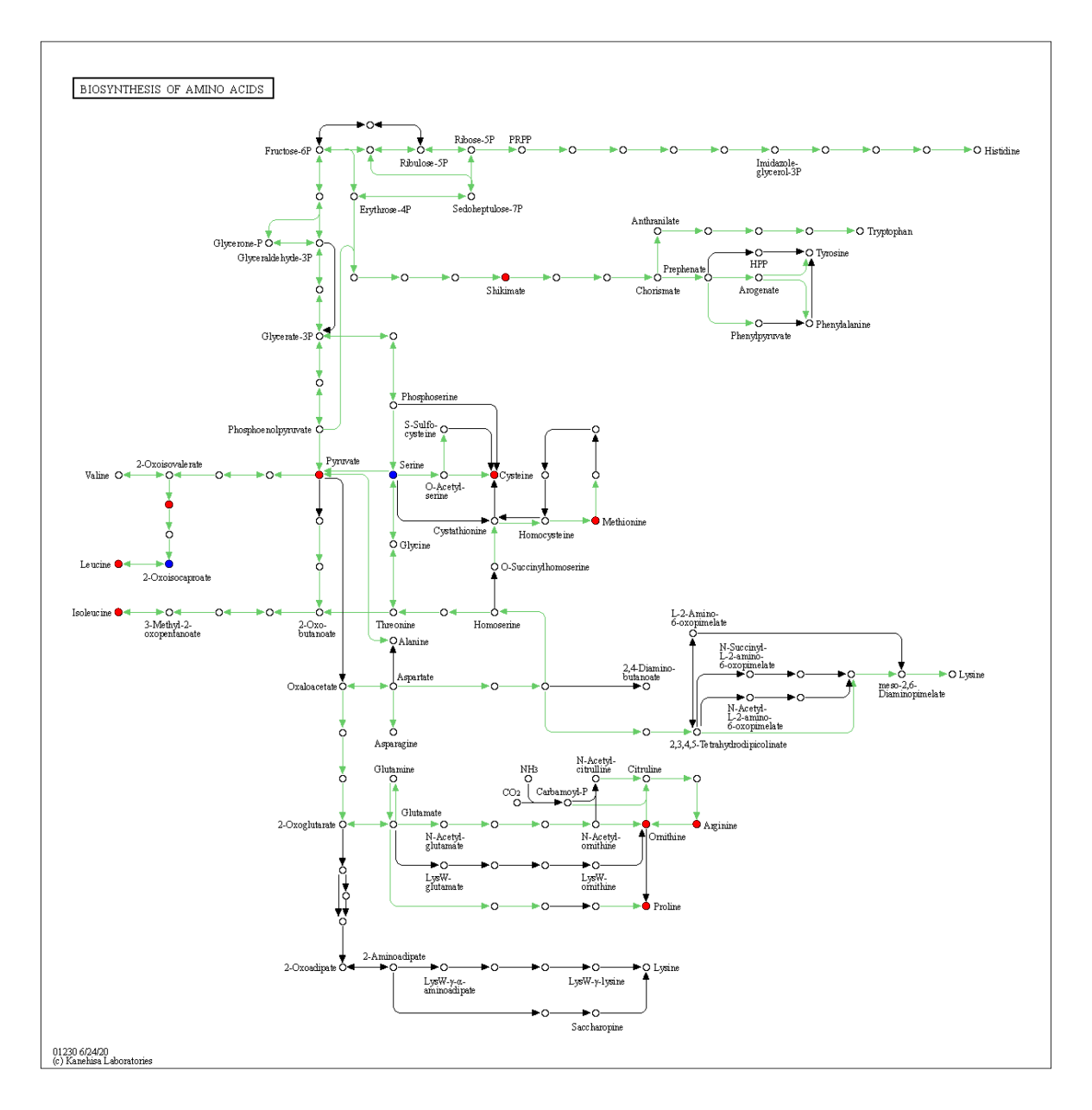


**Figure S10-4.** The biosynthesis of amino acids with significant enrichment of differential metabolites in the KEGG pathway analysis of the WG vs. SBG group.


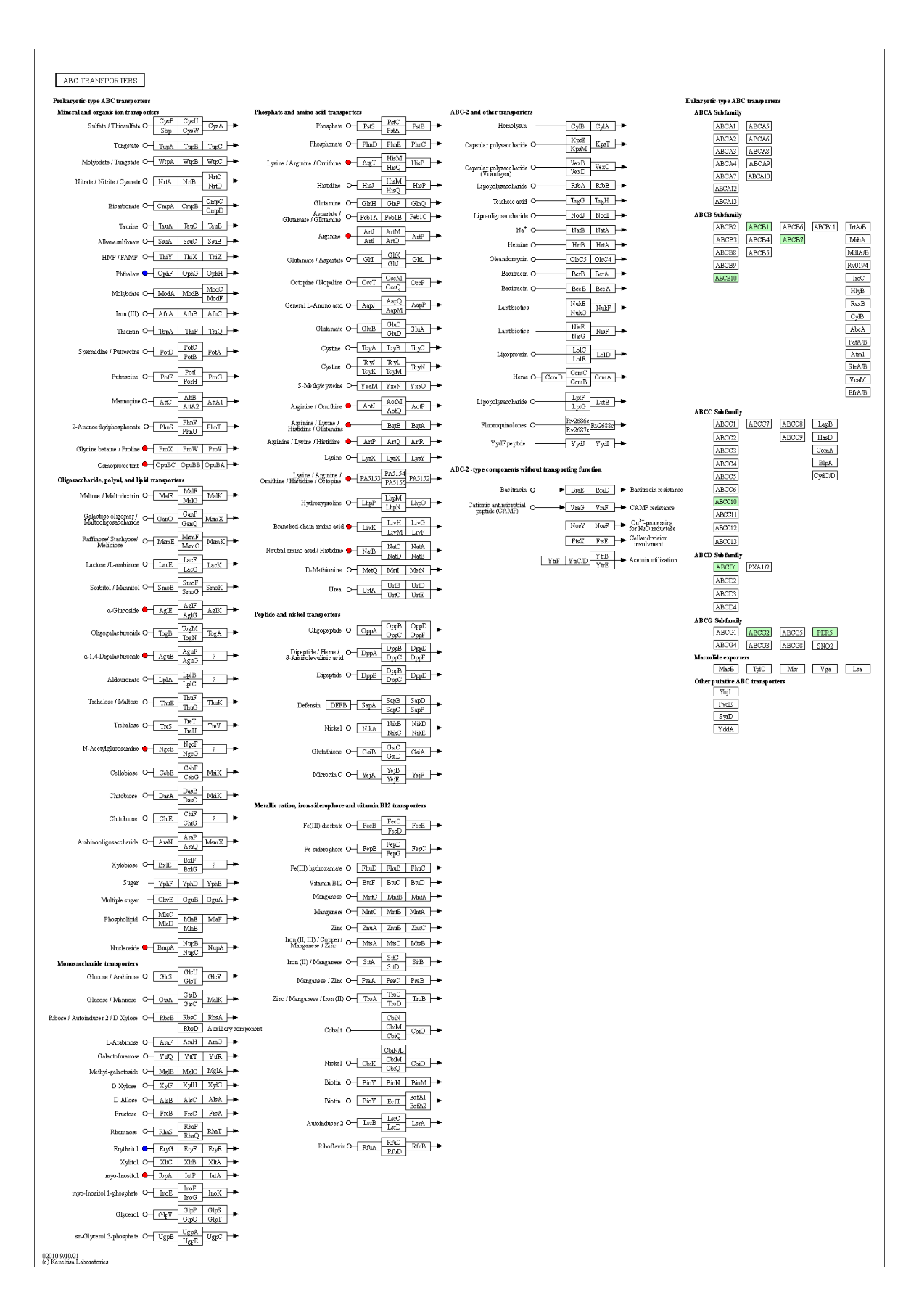


**Figure S10-5.** The ABC transporters with significant enrichment of differential metabolites in the KEGG pathway analysis of the WG vs. SBG group.


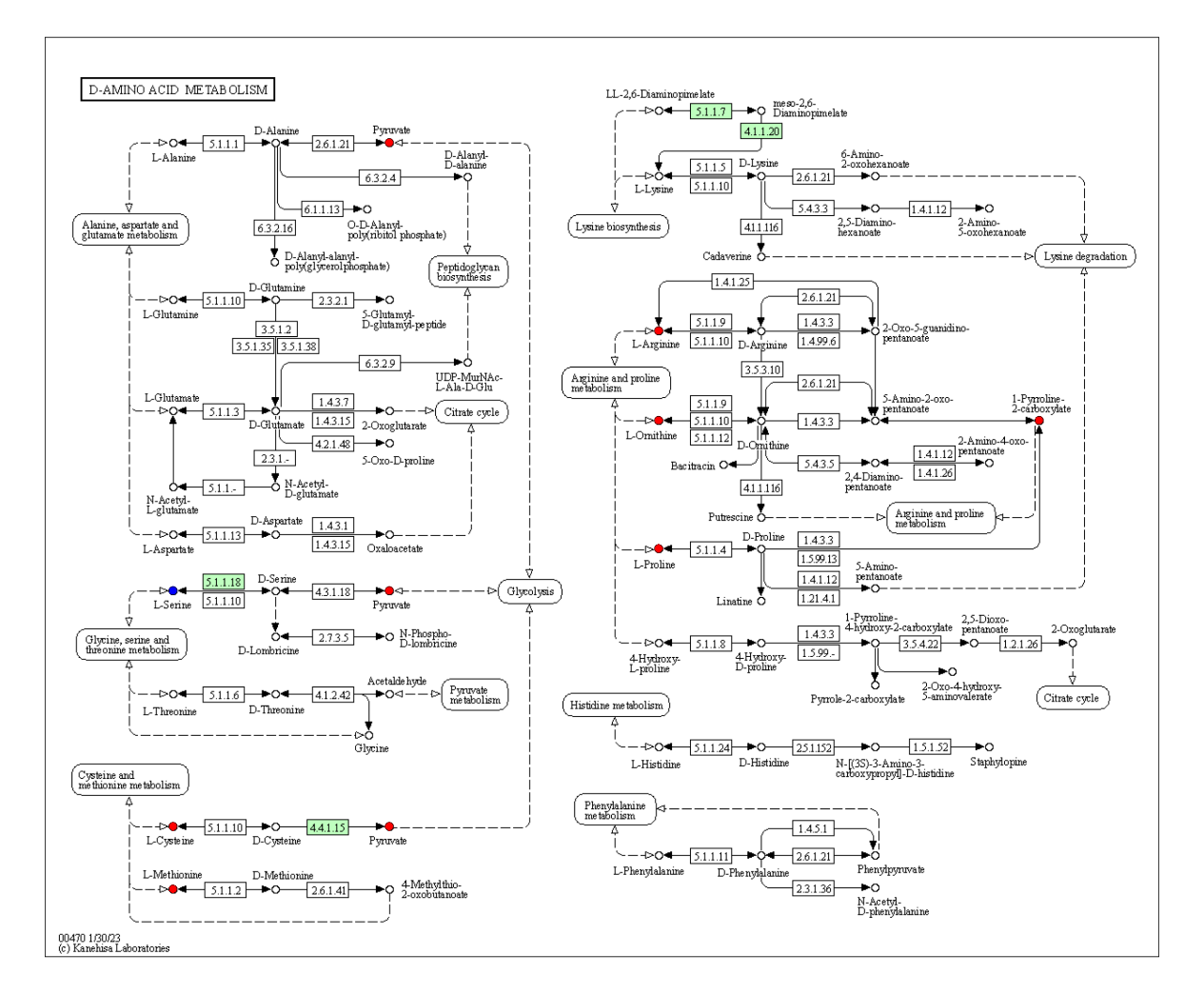


**Figure S10-6.** The D-amino acids metabolism with significant enrichment of differential metabolites in the KEGG pathway analysis of the WG vs. SBG group.


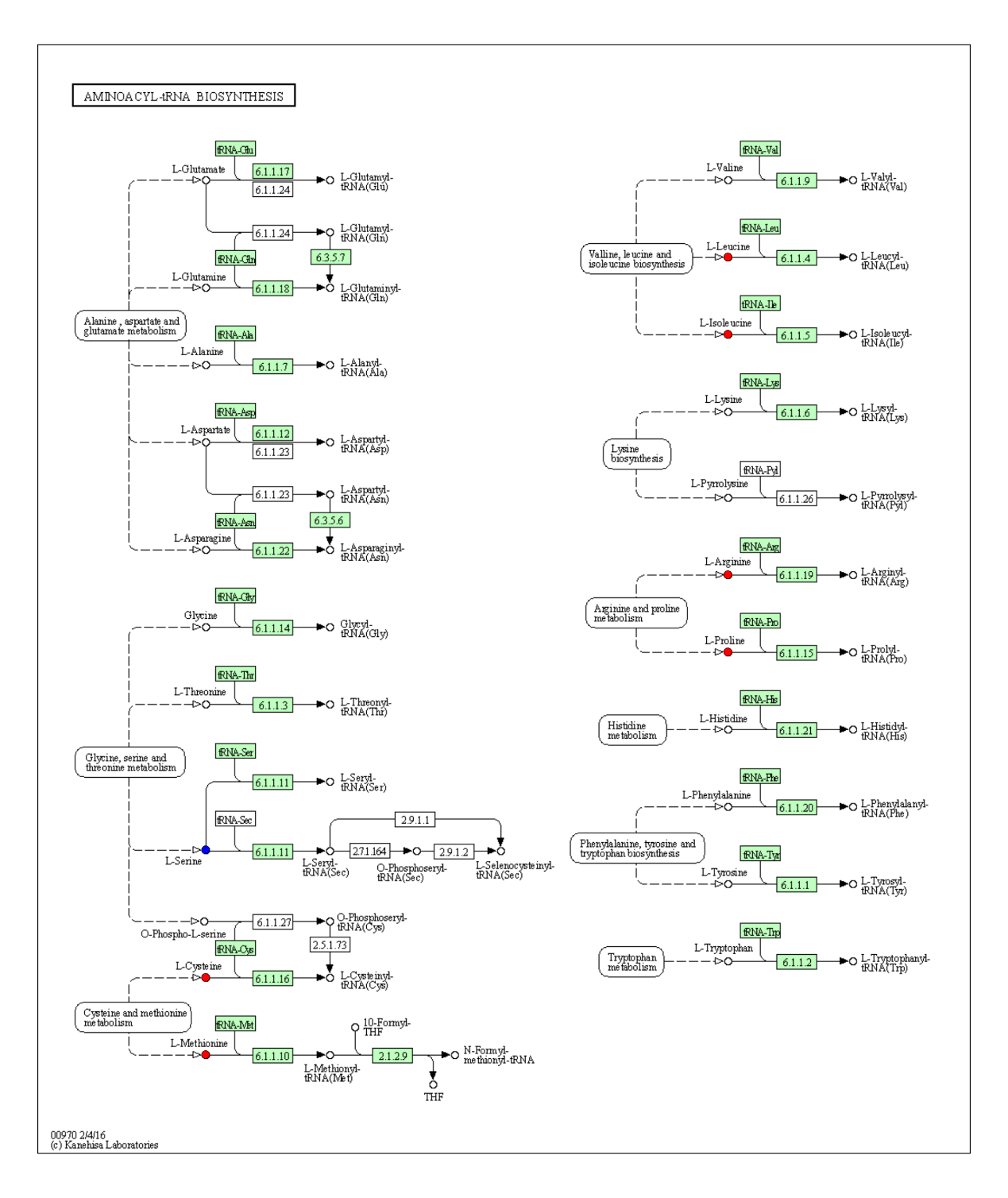


**Figure S10-7.** The aminoacyl-tRNA biosynthesis with significant enrichment of differential metabolites in the KEGG pathway analysis of the WG vs. SBG group.


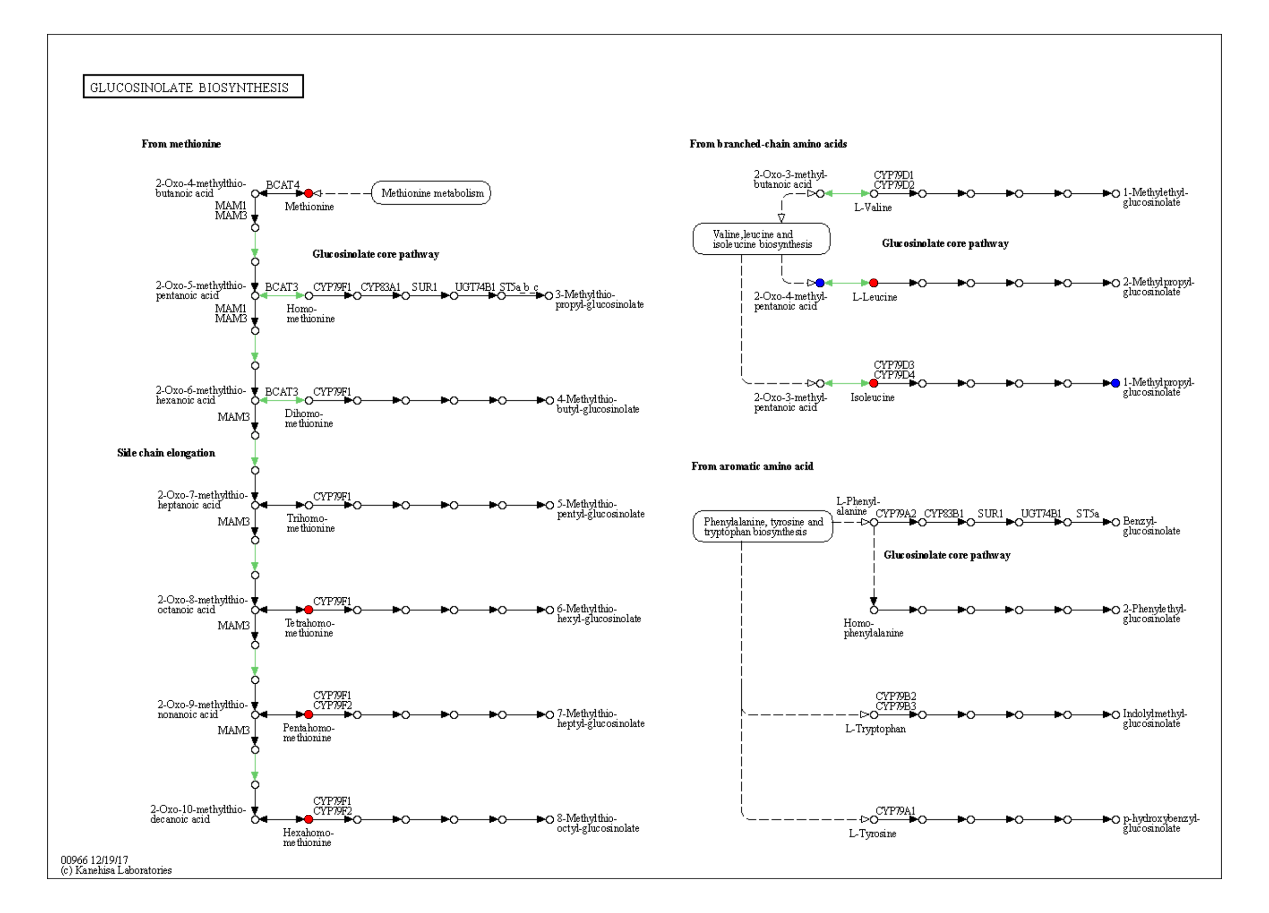


**Figure S10-8.** The glucosinolate biosynthesis with significant enrichment of differential metabolites in the KEGG pathway analysis of the WG vs. SBG group.


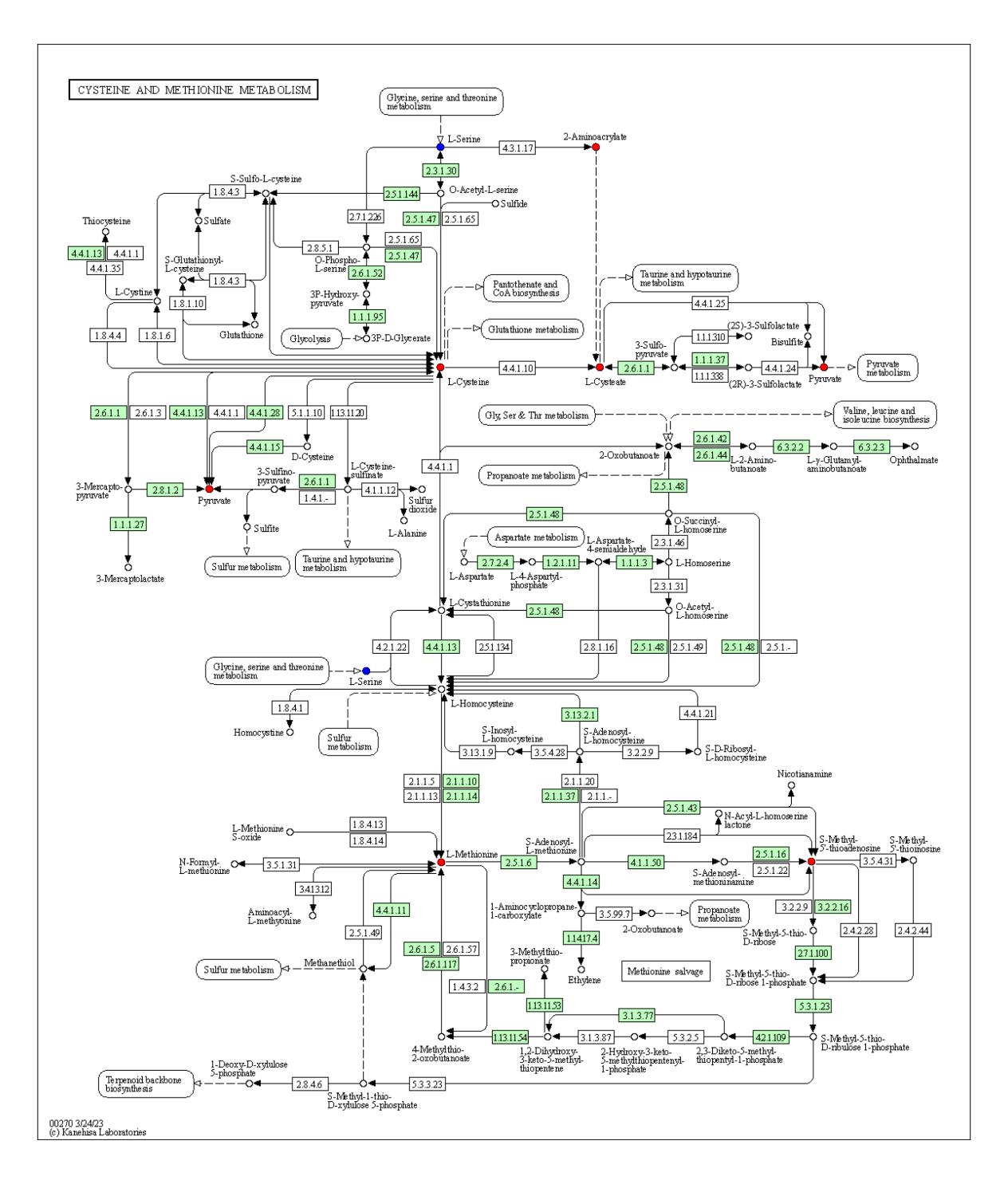


**Figure S10-9.** The cysteine and methionine metabolism with significant enrichment of differential metabolites in the KEGG pathway analysis of the WG vs. SBG group.

| **Table S2** Contents of chemical elements determined in the WG vs. PG group (p<0.05 and VIP>1). | | | | | | |
| --- | --- | --- | --- | --- | --- | --- |
| **NO.** | **Chemical  elements** | **mean1** | **mean2** | **pValue** | **Log2FC** | **data_VIP_select** |
| 1 | **Fe** | 0.002303776 | 0.001816994 | 0.000028 | -0.342446792 | 1.140815292 |
| 2 | **Ca** | 0.102820375 | 0.057097144 | 0.0000873 | -0.84863569 | 1.133086412 |
| 3 | **Ni** | 0.0000108 | 0.00000825 | 0.000116162 | -0.388565288 | 1.129386478 |
| 4 | **K** | 0.702610305 | 0.743389008 | 0.00011905 | 0.08139262 | 1.12772141 |
| 5 | **Cu** | 0.000115782 | 0.000137332 | 0.000255397 | 0.246256847 | 1.109312041 |
| 6 | **Na** | 0.017331083 | 0.02002395 | 0.0005874 | 0.208364784 | 1.08785629 |
| 7 | **Co** | 0.000000555 | 0.000000429 | 0.000976435 | -0.371510124 | 1.082286272 |
| 8 | **Zn** | 0.00049001 | 0.00056274 | 0.00109374 | 0.199657323 | 1.076738913 |
| 9 | **Mo** | 0.0000034 | 0.00000269 | 0.001264382 | -0.337928574 | 1.082337642 |
| 10 | **Cr** | 0.00000742 | 0.00000595 | 0.00140533 | -0.318529518 | 1.083994158 |
| 11 | **Pb** | 0.00000114 | 0.000000841 | 0.001952487 | -0.438856119 | 1.063763245 |
| 12 | **Sr** | 0.000565235 | 0.000364406 | 0.028571429 | -0.633304088 | 1.147951494 |
| 13 | **Ag** | 0.00000751 | 0.000000251 | 0.028571429 | -4.903053638 | 1.063335741 |
| 14 | **Ir** | 0.000000282 | 1.46E-08 | 0.028571429 | -4.271654888 | 1.118026238 |
| 15 | **Li** | 0.0000022 | 0.00000161 | 0.028571429 | -0.450442835 | 1.137671028 |
| 16 | **Cd** | 0.00000134 | 0.000000353 | 0.028571429 | -1.924492912 | 1.170290818 |
| 17 | **Hg** | 0.00000453 | 0.000000332 | 0.028571429 | -3.770255904 | 1.077390371 |

| **Table S3** Contents of chemical elements determined in the WG vs. HG group (p<0.05 and VIP>1). | | | | | | |
| --- | --- | --- | --- | --- | --- | --- |
| **NO.** | **Chemical  elements** | **mean1** | **mean2** | **pValue** | **Log2FC** | **data_VIP_select** |
| 1 | **Na** | 0.017389817 | 0.02002395 | 0.000275902 | 0.203483843 | 1.177681528 |
| 2 | **Mn** | 0.00026284 | 0.000302868 | 0.000308157 | 0.204504309 | 1.183222357 |
| 3 | **K** | 0.727521043 | 0.743389008 | 0.000969536 | 0.031128379 | 1.154907768 |
| 4 | **As** | 0.00000106 | 0.00000145 | 0.001283001 | 0.451988635 | 1.147776281 |
| 5 | **V** | 0.00000156 | 0.00000116 | 0.001601332 | -0.427421224 | 1.146260331 |
| 6 | **Cd** | 0.000000296 | 0.000000353 | 0.002041114 | 0.254071008 | 1.121296578 |
| 7 | **Sr** | 0.000400141 | 0.000364406 | 0.002670779 | -0.134961747 | 1.118056755 |
| 8 | **Zr** | 0.000000876 | 0.000000693 | 0.002988282 | -0.338075517 | 1.119116936 |
| 10 | **Ca** | 0.106181023 | 0.057097144 | 0.028571429 | -0.895035457 | 1.230110045 |
| 11 | **Zn** | 0.000468026 | 0.00056274 | 0.028571429 | 0.265879838 | 1.237197196 |
| 12 | **P** | 0.094460228 | 0.123936928 | 0.028571429 | 0.391827191 | 1.230135294 |
| 13 | **Cu** | 0.000104771 | 0.000137332 | 0.028571429 | 0.390428387 | 1.23157169 |
| 14 | **Ag** | 0.00000187 | 0.000000251 | 0.028571429 | -2.897279001 | 1.215839046 |
| 15 | **Ir** | 9.34E-08 | 1.46E-08 | 0.028571429 | -2.677454181 | 1.237460891 |
| 16 | **Mo** | 0.00000169 | 0.00000269 | 0.028571429 | 0.670582926 | 1.213086744 |
| 17 | **Hg** | 0.00000146 | 0.000000332 | 0.028571429 | -2.136713222 | 1.234753328 |
| 18 | **Ti** | 0.000132322 | 0.000166457 | 0.028571429 | 0.331096596 | 1.058620926 |
| 19 | **Tl** | 5.13E-08 | 0.000000108 | 0.028571429 | 1.074000581 | 1.240044627 |

| **Table S4** Contents of chemical elements determined in the WG vs. SCG group (p<0.05 and VIP>1). | | | | | | |
| --- | --- | --- | --- | --- | --- | --- |
| **NO.** | **Chemical  elements** | **mean1** | **mean2** | **pValue** | **Log2FC** | **data_VIP_select** |
| 1 | **Sr** | 0.000289421 | 0.000364406 | 0.000183045 | 0.332377108 | 1.18781101 |
| 2 | **Na** | 0.023797055 | 0.02002395 | 0.000395658 | -0.24905645 | 1.168901579 |
| 3 | **Al** | 0.001451027 | 0.000621549 | 0.000675544 | -1.223134328 | 1.154232009 |
| 4 | **Sn** | 0.00000772 | 0.00000951 | 0.001780826 | 0.300844494 | 1.127299447 |
| 5 | **Ag** | 0.000000311 | 0.000000251 | 0.002458221 | -0.309227216 | 1.130991464 |
| 6 | **Li** | 0.00000141 | 0.00000161 | 0.003191538 | 0.191365526 | 1.097991931 |
| 7 | **P** | 0.115123351 | 0.123936928 | 0.006619496 | 0.106425622 | 1.065502617 |
| 8 | **Fe** | 0.001569382 | 0.001816994 | 0.007480483 | 0.211357097 | 1.068075995 |
| 9 | **V** | 0.00000144 | 0.00000116 | 0.007522182 | -0.311944006 | 1.051744485 |
| 10 | **Pb** | 0.000000612 | 0.000000841 | 0.008333024 | 0.458574148 | 1.038963765 |
| 11 | **Ca** | 0.068576535 | 0.057097144 | 0.009428092 | -0.264296427 | 1.033927077 |
| 12 | **Ti** | 0.000221913 | 0.000166457 | 0.009430589 | -0.414844644 | 1.038951558 |
| 13 | **Zr** | 0.000000579 | 0.000000693 | 0.01311817 | 0.259292004 | 1.012769793 |
| 14 | **Co** | 0.000000356 | 0.000000429 | 0.014106561 | 0.269100407 | 1.01238849 |
| 15 | **Zn** | 0.000508953 | 0.00056274 | 0.028571429 | 0.14493608 | 1.184178274 |
| 16 | **Cu** | 0.0000661 | 0.000137332 | 0.028571429 | 1.054945653 | 1.235791718 |
| 17 | **Mo** | 0.00000432 | 0.00000269 | 0.028571429 | -0.68342514 | 1.22519049 |
| 18 | **Cd** | 0.0000014 | 0.000000353 | 0.028571429 | -1.987686739 | 1.237551846 |
| 19 | **Tl** | 0.000000048 | 0.000000108 | 0.028571429 | 1.169925001 | 1.230601621 |

| **Table S5** Contents of chemical elements determined in the WG vs. SCG group (p<0.05 and VIP>1). | | | | | | |
| --- | --- | --- | --- | --- | --- | --- |
| **NO.** | **Chemical  elements** | **mean1** | **mean2** | **pValue** | **Log2FC** | **data_VIP_select** |
| 1 | Cu | 0.000160345 | 0.000137332 | 0.000181555 | -0.223511537 | 1.25889515 |
| 2 | Fe | 0.002196484 | 0.001816994 | 0.000185959 | -0.273642335 | 1.279876891 |
| 3 | Se | 0.00000128 | 0.00000064 | 0.00022035 | -1 | 1.260720623 |
| 4 | Pb | 0.000000507 | 0.000000841 | 0.000877473 | 0.730120053 | 1.226083084 |
| 5 | Hg | 0.000000496 | 0.000000332 | 0.003365488 | -0.579156879 | 1.157822025 |
| 6 | Ag | 0.000000834 | 0.000000251 | 0.005789666 | -1.732360019 | 1.120599982 |
| 7 | Mn | 0.000339535 | 0.000302868 | 0.014258128 | -0.164871142 | 1.075177691 |
| 8 | Na | 0.008867816 | 0.02002395 | 0.028571429 | 1.175075853 | 1.301108171 |
| 9 | P | 0.16410111 | 0.123936928 | 0.028571429 | -0.404978883 | 1.2864718 |
| 10 | Li | 0.00000392 | 0.00000161 | 0.028571429 | -1.283792966 | 1.293310713 |
| 11 | Mo | 0.00000877 | 0.00000269 | 0.028571429 | -1.70497067 | 1.320840337 |
| 12 | Ni | 0.0000168 | 0.00000825 | 0.028571429 | -1.025995209 | 1.301153457 |
| 13 | Co | 0.000000525 | 0.000000429 | 0.028571429 | -0.291339775 | 1.228679184 |
| 14 | Sn | 0.0000114 | 0.00000951 | 0.028571429 | -0.261516578 | 1.011931248 |
